# ICU admission and mortality in adult patients with influenza A(H1N1)pdm09-related pneumonia in Vietnam since the 2009 H1N1 pandemic: A 10-year cohort study

**DOI:** 10.1371/journal.pone.0348450

**Published:** 2026-08-24

**Authors:** Quang Minh Ho, Bich Thuy Duong, Le Nhu Tung Nguyen, Tri Nugraha Susilawati, Ngoc Minh Tam Bui, Thanh Truc Thai, David J. Muscatello, Anthony Sunjaya, Siyu Chen, Thanh Nguyen Nguyen, Minh Thu Nguyen, Kim Anh Nguyen, Minh Cuong Duong

**Affiliations:** 1 Hospital for Tropical Diseases, Ho Chi Minh City, Vietnam; 2 Franco-Vietnamese Hospital, Ho Chi Minh City, Vietnam; 3 Faculty of Medicine, Universitas Sebelas Maret, Jawa Tengah, Indonesia; 4 University Medical Center Ho Chi Minh City, Ho Chi Minh City, Vietnam; 5 Department of Medical Statistics and Informatics, University of Medicine and Pharmacy at Ho Chi Minh City, Vietnam; 6 School of Population Health, Sydney, New South Wales Australia; 7 The George Institute for Global Health, Sydney, New Souht Wales Australia; 8 Centre for Health Behaviours Research, Jockey Club School of Public Health and Primary Care, Faculty of Medicine, The Chinese University of Hong Kong, Hong Kong SAR, China; 9 School of Nursing, Yale University, New Haven, Connecticut, United States of America; 10 Tam Anh Hospital, Ho Chi Minh City, Vietnam; 11 Oxford University Clinical Research Unit (OUCRU), Ho Chi Minh City, Vietnam; Children's National Hospital, George Washington University, UNITED STATES OF AMERICA

## Abstract

**Background:**

The A(H1N1)pdm09 virus remains an important cause of severe influenza worldwide. This study examined the burden of ICU admission, mortality, and associated predictors among adults with A(H1N1)pdm09-related pneumonia treated at a leading infectious diseases hospital in Vietnam.

**Methods:**

Demographic, clinical, laboratory, treatment, and outcome data were retrospectively collected from the medical records of adults hospitalized with laboratory-confirmed A(H1N1)pdm09 infection between 2009 and 2019.

**Results:**

Among 729 patients with laboratory-confirmed A(H1N1)pdm09 infection, 21.7% (158/729) developed pneumonia. Among patients with pneumonia, 36.7% developed moderate-to-severe acute respiratory distress syndrome, 15.2% required invasive ventilation, 48.7% were admitted to the ICU, and 8.2% died. Independent predictors of ICU admission included age older than 60 years (AOR 18.91, 95% CI 3.60–99.24), the presence of comorbidities (AOR 9.39, 95% CI 2.75–32.06), and laboratory evidence of systemic inflammation, renal dysfunction, and hepatic involvement. Intensified antiviral therapy (AOR = 8.577, 95%CI 1.416–83.341, P = 0.020), and invasive ventilation (AOR 8.426, 95%CI 1.105–141.366, P = 0.039) were independently associated with mortality.

**Conclusions:**

Mortality associated with A(H1N1)pdm09-related pneumonia remains substantial. Older patients, those with comorbidities, and those presenting with abnormal laboratory findings should be monitored closely for deterioration requiring intensive care. The study did not demonstrate improved outcomes with intensified antiviral regimens. Prospective studies are required before definitive treatment recommendations can be made.

## Introduction

The A(H1N1)pdm09 virus emerged in 2009 and triggered a global influenza pandemic associated with an estimated 123,000–203,000 deaths [1,2]. The pandemic was declared over in August 2010, after which A(H1N1)pdm09 transitioned into an endemic seasonal strain co-circulating with influenza A(H3N2) and B viruses [3,4]. While influenza seasonality is well defined in temperate regions, circulation patterns in tropical areas are often irregular, which may compromise the optimal timing and impact of vaccination programs [5]. Despite the availability of effective vaccines, A(H1N1)pdm09 remains a significant contributor to worldwide influenza-related morbidity and mortality [6]. From October 2024 through May 2025 influenza activity was reported in all influenza transmission zones [7]. The predominant viruses varied across transmission zones and between countries [7]. A study conducted in China found that between 2010 and 2015, the influenza-associated mortality was 9.9 per 100,000 people, in which influenza A(H3N2) virus was the leading cause with a rate of 5.18 per 100,000, followed by A(H1N1)pdm09 with a rate of 2.9 per 100,000 [8]. Additionally, the prevalence of influenza shifted between the Southern and Northern Hemispheres following the COVID-19 pandemic. Specifically, the post-pandemic peak of influenza in the Northern Hemisphere occurred as early as December, with the epidemic’s duration increasing to 11 weeks. Meanwhile, in the Southern Hemisphere, the epidemic increased in length to 20 weeks [9]. Given that influenza A/B and SARS-CoV-2 share similar transmission pathways (via aerosolized droplets and contaminated surfaces) and clinical presentations, and typically circulate in overlapping seasonal outbreaks, their potential for co-infection poses a significant public health threat [9].

While most influenza infections resolve without treatment, some patients, especially high-risk groups, may develop serious complications that can lead to death [3]. High-risk groups include children younger than five years, the elderly, pregnant women, and individuals with compromised immune systems or chronic medical conditions [10,11]. However, the literature reports inconsistent risk factors for severe outcomes, which may partly explain the observed between-country differences in A(H1N1)pdm09 mortality burden [12,13]. A study involving 696 A(H1N1)pdm09 patients in Spain has concurred that immune deficiency and chronic cardiovascular diseases are risk factors for mortality. Interestingly, this study has found that older age is associated with lower ICU admission but a risk factor for death, suggesting that age may play a differential role in predicting ICU admission versus mortality [11]. Regarding pregnancy, given that this is a well-accepted risk factor for severity and mortality of A(H1N1)pdm09 pneumonia, the World Health Organization (WHO) recommends that pregnant women are prioritized for influenza vaccination [14]. Although some studies reported differing mortality estimates among pregnant women [12], pregnancy remains an established high-risk condition for severe influenza outcomes.

The dissimilarities in identifying risk factors for A(H1N1)pdm09 pneumonia-related mortality worldwide may reflect differences in study design, timing, population age structures, ethnic composition, geographical settings, and vaccination coverage [15]. Frequent viral mutations that influence disease severity may further contribute to variability in findings [16]. Therefore, ongoing, robust, country-specific studies are needed to improve understanding of the risk factors of A(H1N1)pdm09infection-related ICU admission and mortality. However, long-term influenza surveillance remains limited in tropical regions of the Asia-Pacific due to gaps in information technology and available resources, with surveillance systems largely focusing on basic epidemiological data but lacking clinical and outcome information [17,18]. Most studies in these areas are short-term (typically 1–2 years) [19], which is insufficient for effective monitoring, as influenza circulation in tropical settings tends to be sporadic and unpredictable. Establishing a comprehensive, nationwide surveillance system requires substantial financial investment, trained personnel, and infrastructure capable of collecting and integrating individual-level data. Moreover, because many countries in the region face competing public health priorities [20], maintaining long-term influenza surveillance systems is often challenging and may not be feasible or sustainable [19,21]. In Vietnam, influenza viruses co-circulated all year round and were an important cause of influenza-like illness between 2006 and 2010 [22]. The first H1N1 vaccine became available locally in 2010, followed by other vaccines targeting both seasonal influenza strains and A(H1N1)pdm09. However, these vaccines are accessible primarily on a self-paid basis, even for high-risk groups. To strengthen influenza prevention and control in Vietnam and similar settings, continual assessment of disease severity is needed [22]. This study aimed to examine the clinical burden and identify predictors of severe disease among adult patients with A(H1N1)pdm09-related pneumonia. The primary outcomes were ICU admission and all-cause mortality. The secondary outcomes included respiratory support requirements (such as invasive ventilation), development of moderate-to-severe ARDS, occurrence of hospital-acquired infections, and overall hospital and ICU lengths of stay.

## Materials and methods

### Study design and context

A 10-year retrospective cohort study was conducted at the Hospital for Tropical Diseases (HTD) in Ho Chi Minh City from 1st January 2009–31st December 2019. The study was approved by the Ethics Committee of HTD (approval number 43/HĐĐĐ). Data were accessed for research purposes from 07/11/2019 to 30/04/2020. HTD is the 550-bed referral hospital for infectious diseases in southern Vietnam, receiving patients from across this region, including the Mekong Delta, with approximately 3,000 patients accessing healthcare services daily [23]. HTD’s medical records of hospitalized patients are standardized in accordance with the Vietnam Ministry of Health’s guidelines [24].

All eligible hospitalized patients meeting the inclusion criteria during the study period were enrolled consecutively. The inclusion criteria included persons aged 17 years and older (referred to here as ‘adults’, according to the hospital admission policy and national clinical practice during the study period.), having a positive reverse transcription polymerase chain reaction (RT-PCR) result with A(H1N1)pdm09 from nasal or oropharyngeal swab, and developing pneumonia confirmed by chest x-rays. There were no exclusion criteria.

### A(H1N1)pdm09 laboratory confirmation, definitions of A(H1N1)pdm09 pneumonia, and indications for ICU admission

Nasal and oropharyngeal swabs were collected by medical staffs as per the HTD’s guidelines. Loop Mediated Isothermal Amplification (LAMP) has also been reported as a rapid molecular method for detecting influenza A virus [25]. However, throughout the study period, real-time RT PCR remained the routine diagnostic method at our institution and was therefore used for patient enrolment. RT-PCR was performed on the LightCycler 480 II System (Roche Molecular Diagnostics, Pleasanton, CA) [26,27] using the QIAGEN OneStep RT‐PCR kit [27,28]. The 20 µl of reaction volume was prepared and included 5 µl of extracted RNA template, 0.5 µl of enzyme mix, 10 µl of 2 × reaction mix, 0.25 µmol/L influenza A forward primer, 0.2 µmol/L influenza A reverse primer, 0.25 µmol/L influenza A probe, 0.25 µmol/L influenza B forward primer, 0.2 µmol/L influenza B reverse primer, and 0.25 µmol/L influenza B original probe. This process had been validated elsewhere [29]. All laboratory tests were performed at the standardized Laboratory Department (ISO 15189) of the HTD.

Patients with A(H1N1)pdm09 pneumonia were defined as those who had lung injuries confirmed by chest x-rays demonstrating parenchymal and/or interstitial injuries or alveolar injuries. Indications for ICU admission were in accordance with the national guidelines for diagnosis and treatment of influenza [30] and international recommendations [10,31] including development of respiratory distress or septic shock. These clinical admission criteria and thresholds remained consistent throughout the 10-year study period, and admission decisions were not constrained by ICU bed availability. Respiratory distress is defined as difficulties in breathing or shortness of breath, PaO_2_/FIO_2_ ≤ 200 mmHg or SpO2/FIO2 ≤ 235 (if SpO2 ≤ 97%) [32]. Septic shock is identified with a clinical construct of sepsis with persisting hypotension requiring vasopressors to maintain MAP ≥ 65 mmHg and having a serum lactate level >2 mmol/L (18 mg/dL) despite adequate volume resuscitation [33].

### Data collection

A data collection form was used to record participants’ information from HTD’s paper-based medical records. Data included demographics (age, gender, comorbidity, and pregnancy status of females), clinical signs and symptoms, chest x-ray features, laboratory test results, treatments, complications, and outcomes. Clinical features included days from onset to hospital admission, fever, fatigue, cough, dyspnea, sore throat, chest pain, and moderate-to-severe acute respiratory distress syndrome (ARDS). Based on the Berlin definition, ARDS was defined as an acute disorder that starts within seven days of the inciting event and is characterized by bilateral lung infiltrates and severe progressive hypoxemia in the absence of any evidence of cardiogenic pulmonary edema [32]. ARDS was categorized as mild, moderate, and severe [32]. Chest x-ray features included unilateral or bilateral lung infiltrates and alveolar injuries. All radiographs were interpreted by the treating clinical team and independently verified by attending staff radiologists. Laboratory characteristics included complete blood count, serum creatinine, alanine transaminase (ALT), aspartate transaminase (AST), total bilirubin, glycemia, serum albumin, C-reactive protein (CRP), procalcitonin, and arterial lactate. Baseline laboratory investigations used in the analyses were performed at hospital admission unless otherwise specified. For missing data, percentages for categorical variables were calculated based on valid cases (available data), and the actual denominator for each variable was specified where missing data occurred. Microbiological specimens were collected and investigated only when clinically suspected and indicated, rather than systematically for all patients. Treatments included antiviral therapy (standard-dose [75 mg/12h] or double-dose [150 mg/12h] oseltamivir or combination therapy [oseltamivir plus zanamivir or ribavirin]), other medications (corticosteroids and vasopressor drugs), and respiratory support (nasal cannula, mask, high-flow nasal cannula (HFNC), continuous positive airway pressure (CPAP), invasive or prone ventilation). Complications included hospital-acquired infections, which were identified based on standardized clinical and microbiological parameters, defined as any infection including ventilator-acquired pneumonia (VAP), urinary tract infection and bloodstream infection developing after two days of admission. Outcomes included mortality, hospital discharge, inter-hospital transfer together with reasons, ICU and hospital length of stay.

### Statistical analysis

Data were managed and analyzed using SPSS software version 26. Categorical variables were presented as a count and percentage and compared using the Chi-squared test, while continuous variables were presented as mean ± standard deviation (SD) and compared using Student’s t-test. For comparison purposes, 95% confidence intervals (CIs) of the point incidence of A(H1N1)pdm09-related pneumonia, ICU admission, mortality, discharge, and inter-hospital transfer were calculated. An additional comparative analysis was performed to compare ICU admission and mortality between the pandemic period (2009–2010) and the post pandemic period (2011–2019) using the χ² test or Fisher’s exact test, as appropriate. Multivariable logistic regression models were developed to test predictors of ICU admission and mortality related to A(H1N1)pdm09 pneumonia. A standard multivariable logistic regression for ICU admission prediction model was constructed. Model calibration and goodness-of-fit were formally evaluated using the Hosmer–Lemeshow test, while discrimination capacity was assessed via the Area Under the ROC Curve (AUC/ C-statistic). Multicollinearity among predictors was monitored using Variance Inflation Factors (VIF). For mortality model, due to low-event constraints (sparse mortality data), Firth’s penalized logistic regression was employed to minimize small-sample bias and avoid issues of separation. Complete-case analysis was applied to this model. Goodness-of-fit was evaluated using the Penalized Likelihood Ratio Test, and discrimination power was determined via the AUC/ C-statistic. Multicollinearity was similarly assessed using VIF. Given the potential for confounding among covariates, the purposeful selection process was used to identify covariates for the regression models. Following the methodology described elsewhere [34,35], a more generous P-value cutoff of <0.25 in the univariate analysis was used. This approach was chosen because traditional cutoffs (such as 0.05) often fail to identify variables that, while not independently significant, act as important confounders or become significant when adjusted for other covariates. Furthermore, variables considered clinically important based on the authors’ expertise were retained irrespective of their univariable statistical significance to ensure that potential confounding was appropriately addressed. Variables included in the model for predictors of ICU admission were gender; age > 60 years old; presence of any comorbidity; day from onset to admission; white blood cell (WBC), neutrophil, lymphocyte, and platelet cell counts; serum creatinine; AST; ALT; and moderate-to-severe ARDS. Variables included in the model for predictors of mortality were gender, age > 60 years old, presence of any comorbidity, day from onset to admission, WBC and lymphocyte counts, moderate-to-severe ARDS, invasive ventilation, double-dose oseltamivir or combination, corticosteroids, vasopressors, hospital-acquired infection, and ICU admission. Alpha was set at 5% level.

### Maintenance of study standard

This study was reported in line with the STROBE guideline for cohort studies (Appendix 1).

## Results

### Baseline characteristics of study participants

**Fig 1:**
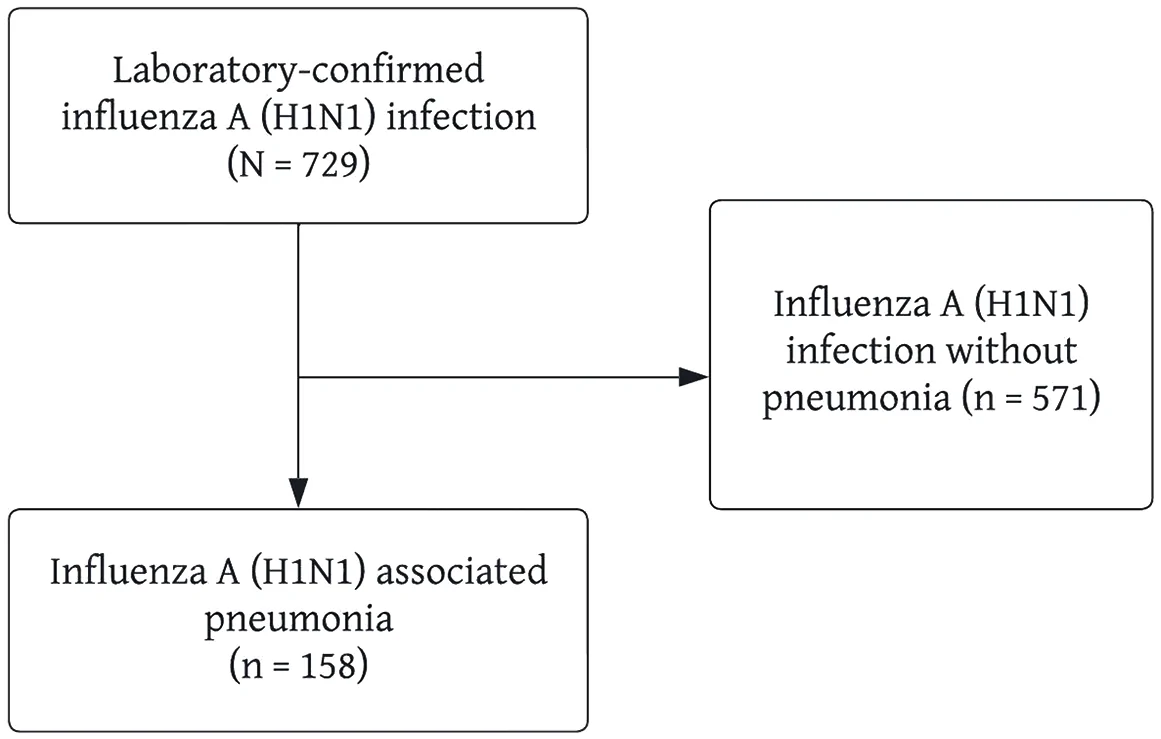
Flowchart of study participants.

**Fig 2:**
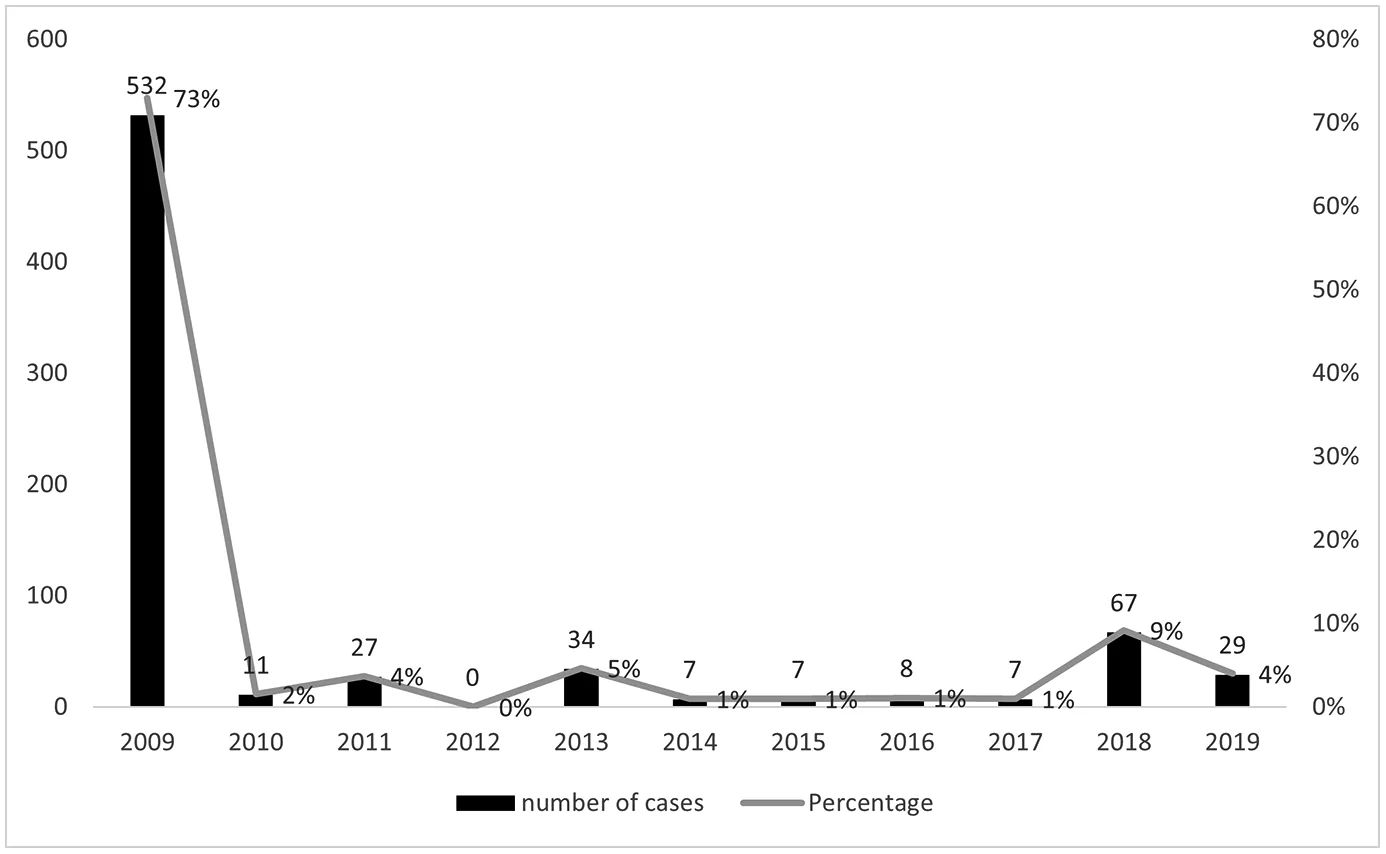
Frequency of laboratory-confirmed A(H1N1)pdm09 infected cases by year between 2009 and 2019.

A total of 729 A(H1N1)pdm09 infected patients received treatment at the HTD during the study period with no subsequent exclusions or missing data for primary outcomes (Fig 1). Most (73%, 532/729) patients were hospitalized in 2009 (Fig 2). Of these 729 patients, A(H1N1)pdm09-related pneumonia was diagnosed in 21.7% (158/729, 95% CI 18.8–24.8%).

The mean age of 158 participants with A(H1N1)pdm09-related pneumonia was 41.8 ± 16 years old, with 24 cases (15.2%) older than 60 years old. Among these 158 participants, 50.6% (80/158) were male, 16.5% (26/158) were pregnant, and 30.4% (48/158) had at least one comorbidity, including cardiovascular disease, diabetes, chronic liver disease, chronic lung disease (chronic obstructive pulmonary disease and asthma), and chronic kidney disease. The mean period from symptom onset to hospital admission was 4 ± 3 days. The most common presenting symptoms included fever (92.4%, 146/158), fatigue (61.4%, 97/158), and productive cough (51.9%, 82/158). Patients with moderate-to-severe ARDS accounted for 36.7% (58/158). Alveolar injuries (96.8%, 153/158) were the most common finding on chest x-rays (Table 1). Among the 26 pregnant patients included, 57.7% required ICU admission and three (11.5%) died. Four women went into labor while being treated for influenza, and one case resulted in stillbirth.

**Table 1:** Demographic, clinical, and chest radiographic characteristics of 158 patients with laboratory-confirmed A(H1N1)pdm09-related pneumonia.

Characteristics	Summary statistics*
Demographics	
Age (years) >60 years old	41.8 ± 16 24 (15.2)
Male	80 (50.6)
Comorbidities	48 (30.4)
Cardiovascular disease	17 (10.8)
Diabetes	14 (8.9)
Chronic liver disease	8 (5.1)
Chronic lung diseases (COPD or asthma)	6 (3.8)
Chronic kidney disease	3 (1.9)
Pregnancy	26 (16.5)
Days from disease onset to admission	4 ± 3
Clinical characteristics	
Fever	146 (92.4)
Fatigue	97 (61.4)
Productive cough	82 (51.9)
Shortness of breath	77 (48.7)
Sore throat	42 (26.6)
Chest pain	40 (25.3)
Moderate-to-severe ARDS	58 (36.7)
Chest radiographic characteristics	
Alveolar injuries	153 (96.8)
Interstitial injuries	11 (7)
Bilateral lung infiltrates	105 (66.5)
Unilateral lung infiltrates	53 (33.5)

COPD: c*hronic obstructive pulmonary* disease; ARDS: acute respiratory distress syndrome

*N (%) for categorical variables and mean ± SD for continuous variables

### Treatment and outcomes of study participants

**Fig 3:**
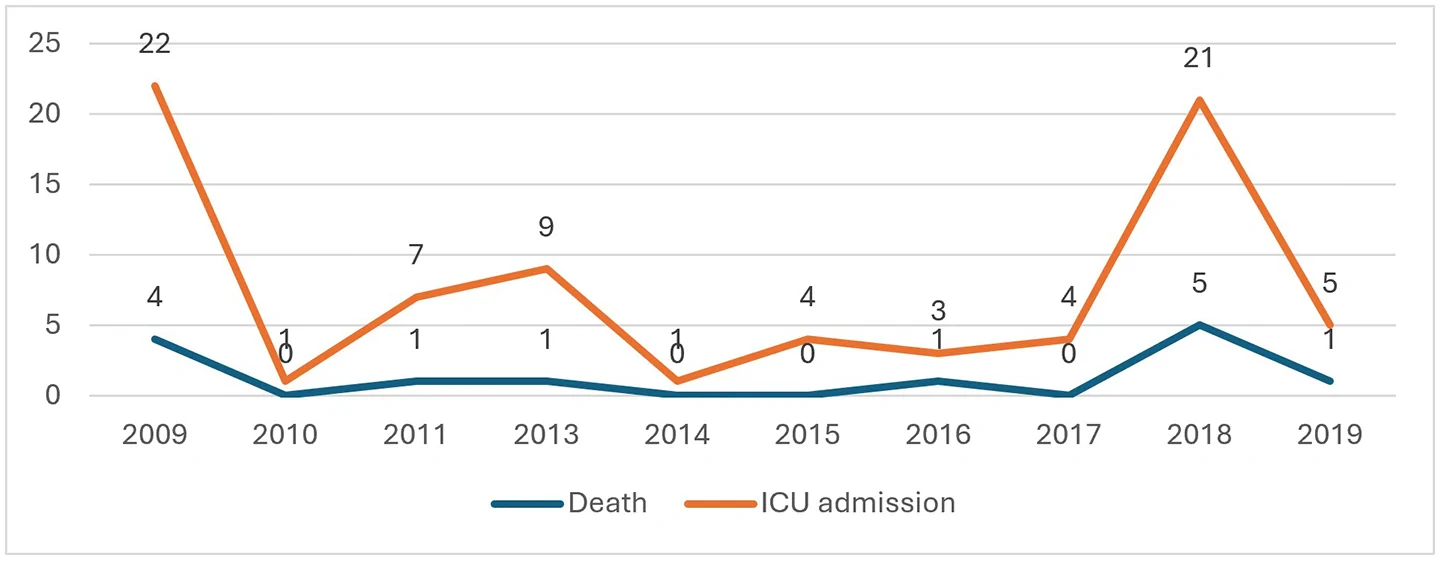
Number of ICU admitted and fatal cases during the study period.

Most patients (88%, 139/158) were treated with standard-dose oseltamivir. ICU admission accounted for 48.7% (77/158, 95%CI 41.1–56.5%). Just less than two-thirds (61.4%, 97/158) of patients received respiratory support, and invasive ventilation accounted for 15.2% (24/158). Hospital-acquired infections were documented in 6.3% (10/158) of patients. Most patients were discharged home (86.1%, 136/158, 95%CI 79.8–90.6%), and 7.6% required inter-hospital transfer (12/158, 95%CI 4.4–12.8%). Reasons for inter-hospital transfer included treatment of tuberculosis and other comorbidities, and delivery services for pregnancy. The mortality rate was 8.2% (13/158, 95%CI 4.9–13.6%). The mean hospital and ICU lengths of stay were 12.1 ± 9.1 and 6.8 ± 6.5 days, respectively (Table 2). A temporal comparison between the pandemic period (2009–2010) and the post pandemic period (2011–2019) showed that mortality did not differ significantly between the pandemic and post pandemic periods (P = 0.765). Regarding ICU admission, the percentage of patients requiring ICU admission was significantly higher in the post-pandemic period (p = 0.034) (Fig 3) (Table 3).

**Table 2:** Antiviral and supportive therapies, complications, and treatment outcomes of 158 patients with A(H1N1)pdm09-related pneumonia.

Characteristics (n = 158)	Summary statistics*
Antiviral therapy	
Standard dose oseltamivir (75 mg/12h)	139 (88)
Double dose oseltamivir (150 mg/12h) or combination^**^	19 (12.0)
ICU admission	77 (48.7)
Respiratory support*** 97 (61.4)	
Nasal cannula	37 (23.4)
Mask	24 (15.2)
HFNC or CPAP ventilation	12 (7.6)
Invasive ventilation	24 (15.2)
None	61 (38.6)
Other treatments	
Prone ventilation	12 (7.6)
Corticosteroids	25 (15.8)
Vasopressor	16 (10.1)
Hospital-acquired infections	10 (6.3)
Outcomes	
Discharge	136 (86.1)
Mortality	13 (8.2)
Inter-hospital transfer	12 (7.6)
ICU length of stay (days)	6.8 ± 6.5
Hospital length of stay (days)	12.1 ± 9.1

HFNC: High-flow nasal cannula; CPAP: continuous positive airway pressure

*N (%) for categorical variables and mean ± SD for continuous variables

**Combination therapy: oseltamivir with zanamivir (n=12) or ribavirin (n=2).

***Respiratory support categories are not mutually exclusive, with patients able to receive >1

**Table 3:** Comparison of ICU admission and mortality rate between the pandemic period (2009-2010) and the post pandemic period (2011-2019).

Rate	2009-2010	2011-2019	P^**^	OR (95%CI)
ICU admission	23 (37.7)	54 (55.7)	0.034	2.065 (1.026-4.219)
Mortality	4 (6.6)	9 (9.3)	0.765	0.687 (0.147-2.609)

** Fisher’s exact test for categorical variables)

### Unadjusted risk factors for ICU admission and mortality among 158 patients with laboratory-confirmed A(H1N1)pdm09-related pneumonia

There was a statistically significant association between ICU admission and comorbidities (OR = 1.812, 95%CI 1.348–2.435, P < 0.001), and laboratory parameters including serum creatinine (OR = 1.024, 95%CI 1.010–1.038, P < 0.001), glycemia (OR = 1.007, 95%CI 1.001–1.014, P = 0.026), AST (OR = 1.007, 95%CI 1.002–1.012, P = 0.005), and serum albumin (OR = 0.775, 95%CI 0.654–0.918, P = 0.003) (Table 4).

**Table 4:** Unadjusted risk factors tested for ICU admission among 158 patients with laboratory-confirmed A(H1N1)pdm09-related pneumonia.

Variables	ICU admission*		P value**	OR (95%Cl)
	Yes (n = 77)	No (n = 81)		
Demographic***s***				
Above 60 years old	10 (13)	14 (17.3)	0.511	0.714 (0.296–1.721)
Male	44 (57.1)	36 (44.4)	0.115	1.667 (0.888–3.127)
Comorbidities	34 (44.2)	14 (17.3)	<0.001	1.812 (1.348–2.435)
Days from disease onset to admission	5 ± 2	4 ± 3	0.128	1.115 (0.969–1.284)
Laboratory characteristics				
White blood cell (per 1000 cells/mm3)	7.994 ± 5.283	6.615 ± 3.233	0.054	1.078 (0.999–1.164)
Neutrophil (per 1000 cells/mm3)	6.357 ± 4.381	4.771 ± 3.002	0.012	1.127 (1.027–1.238)
Lymphocyte (per 1000 cells/mm3)	2.027 ± 1.149	1.189 ± 0.669	0.866	0.982 (0.794–1.214)
Platelet (per 1000 cells/mm3)	171 ± 88	195 ± 97	0.111	0.997 (0.993–1.001)
Serum creatinine (μmol/L)	86.1 ± 35.5	68.5 ± 19.8	<0.001	1.024 (1.010–1.038)
Glycemia(g/L)	131.2 ± 56.9	103.9 ± 67.8	0.026	1.007 (1.001–1.014)
AST (U/L)	109 ± 101	65 ± 65	0.005	1.007(1.002–1.012)
ALT (U/L)	67 ± 57	51 ± 48	0.396	1.002 (0.997–1.008)
Serum albumin (g/L)	29.5 ± 4.8	41.6 ± 14.1	0.003	0.775 (0.654–0.918)
Total bilirubin (μmol/L)	28.7 ± 17.7	11.1 ± 10.4	0.413	1.021 (0.972–1.073)
CRP (mg/dL)	85 ± 60	66 ± 62	0.405	1.006 (0.993–1.019)
Procalcitonin (ng/mL)	12 ± 5.6	3.3 ± 1.7	0.482	1.103(0.839–1.449)
Arterial lactate (mmol/L)	2.5 ± 2.1	2 ± 0.8	0.392	1.211 (0.781–1.876)
Chest radiographic characteristics				
Alveolar injuries***	75 (97.4)	78 (96.3)	1.000	1.442 (0.234–8.876)
Bilateral lung infiltrates****	24 (31.2)	29 (35.8)	0.503	1.299 (0.671–2.514)

*N (%) for categorical variables and mean ± SD for continuous variables

**Fisher’s exact test for categorical variables and Student’s t-test for continuous variables

***Compared with those who developed interstitial injuries

****Compared with those who developed unilateral lung infiltrates

There was a statistically significant association between mortality and moderate-to-severe ARDS (P < 0.001, OR = 23.170, 95%CI 2.905–184.789), laboratory parameters including AST (OR = 1.009, 95%CI 1.003–1.014, P < 0.001), ALT (OR = 1.007, 95%CI 1.000–1.013, P = 0.041), and serum albumin (OR = 0.774, 95%CI 0.615–0.975, P = 0.030), and treatment including the use of double-dose oseltamivir or combination (OR = 4.792, 95%CI 1.471–15.612, P = 0.009) as well as invasive ventilation (OR = 31.190, 95%CI 7.669–126.861, P < 0.001). There was also a statistically significant association between mortality and the use of corticosteroids (OR = 5.684, 95%CI 1.725–18.728, P = 0.004) and vasopressors (OR = 11.571, 95%CI 3.264–41.021, P < 0.001), hospital-acquired infections (OR = 17.500, 95%CI 4.189–73.109, P < 0.001), and ICU admission (OR=14.769, 95%CI 1.871–116.586, P = 0.011) (Table 5).

**Table 5:** Unadjusted risk factors tested for mortality among 158 patients with laboratory-confirmed A(H1N1)pdm09-related pneumonia.

Variables	Mortality*		P value**	OR (95%CI)
	Yes (n = 13)	No (n = 145)		
Demographics				
Above 60 years old	3 (23.1)	21 (14.5)	0.414	1.771 (0.450–6.975)
Male	6 (48.2)	74 (51.0)	0.736	1.216 (0.390–3.794)
Comorbidities	4 (30.8)	44 (30.3)	0.975	0.98 (0.287–3.353)
Days from disease onset to admission	6 ± 1	4 ± 3	0.129	1.112 (0.970–1.275)
Moderate-to-severe ARDS	11 (84.6)	47 (32.4)	<0.001	23.170 (2.905–184.789)
Laboratory characteristics				
White blood cell (per 1000 cells/mm3)	7.35 ± 4.73	7.28 ± 4.38	0.956	1.004 (0.883–1.141)
Neutrophil (per 1000 cells/mm^3^)	6.17 ± 4.06	5.49 ± 3.8	0.540	1.043 (0.912–1.194)
Lymphocyte (per 1000 cells/mm^3^)	0.78 ± 0.5	1.2 ± 1.54	0.121	0.355 (0.096–1.313)
Platelet (per 1000 cells/mm^3^)	202.9 ± 105.1	181.5 ± 92.0	0.431	1.002 (0.997–1.007)
Serum creatinine (µmol/L)	92 ± 35	76 ± 29	0.068	1.015 (0.999–1.031)
Glycemia (g/L)	150 ± 55	115 ± 64	0.075	1.008 (0.999–1.017)
AST (U/L)	183 ± 99	78 ± 81	< 0.001	1.009 (1.003–1.014)
ALT (U/L)	89.39 ± 119.85	48.88 ± 49.96	0.041	1.007 (1.000-1.013)
Serum albumin (g/L)	27 ± 4	36 ± 11	0.030	0.774 (0.615–0.975)
Total bilirubin (µmol/L)	11.67 ± 2.61	14.28 ± 22.91	0.843	0.993 (0.922–1.068)
CRP (mg/dL)	121 ± 57.98	71.26 ± 60.7	0.287	1.012 (0.990–1.036)
Procalcitonin (ng/mL)	12.79 ± 23.29	2. 98 ± 5.19	0.165	1.068 (0.973–1.171)
Arterial lactate (mmol/L)	3.58 ± 3.22	2.22 ± 1.58	0.056	1.299 (0.993–1.699)
Chest radiographic characteristics				
Alveolar injuries***	13 (100)	140 (96.6)	1.000	1.093 (1.041–1.147)
Bilateral lung infiltrates^****^	8 (61.5)	96 (66.2)	0.734	0.817 (0.254–2.629)
Antiviral therapy				
Double dose oseltamivir or combination of oseltamivir with zanamivir or ribavirin*****	6 (46.2)	22 (15.2)	0.009	4.792 (1.471–15.612)
Respiratory support				
Invasive ventilation******	10 (76.9)	14 (9.7)	<0.001	31.190 (7.669–126.861)
Other treatments				
Prone ventilation	12 (92.3)	134 (92.4)	0.989	0.985 (0.117–8.294)
Corticosteroids	6 (46.2)	19 (13.1)	0.004	5.684 (1.725–18.728)
Vasopressors	6 (46.2)	10 (6.9)	< 0.001	11.571 (3.264–41.021)
Hospital-acquired infections	5 (38.5)	5 (3.4)	< 0.001	17.500 (4.189–73.109)
ICU admission (n = 77)	12 (92.3)	65 (44.8)	0.011	14.769 (1.871–116.586)

*N (%) for categorical variables and mean ± SD for continuous variables.

**Fisher’s exact test for categorical variables and Student’s t-test for continuous variables.

*** Compared with those who developed interstitial injuries

****Compared with those who developed unilateral lung infiltrates

*****Compared with those who received standard dose oseltamivir.

******Compared with those who received non-invasive respiratory supports including nasal cannula, mask, high-flow nasal cannula (HFNC), and c*ontinuous positive airway pressure* (CPAP) as well as those who did not receive respiratory support

### Models for the prediction of ICU admission and mortality among 158 patients with laboratory-confirmed A(H1N1)pdm09-related pneumonia

Predictors of ICU admission included being above 60 years old (AOR 18.909, 95%CI 3.603–99.235, P < 0.001), presence of any recorded comorbidity (AOR 9.391, 95%CI 2.751–32.062, P < 0.001), WBC (AOR 1.195, 95%CI 1.037–1.378, P = 0.015), Neutrophil (AOR 11.334, 95%CI 1.856–69.22, P = 0.009), Lymphocyte (AOR 9.092, 95%CI 1.342–61.588, P = 0.024), Serum creatinine (AOR 1.032, 95%CI 1.005–1.059, P = 0.019), AST (AOR 1.015, 95%CI 1.004–1.026, P = 0.005), and ALT (AOR 0.987, 95%CI 0.974–0.999, P = 0.037) (Table 6).

**Table 6:** Logistic regression analysis of predictors of ICU admission among 158 patients with laboratory-confirmed A(H1N1)pdm09 pneumonia.

Variables	P value	AOR (95%CI)
Above 60 years old	< 0.001	18.909 (3.603–99.235)
Male	0.434	1.570 (0.507–4.860)
Comorbidities	< 0.001	9.391 (2.751–32.062)
Day from onset to admission	0.207	1.088 (0.954–1.242)
WBC (per 1000 cells/mm^3^)	0.015	1.195 (1.037–1.378)
Neutrophil (per 1000 cells/mm^3^)	0.009	11.334 (1.856–69.22)
Lymphocyte (per 1000 cells/mm^3^)	0.024	9.092 (1.342–61.588)
Platelet cell (per 1000 cells/mm^3^)	0.164	0.995 (0.988–1.002)
Serum creatinine (μmol/L)	0.019	1.032 (1.005–1.059)
AST (U/L)	0.005	1.015 (1.004–1.026)
ALT (U/L)	0.037	0.987 (0.974–0.999)

AOR: Adjusted odds ratio; ARDS: acute respiratory distress syndrome. * Model diagnostics: Hosmer–Lemeshow goodness-of-fit test: Chi-square = 3.978, df = 8, P = 0.859. The Area Under the ROC Curve (**AUC/ C-statistic**) was **0.8801**.

Predictors of mortality were invasive ventilation (AOR 8.426, 95%CI 1.105–141.366, P = 0.039) and double-dose oseltamivir or combination therapy (AOR = 8.577, 95%CI 1.416–83.341, P = 0.020) (Table 7).

**Table 7:** Firth’s penalized logistic regression analysis of predictors of mortality among 158 patients with laboratory-confirmed A(H1N1)pdm09 pneumonia.

Variables	P value	AOR (95%CI)
Above 60 years old	0.205	5.137 (0.408-98.998)
Male	0.976	1.030 (0.146-8.282)
Comorbidities	0.731	1.445 (0.181-14.109)
Days from onset to admission	0.123	1.169 (0.918-1.388)
WBC (per 1000 cells/mm^3^)	0.187	0.848 (0.631-1.068)
Lymphocyte (per 1000 cells/mm^3^)	0.647	1.121 (0.080-1.810)
Serum creatinine (μmol/L)	0.425	1.011 (0.985-1.041)
AST (U/L)	0.330	1.006 (0.994-1.017)
ALT (U/L)	0.875	0.999(0.986-1.012)
Moderate-to-severe ARDS	0.927	0.885 (0.053-12.097)
Invasive ventilation	0.039	8.426 (1.105-141.366)
Double dose oseltamivir (150 mg/12h) or combination*	0.020	8.577 (1.416-83.341)
Corticosteroids	0.286	0.371 (0.045-2.218)
Vasopressors	0.796	1.270 (0.189-7.600)
Hospital acquired infection	0.217	0.262 (0.024-2.151)
ICU admission	0.336	3.661 (0.248-81.225)

AOR: Adjusted odds ratio; ARDS: acute respiratory distress syndrome. Chi-square = 42.575, df = 16, $P < 0.001 (Penalized likelihood ratio test). Multicollinearity assessment via Variance Inflation Factors (VIF) indicated no problematic collinearity (all VIFs < 5.0, ranging from 1.087 to 3.625; highest for AST and ALT). The Area Under the ROC Curve (AUC/ C-statistic) was 0.9515.

*Combination therapy: oseltamivir with zanamivir (n=12) or ribavirin (n=2).

## Discussion

This study included all eligible patients treated at the HTD over a period of 10 years since the H1N1 pandemic. As the leading infectious disease center in southern Vietnam, HTD enabled this to be the largest and longest cohort study of its kind conducted in the country and likely in the wider tropical regions of the Asia-Pacific. Despite available treatments and vaccines, the study confirms a notable mortality in patients with A(H1N1)pdm09-related pneumonia. To reduce mortality, older patients, those with comorbidities, and those presenting with abnormal laboratory findings should be monitored closely for deterioration requiring intensive care.

During the study period, more than two-thirds inpatients infected with A(H1N1)pdm09 influenza were documented in 2009 when A(H1N1)pdm09 emerged [3]. Since then, A(H1N1)pdm09 has continued to circulate as a seasonal flu and been included in the seasonal influenza vaccines [4]. Given that influenza circulates year-round in Vietnam, both Southern and Northern hemisphere influenza vaccines together with a locally manufactured seasonal influenza vaccine are licensed [36]. Information on the uptake of seasonal influenza vaccine in Vietnam is limited. Nevertheless, a local study conducted on 750 people in the general community found that 30% of participants received a seasonal flu vaccine either in the current or preceding flu season, and 64% expressed demand for this vaccine [37]. Considering this, the reduction in the number of hospitalized A(H1N1)pdm09 cases over time may partly reflect increasing population immunity, including possible contributions from vaccination and prior exposure, although these factors could not be evaluated directly because individual vaccination status was unavailable in this study [37]. The underlying health conditions, such as cardiovascular diseases, diabetes and pregnancy, may exacerbate the severity of A(H1N1)pdm09 infection [38]. Despite this significant risk, most SEAR countries lack comprehensive seasonal influenza vaccination policies, resulting in low vaccine uptake across the region [39]. Currently, flu vaccines are not part of the country’s broader immunization efforts [37]. Considering the significant role of flu vaccination in preventing the disease, it is beneficial to add the vaccine to the Vietnam’s expanded immunization program, initially for priority populations with high risks of increased ICU admission and mortality.

Approximately 30% of our participants had at least one comorbidity, predominantly cardiovascular diseases and diabetes, and 16.5% were pregnant women. These underlying health conditions may exacerbate the severity of A(H1N1)pdm09infection [38]. Although flu-like symptoms such as fever, cough, and sore throat are not specific for early diagnosis [40], most of our participants developed these symptoms. This finding was supported by previous studies indicating that flu-like symptoms can account for up to 100% of influenza patients [41]. Alveolar injuries were the most common characteristics of lung injuries caused by A(H1N1)pdm09 among our participants, predominantly representing bilateral lung infiltrates. A Japanese study similarly found that 71% (17/24) of their influenza patients developed bilateral lung injuries [42]. However, several studies have indicated that the chest radiological findings of A(H1N1)pdm09infected patients are diverse and atypical [43], which may resemble those of other respiratory diseases, such as COVID-19 [43]. Therefore, we believe that in clinical practice, a combination of thorough history-taking, clinical examination, and laboratory results is pivotal in making an accurate diagnosis and determining appropriate treatment.

Regarding treatment, all study participants were hospitalized early and treated with oseltamivir. A meta-analysis has shown that early treatment with oseltamivir can reduce the risk of lower respiratory tract complications and antibiotic usage in adult patients [44,45]. Our participants required respiratory support at different levels, including non-invasive and invasive methods. Indeed, treatment for respiratory failure in patients with A(H1N1)pdm09 pneumonia is similar to that of pneumonia caused by other agents and is based on patients’ severity of hypoxemia and underlying conditions [30]. Despite effective treatment and vaccines, our study confirms that the mortality due to A(H1N1)pdm09-related pneumonia remains substantial. It is comparable to that of a study conducted in India (8543/114667, 7.5%, 95% CI 7.3–7.6%) during a similar time period [46] and of a study conducted in the North of the country during 2015–2017 (25/259, 9.3%, 95%CI 6.0–13.7%) [47]. In contrast, our mortality is lower than that of a study conducted in Singapore (41/172, 23.8%, 95%CI 18.1–30.7%) between June 2009 and August 2010 [48]. Direct comparisons of mortality estimates between countries should be interpreted cautiously. This is because differences in hospitalization thresholds, ICU capacity, healthcare systems, diagnostic practices, and patient population characteristics may contribute to the observed variation. Further comparative studies using standardized methodologies are needed to better understand these differences. Nevertheless, the mortality rate due to A(H1N1)pdm09 infection varies between countries, although it may be lower in developed countries, due to history of vaccination, differences in climate patterns, population density, and population age structure, as well as countries’ economic status and development [49]. In Vietnam, our mortality rate is similar to that of a study conducted in the North of the country during 2015–2017 (25/259, 9.3%, 95%CI 6.0–13.7%) [47]. In light of our findings, mortality due to A(H1N1)pdm09-related pneumonia is not rare, despite effective treatment and vaccine.

We found that age above 60 years and the presence of comorbidities were associated with ICU admission. Previous research similarly showed that elderly patients and those with comorbidities are associated with higher ICU admission rates, likely due to the increased risk of complications from A(H1N1)pdm09 infection [3,50]. Other studies also found an elevation of both AST and ALT in several A(H1N1)pdm09 infected patients, probably due to hepatic involvement in the pathogenesis of influenza [51]. We similarly observed an association between ALT, AST levels and ICU admission among our participants. elevated AST and ALT levels may reflect hepatic involvement associated with more severe systemic illness rather than representing independent predictors of mortality [51]. Furthermore, our multivariable model demonstrated that elevated WBC, neutrophil, and lymphocyte counts, along with increased serum creatinine, were independent predictors of intensive care requirement. Considering our findings, patients with A(H1N1)pdm09 infection who have at least one of the following factors: advanced age, comorbidities, liver enzyme elevation, or moderate-to-severe ARDS should be monitored closely and continuously for early identification of complications requiring intensive care. Indeed, at HTD, these characteristics have been trialed as screening criteria since the time the study was conducted. Our observations suggest that they are effective in identifying high-risk patients. Nevertheless, future studies are needed to validate the effectiveness and generalizability of these screening strategies. Regarding pregnancy, it was not identified as a predictor of disease severity or hospitalization in our study, likely due to the small number of pregnant participants. However, several complications were documented in this group. These findings highlight the substantial risks that influenza infection poses during pregnancy and underscore the importance of influenza vaccination in this high-risk group.

We found that mortality is strongly associated with invasive ventilation, which is consistent with findings from other studies [41,52]. Indeed, not all patients with influenza-related pneumonia undergo invasive ventilation, but those with more severe complications, including ARDS, are often given this type of ventilation support within 3–10 days following initial symptom onset [53]. The association likely reflects underlying disease severity among patients requiring invasive respiratory support. Thus, studies are ongoing to identify the feasible and protective ventilation protocol for patients with influenza-related pneumonia requiring mechanical ventilation [54,55]. Additionally, a study in China [56] has found that invasive ventilation is a risk factor for secondary bacterial pneumonia which can subsequently increase the risk of mortality among influenza patients. Although our findings did not demonstrate a significant relationship between hospital-acquired infections and mortality, we believe it is crucial to implement infection control and prevention strategies in critically ill patients with A(H1N1)pdm09 infection on mechanical ventilation. We also found that double-dose oseltamivir or combination therapy was associated with mortality. A recent systematic review and meta-analysis and another cohort study have shown that double-dose oseltamivir therapy does not help decrease mortality in patients with A(H1N1)pdm09 infection [57,58]. However, this review found that in the subgroups of“the multicenter group” and“the large sample size group (n ≥ 200)”, the use of double-dose oseltamivir may be associated with an increased mortality with a low degree of heterogeneity [57]. This is because most of the studies included in this review were observational and non-randomized, in which clinicians tended to prescribe higher doses of oseltamivir for severely ill patients who already had a higher risk of mortality [57]. Regarding combination therapy, a recent study found that the combination of antiviral agents with different action mechanisms shortens the duration of shedding of viable virus in hospitalized patients with severe influenza but does not induce improved clinical outcomes [59]. However, definitive evidence of the increased mortality risk associated with combination therapy is lacking. The observed association between intensified antiviral therapy and mortality most likely reflects confounding by indication. Patients with more severe disease were preferentially treated with intensified antiviral regimens rather than a direct harmful effect of the therapy itself. Current evidence does not demonstrate a clear survival benefit from intensified antiviral regimens in severe influenza, and prospective studies are needed before definitive conclusions regarding their clinical effectiveness can be drawn. Instead, priority should be given to improving early diagnosis, ensuring timely antiviral treatment, and using appropriate antimicrobials in patients with secondary bacterial infection, rather than initiating double-dose oseltamivir or combination antiviral therapy [59,60]. We also did not find any significant association between age over 60 and mortality, which has been documented in other studies [61]. Indeed, a large systematic review and meta-analysis found that the risk estimate for severe outcomes associated with influenza was heterogeneous in different countries classified by income levels [62]. Therefore, we believe that more robust studies are needed to quantify the association between age and A(H1N1)pdm09-associated mortality in Vietnam and comparable countries.

Our study has some notable limitations. Firstly, this study was based on an analysis of medical records and thus, data such as patients’ BMI [63] that may confound the study findings have not been documented. Additionally, some predictor variables contained missing data. Variables with substantial missingness were excluded from the multivariable analyses, which may have limited our ability to evaluate the effects of some potentially relevant predictors. Importantly, individual influenza vaccination status was not recorded in the historical medical records. Since vaccination can significantly reduce clinical severity, its absence may have confounded the observed associations with ICU admission, disease severity, and mortality. Secondly, although our study included all eligible patients treated during the study period, only 13 deaths occurred. Consequently, the multivariable mortality model should be interpreted cautiously, as the limited number of outcome events may have resulted in model overfitting and imprecise effect estimates, reflected by the wide confidence intervals for several adjusted odds ratios. Systematic microbiological investigations for bacterial co-infection at hospital admission were not performed for all patients. Therefore, the potential contribution of community acquired bacterial co infection to disease severity and mortality could not be evaluated in this study. In addition, although the institutional criteria for ICU admission remained unchanged throughout the study period, we cannot completely exclude temporal changes in clinical practice, supportive care, or diagnostic approaches that may have influenced patient management over the 10-year study period. Finally, because the Hospital for Tropical Diseases (HTD) serves as a specialized tertiary referral center, our cohort is inherently prone to selection bias toward more severe clinical cases. Therefore, the reported ICU admission rates and mortality estimates may be higher than those in community hospital settings and may not fully represent the broader population with general influenza infections. Nevertheless, the irregular circulation of A(H1N1)pdm09 in tropical regions and its ongoing antigenic changes underscore the need for continued surveillance. Our study provides updated evidence on the clinical features, laboratory findings, mortality patterns, and predictors of severe A(H1N1)pdm09 pneumonia in a tropical setting.

## Conclusions

Despite effective treatment and vaccines, our study confirms that the mortality due to A(H1N1)pdm09-related pneumonia remains substantial. In clinical practice, a thorough history-taking combined with clinical examination and laboratory results is essential for making an accurate diagnosis. Close and continuous monitoring of older patients and those with comorbidities, liver enzyme elevation, or moderate-to-severe ARDS is needed for a timely detection of complications that require intensive care and thus, reduce mortality. The study did not demonstrate improved outcomes with intensified antiviral regimens. However, prospective studies are required before definitive treatment recommendations can be made. Further robust, large-scale studies are warranted to more comprehensively profile mortality risk factors associated with A(H1N1)pdm09 pneumonia in Vietnam and similar Asian populations.

## Abbreviations

ALT: alanine transaminase
ARDS: acute respiratory distress syndrome
AST: aspartate transaminase
CIs: confidence intervals
CPAP: continuous positive airway pressure
CRP: C-reactive protein
HFNC: high-flow nasal cannula
HTD: Hospital for Tropical Diseases
ICU: intensive care unit
RT-PCR: reverse transcription polymerase chain reaction
SD: standard deviation
VAP: ventilator-acquired pneumonia
WBC: white blood cell
WHO: World Health Organization
