# Supplementary material for: ICU admission and mortality in adult patients with influenza A(H1N1)pdm09-related pneumonia in Vietnam since the 2009 H1N1 pandemic: A 10-year cohort study
Source: PLoS One. 2026 Aug 24;21(8):e0348450. doi: 10.1371/journal.pone.0348450 (PMC13502601; doi:10.1371/journal.pone.0348450)
Supplement: Supplementary file 1 [file pone.0348450.s004.pdf]

Dear Editor and Reviewers,

Thank you very much for taking the time to review our manuscript and for your valuable feedback, which we greatly appreciate. Please find our detailed responses to your comments below.

Handling Editor's comments:

1. **Given that influenza circulates year-round in Vietnam, both Southern and Northern hemisphere influenza vaccines together with a locally manufactured seasonal influenza vaccine are licensed. The authors should introduce the seasonality of influenza A in Southern and Northern hemisphere, as well as epidemiology of influenza and co-infection with other respiratory viruses in the post-COVID era. More references should be cited, with this one (PMID: 40137747) as an example (citing is optional).**

Response: We thank the Editor for this valuable suggestion. We agree that additional background on influenza seasonality, post-COVID epidemiology, and viral co-infection strengthens the rationale for our study. Accordingly, we have revised the Introduction by incorporating recent evidence on global influenza circulation, changes in influenza epidemiology following the COVID 19 pandemic, and the clinical relevance of influenza and SARS-CoV-2 co-infection. The revised text is shown below.

Introduction – 1st paragraph

While influenza seasonality is well defined in temperate regions, circulation patterns in tropical areas are often irregular, which may compromise the optimal timing and impact of vaccination programs [5]. Despite the availability of effective vaccines, A(H1N1)pdm09 remains a significant contributor to worldwide influenza-related morbidity and mortality [6]. From October 2024 through May 2025 influenza activity was reported in all influenza transmission zones [7]. The predominant viruses varied across transmission zones and between countries [7]. A study conducted in China found that between 2010 and 2015, the influenza-associated mortality was 9.9 per 100,000 people, in which influenza A(H3N2) virus was the leading cause with a rate of 5.18 per 100,000, followed by A(H1N1)pdm09 with a rate of 2.9 per 100,000[8]. Additionally, the prevalence of influenza shifted between the Southern and Northern Hemispheres following

the COVID-19 pandemic. Specifically, the post-pandemic peak of influenza in the Northern Hemisphere occurred as early as December, with the epidemic's duration increasing to 11 weeks. Meanwhile, in the Southern Hemisphere, the epidemic increased in length to 20 weeks [9]. Given that influenza A/B and SARS-CoV-2 share similar transmission pathways (via aerosolized droplets and contaminated surfaces) and clinical presentations, and typically circulate in overlapping seasonal outbreaks, their potential for co-infection poses a significant public health threat [9].

2. **Lines 129-137 "Nasal and oropharyngeal swabs were collected ... All laboratory tests were performed at the standardized Laboratory Department (ISO 15189) of the HTD." The methods section describes influenza A and influenza B detection using PCR, but it does not provide influenza A subtype (typing) information. In addition, the authors should introduce that LAMP has been successfully used for the detection of influenza A virus. More references should be cited, with this one (Liu, B.M. Isothermal nucleic acid amplification technologies and CRISPR-Cas based nucleic acid detection strategies for infectious disease diagnostics. p 30-47. In Manual of Molecular Microbiology: Fundamentals and Applications; ASM Press: Washington, DC, USA, 2025.doi:10.1128/9781683674597.ch03.) as an example (citing is optional).**

Response: We thank the Editor for this helpful suggestion. We have revised the Materials and Methods section to clarify the influenza A subtyping procedure used in this study. We have also briefly acknowledged Loop Mediated Isothermal Amplification (LAMP) as an emerging molecular diagnostic method for influenza A, while noting that real-time RT PCR remained the routine diagnostic platform throughout the study period. The revised text is shown below.

Materials and methods - A(H1N1)pdm09 laboratory confirmation, definitions of A(H1N1)pdm09 pneumonia, and indications for ICU admission paragraph

Nasal and oropharyngeal swabs were collected by medical staff as per the HTD's guidelines. Loop Mediated Isothermal Amplification (LAMP) has also been reported as a rapid molecular method for detecting influenza A virus [25]. However, throughout the study period, real-time RT PCR remained the routine diagnostic method at our institution and was therefore used for patient enrolment. RT-PCR was performed on the LightCycler 480 II System (Roche Molecular Diagnostics, Pleasanton, CA) [26, 27] using the QIAGEN OneStep RT-PCR kit [27, 28].

Reviewer 1's comments:

1. **Overall Evaluation**

This manuscript presents a 10-year retrospective cohort study evaluating ICU admission, mortality, and predictors of severe outcomes among adults with A(H1N1)pdm09 pneumonia in Vietnam. The study addresses an important public health issue, particularly in tropical and resource-limited settings where long-term influenza surveillance data are limited. The long study duration and inclusion of real-world clinical data are notable strengths.

The manuscript is clinically relevant and provides useful epidemiological and outcome data from a tropical Asian setting. However, several methodological, statistical, and presentation-related issues should be addressed before publication. Some conclusions are currently overstated relative to the observational nature of the study and the limited sample size, especially regarding antiviral therapy and mortality.

Overall recommendation: Major Revision

Response: We sincerely appreciate your evaluation and constructive feedback. We are grateful for the recognition of our study's clinical relevance and its contribution to filling surveillance gaps in tropical settings.

We have taken your concerns very seriously and have comprehensively addressed the methodological, statistical, and presentation-related issues in this revised manuscript. All individual queries have been answered in detail below.

2. **Clarify Study Objective and Primary Outcomes**
   The objectives should be more explicitly stated in the Introduction. Currently, the manuscript discusses ICU admission, mortality, and treatment outcomes, but the primary and secondary outcomes are not clearly defined.
   Suggested improvement:
   At the end of the Introduction, clearly state:
   - **Primary outcomes: ICU admission and mortality**
   - **Secondary outcomes: ventilation requirement, ARDS, hospital-acquired infection, and length of stay**

Response: We thank the Reviewer for this excellent suggestion. While these endpoints were initially detailed in the Materials and Methods section, we agree that explicitly defining them at the end of the Introduction enhances the clarity of our study objectives. We have revised the final paragraph of the Introduction as follows:

Introduction – 3rd paragraph

To strengthen influenza prevention and control in Vietnam and similar settings, continual assessment of disease severity is needed [22]. This study aimed to examine the clinical burden and identify predictors of severe disease among adult patients with A(H1N1)pdm09-related pneumonia. The primary outcomes were ICU admission and all-cause mortality. The secondary outcomes included respiratory support requirements (such as invasive ventilation), development of moderate-to-severe ARDS, occurrence of hospital-acquired infections, and overall hospital and ICU lengths of stay.

3. **Concern Regarding Small Number of Mortality Events**

The mortality analysis appears statistically underpowered. Only 13 deaths occurred, yet many variables were entered into the multivariable logistic regression model. This raises concern for:

- **Model overfitting**
- **Unstable estimates**
- **Extremely wide confidence intervals**
- **Reduced reliability of adjusted odds ratios**

Examples:

- **Invasive ventilation AOR 55.355 (95% CI 1.486–2062.375)**
- **Double-dose oseltamivir/combination therapy AOR 32.625 (95% CI 1.594–667.661)**

These wide confidence intervals indicate substantial statistical instability.

Suggestions:

- **Reduce the number of variables included in the mortality regression model.**
- **Consider using penalized logistic regression or Firth logistic regression.**
- **Include an events-per-variable justification.**
- **Acknowledge possible model instability more explicitly in limitations.**

Response: We thank the Reviewer for raising this important statistical concern. We acknowledge that the small number of deaths (n = 13) limits the precision of the mortality model and increases the risk of model overfitting. Variable selection followed a pre-defined purposeful selection strategy based on both statistical criteria ($P < 0.25$ in the univariable analysis) and clinical relevance, as recommended in the methodological literature. We retained clinically important variables to reduce potential confounding, but we also recognised

that the limited number of events resulted in wide confidence intervals for several estimates. Based on your suggestion, we used Firth logistic regression to strengthen the power of mortality. Additionally, we have included model diagnostics appropriate for each multivariable model. For the ICU admission model, calibration was assessed using the Hosmer–Lemeshow goodness-of-fit test, and discrimination was evaluated using the area under the receiver operating characteristic curve (AUC). For the mortality model, which was analysed using Firth's penalized logistic regression, model performance was evaluated using the penalized likelihood ratio test together with the AUC. Multicollinearity was assessed using variance inflation factors (VIFs), and no evidence of problematic multicollinearity was identified among the variables included in the mortality model. Despite these strategies, we acknowledge that the number of covariates remains high relative to the number of events and therefore regard the mortality model as exploratory. In response to this, we further strengthened the Limitations section to explicitly acknowledge the exploratory nature of the mortality model and the possibility of model instability. The revised text is shown below:

Materials and methods – statistical analysis paragraph

"…A standard multivariable logistic regression for ICU admission prediction model was constructed. Model calibration and goodness-of-fit were formally evaluated using the Hosmer–Lemeshow test, while discrimination capacity was assessed via the Area Under the ROC Curve (AUC / C-statistic). Multicollinearity among predictors was monitored using Variance Inflation Factors (VIF). For mortality model, due to low-event constraints (sparse mortality data), Firth's penalized logistic regression was employed to minimize small-sample bias and avoid issues of separation. Complete-case analysis was applied to this model. Goodness-of-fit was evaluated using the Penalized Likelihood Ratio Test, and discrimination power was determined via the AUC / C-statistic. Multicollinearity was similarly assessed using VIF. Given the potential for confounding among covariates, the purposeful selection process was used to identify covariates for the regression models. Following the methodology described elsewhere [34, 35], a more generous P-value cutoff of <0.25 in the univariate analysis was used. This approach was chosen because traditional cutoffs (such as 0.05) often fail to identify variables that, while not independently significant, act as important confounders or become significant when adjusted for other covariates. Furthermore, variables considered clinically important based on the authors' expertise were retained irrespective of their univariable statistical significance to ensure that potential confounding was appropriately addressed."

Results

Table 7. Firth's penalized logistic regression analysis of predictors of mortality among 158 patients with laboratory-confirmed A(H1N1)pdm09 pneumonia

Variables	P value	AOR (95%CI)
Above 60 years old	0.205	5.137 (0.408-98.998)
Male	0.976	1.030 (0.146-8.282)
Comorbidities	0.731	1.445 (0.181-14.109)
Days from onset to admission	0.123	1.169 (0.918-1.388)
WBC (per 1000 cells/mm^3)	0.187	0.848 (0.631-1.068)
Lymphocyte (per 1000 cells/mm^3)	0.647	1.121 (0.080-1.810)
Serum creatinine (μmol/L)	0.425	1.011 (0.985-1.041)
AST (U/L)	0.330	1.006 (0.994-1.017)
ALT (U/L)	0.875	0.999(0.986-1.012)
Moderate-to-severe ARDS	0.927	0.885 (0.053-12.097)
Invasive ventilation	0.039	8.426 (1.105-141.366)
Double dose oseltamivir (150mg/12h) or combination*	0.020	8.577 (1.416-83.341)
Corticosteroids	0.286	0.371 (0.045-2.218)
Vasopressors	0.796	1.270 (0.189-7.600)
Hospital acquired infection	0.217	0.262 (0.024-2.151)
ICU admission	0.336	3.661 (0.248-81.225)

AOR: Adjusted odds ratio; ARDS: acute respiratory distress syndrome. Chi-square = 42.575, df = 16, $P < 0.001 (Penalized likelihood ratio test). Multicollinearity assessment via Variance Inflation Factors (VIF) indicated no problematic collinearity (all VIFs < 5.0, ranging from 1.087 to 3.625; highest for AST and ALT). The Area Under the ROC Curve (AUC / C-statistic) was 0.9515.

*Combination therapy: oseltamivir with zanamivir (n=12) or ribavirin (n=2).

To ensure complete transparency and help readers better interpret our findings, we have expanded the Limitations section to explicitly address how the small number of events affects model precision and stability:

Discussion – Limitations paragraph

“Our study has some notable limitations. Firstly, this study was based on an analysis of medical records and thus, data such as patients’ BMI [63] that may confound the study findings have not been documented. Additionally, some predictor variables contained missing data. Variables with substantial missingness were excluded from the multivariable analyses, which may have limited our ability to evaluate the effects of some potentially relevant predictors. Importantly, individual influenza vaccination status was not recorded in the historical medical records. Since vaccination can significantly reduce clinical severity, its absence may have confounded the observed associations with ICU admission, disease severity, and mortality. Secondly, although our study included all eligible patients treated during the study period, only 13 deaths occurred. Consequently, the multivariable mortality model should be interpreted cautiously, as the limited number of outcome events may have resulted in model overfitting and imprecise effect estimates, reflected by the wide confidence intervals for several adjusted odds ratios. Systematic microbiological investigations for bacterial co-infection at hospital admission were not performed for all patients. Therefore, the potential contribution of community acquired bacterial co-infection to disease severity and mortality could not be evaluated in this study. In addition, although the institutional criteria for ICU admission remained unchanged throughout the study period, we cannot completely exclude temporal changes in clinical practice, supportive care, or diagnostic approaches that may have influenced patient management over the 10-year study period. Finally, because the Hospital for Tropical Diseases (HTD) serves as a specialized tertiary referral center, our cohort is inherently prone to selection bias toward more severe clinical cases. Therefore, the reported ICU admission rates and mortality estimates may be higher than those in community hospital settings and may not fully represent the broader population with general influenza infections…”

4. Interpretation of Antiviral Therapy Findings is Overstated

The manuscript repeatedly implies that double-dose oseltamivir or combination therapy may increase mortality. However, this conclusion is not sufficiently supported because:

- **This is an observational retrospective study.**
- **Sicker patients are more likely to receive aggressive therapy (confounding by indication).**
- **Disease severity itself likely influenced treatment decisions.**

Therefore, the association should not be interpreted causally.

Current problematic statements:

- **"Neither double-dose oseltamivir nor combination therapy is recommended…"**
- **"Alternative antiviral regimens should not be recommended."**

These conclusions exceed the evidence generated by this study.

Suggested revision:

Replace with more cautious wording such as:

"The observed association between intensified antiviral therapy and mortality likely reflects treatment allocation to more severely ill patients rather than a direct harmful effect of therapy."

And:

"Further prospective studies are required to evaluate the efficacy of intensified antiviral regimens in severe influenza."

Response:

We thank the Reviewer for this important comment. We agree that the observed association should not be interpreted as causal because this retrospective observational study is susceptible to confounding by indication. Therefore, we have revised the Discussion and Conclusion to avoid causal language and to emphasise that patients receiving intensified antiviral therapy were likely those with more severe disease. The revised text is shown below.

Discussion – 6th paragraph

"…The observed association between intensified antiviral therapy and mortality most likely reflects confounding by indication. Patients with more severe disease were preferentially treated with intensified antiviral regimens rather than a direct harmful effect of the therapy itself. Current evidence does not demonstrate a clear survival benefit from intensified antiviral regimens in severe influenza, and prospective studies are needed before definitive conclusions regarding their clinical effectiveness can be drawn…"

5. **ICU Admission Criteria Need Better Standardization**

The manuscript states that ICU admission followed national and international guidelines. However, ICU admission practices can vary over time, physicians, and hospital capacity.

Given the 10-year study duration, ICU admission thresholds may have changed.

Suggestions:

- **Clarify whether ICU criteria remained consistent throughout the study period.**
- **Mention whether ICU bed availability influenced admission decisions.**
- **Discuss possible temporal bias.**

Response:

We thank the Reviewer for this important comment. We have clarified the manuscript to state that the institutional criteria for ICU admission remained consistent throughout the study period and were based on national and international guidance. We have also clarified that no institutional policy restricted ICU admission because of ICU bed availability during the study period. Although temporal changes in clinical practice cannot be completely excluded in a retrospective study, the consistency of ICU admission criteria has now been explicitly described in the Methods and acknowledged in the Limitations.

Methods section – A(H1N1)pdm09 laboratory confirmation, definitions of A(H1N1)pdm09 pneumonia, and indications for ICU admission section – the 2nd paragraph

"…Indications for ICU admission were in accordance with the national guidelines for diagnosis and treatment of influenza [30] and international recommendations [10, 31] including development of respiratory distress or septic shock. These clinical admission criteria and thresholds remained consistent throughout the 10-year study period, and admission decisions were not constrained by ICU bed availability."

Discussion section – the Limitations paragraph:

"In addition, although the institutional criteria for ICU admission remained unchanged throughout the study period, we cannot completely exclude temporal changes in clinical practice, supportive care, or diagnostic approaches that may have influenced patient management over the 10-year study period."

6. **Temporal Changes Across 2009–2019 Were Not Analyzed**

More than 70% of cases occurred in 2009, yet the study combines all years together.

Clinical management, vaccination uptake, antiviral use, and ICU care likely evolved over time.

Suggestions:

- **Include subgroup analysis comparing:**
  - **Pandemic period (2009–2010)**
  - **Post-pandemic years**
- **Assess whether mortality or ICU admission differed by time period.**
- **Alternatively, discuss this limitation more thoroughly.**

Response:

We thank the Reviewer for this valuable suggestion. We agree that evaluating potential temporal differences between the pandemic and post pandemic periods strengthens the interpretation of our findings. To address this comment, we performed an additional comparative analysis by dividing the cohort into the pandemic period (2009–2010) and the post pandemic period (2011–2019). We compared the proportions of ICU admission and mortality between the two periods. The analysis showed that mortality did not differ significantly between the pandemic and post pandemic periods ($P = 0.765$). Regarding ICU admission, the percentage of patients requiring ICU admission was significantly higher in the post-pandemic period ($p = 0.034$). This difference may reflect temporal changes in ICU availability rather than the severity of the disease. We have explained our statistical approach in the Methods section and incorporated these findings into the Results section and added a new figure illustrating the annual distribution of hospitalized cases, ICU admissions, and deaths throughout the study period (Figure 3). These additional analyses suggest that, despite substantial variation in the number of hospitalized cases across years, there was no evidence of a temporal difference in the proportions of patients experiencing in hospital mortality over the study period.

Materials and Methods section – statistical analysis paragraph:

"…An additional comparative analysis was performed to compare ICU admission and mortality between the pandemic period (2009–2010) and the post pandemic period (2011–2019) using the χ^2 test or Fisher's exact test, as appropriate."

Results section – the first paragraph:

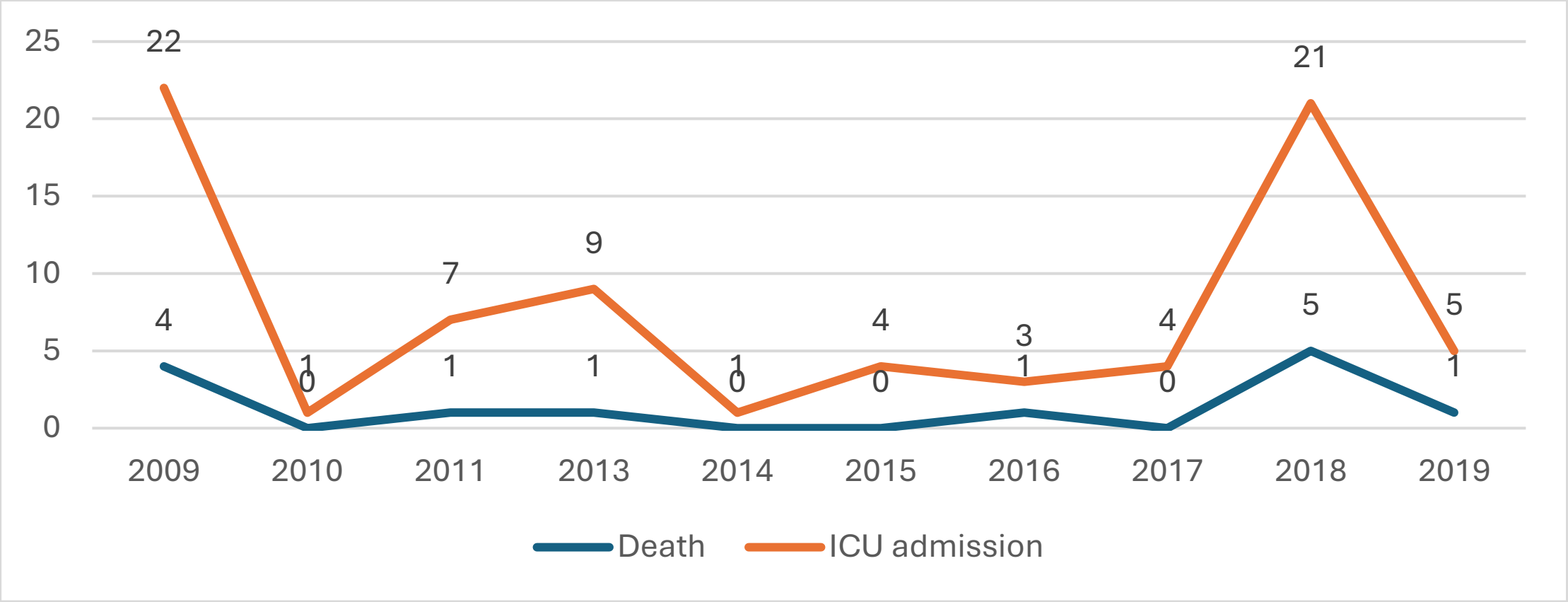

Figure 3. Number of ICU admitted and fatal cases during the study period

"…Reasons for inter-hospital transfer included treatment of tuberculosis and other comorbidities, and delivery services for pregnancy (data not shown). The mortality rate was 8.2% (13/158, 95%CI 4.9–13.6%). The mean hospital and ICU lengths of stay were 12.1 ± 9.1 and 6.8 ± 6.5 days, respectively (Table 2). A temporal comparison between the pandemic period (2009–2010) and the post pandemic period (2011–2019) showed that mortality did not differ significantly between the pandemic and post pandemic periods (P = 0.765). Regarding ICU admission, the percentage of patients requiring ICU admission was significantly higher in the post-pandemic period (p = 0.034). Figure 3 illustrates the annual distribution of hospitalized cases, ICU admissions, and deaths during the study period."

Table 1. Comparison of ICU admission and mortality rate between the pandemic period (2009-2010) and the post pandemic period (2011-2019)

	**2009-2010**	**2011-2019**	**P***	**OR (95%CI)**
ICU admission	23 (37.7)	54 (55.7)	0.034	2.065 (1.026-4.219)
Mortality	4 (6.6)	9 (9.3)	0.765	0.687 (0.147-2.609)

(**Fisher's exact test for categorical variables)

7. **ARDS and ICU Admission Relationship May Introduce Circular Reasoning**

Moderate-to-severe ARDS is identified as a predictor of ICU admission, but ARDS itself is often one of the indications for ICU transfer.

Thus, the relationship may partly reflect clinical decision-making rather than independent prediction.

Suggestions:

- **Clarify timing of ARDS diagnosis relative to ICU admission.**

- **Discuss possible incorporation bias.**

Response:

We thank the Reviewer for this important methodological observation. We agree that moderate-to-severe ARDS is closely related to the clinical decision to admit patients to the ICU. Hence, including it as a predictor of ICU admission may introduce incorporation bias. In this retrospective study, ARDS status was extracted from the complete hospitalization record and was not consistently documented before ICU admission. Therefore, the temporal relationship between ARDS onset and ICU admission could not be established with certainty. Based on your kind inputs, we removed moderate-to-severe ARDS from the multivariable model evaluating predictors of ICU admission to avoid incorporation bias. Table 5 and the corresponding Results and Discussion have been revised accordingly as follows.

Result section:

Table 6. Logistic regression analysis of predictors of ICU admission among 158 patients with laboratory-confirmed A/(H1N1)pdm09pneumonia

Variables	P value	AOR (95%CI)
Above 60 years old	< 0.001	18.909 (3.603–99.235)
Male	0.434	1.57 (0.507–4.86)
Comorbidities	< 0.001	9.391 (2.751–32.062)
Day from onset to admission	0.207	1.088 (0.954–1.242)
WBC (per mm^3)	0.015	1.195 (1.037–1.378)
Neutrophil (per mm^3)	0.009	11.334 (1.856–69.22)
Lymphocyte (per mm^3)	0.024	9.092 (1.342–61.588)
Platelet cell (per mm^3)	0.164	0.995 (0.988–1.002)
Serum creatinine (µmol/L)	0.019	1.032 (1.005–1.059)
AST (U/L)	0.005	1.015 (1.004–1.026)
ALT (U/L)	0.037	0.987 (0.974–0.999)

AOR: Adjusted odds ratio; ARDS: acute respiratory distress syndrome. * Model diagnostics: Hosmer–Lemeshow goodness-of-fit test: Chi-square = 3.978, df = 8, P = 0.859.

Predictors of ICU admission included being above 60 years old (AOR 18.909, 95%CI 3.603–99.235, P<0.001), presence of any recorded comorbidity (AOR 9.391, 95%CI 2.751–32.062, P<0.001), WBC (AOR 1.195, 95%CI 1.037–1.378, P=0.015), Neutrophil (AOR 11.334, 95%CI 1.856–69.22, P=0.009), Lymphocyte (AOR 9.092, 95%CI 1.342–61.588, P=0.024), Serum creatinine (AOR 1.032, 95%CI 1.005–1.059, P=0.019), AST (AOR 1.015, 95%CI 1.004–1.026, P=0.005), and ALT (AOR 0.987, 95%CI 0.974–0.999, P=0.037) (Table 6).

Discussion – 5th paragraph

"…We similarly observed an association between ALT, AST levels and ICU admission among our participants. Furthermore, our multivariable model demonstrated that elevated WBC,

neutrophil, and lymphocyte counts, along with increased serum creatinine, were independent predictors of intensive care requirement. Considering these findings, patients with A(H1N1)pdm09 infection who present with advanced age, baseline comorbidities, liver enzyme elevation, acute kidney parameters, or marked leukocytosis and neutrophilia should be monitored closely and continuously for timely detection of complications and the need for ICU admission…"

8. **Lack of Vaccination Status is a Significant Limitation**

Influenza vaccination status is highly relevant when studying severity and mortality.

The absence of vaccination data should be highlighted more strongly because it may substantially confound:

- **ICU admission**
- **Disease severity**
- **Mortality**

Suggestion:

Explicitly discuss this as a major limitation.

Response:

We thank the Reviewer for this important observation. We agree that individual influenza vaccination status is an important potential confounder when evaluating disease severity, ICU admission, and mortality. Unfortunately, because this was a retrospective study based on historical paper medical records, vaccination status was not routinely documented. Therefore, it could not be evaluated. We have now acknowledged this explicitly as a major limitation of the study as follows.

Discussion – 7th paragraph

"…Additionally, some predictor variables contained missing data. Variables with substantial missingness were excluded from the multivariable analyses, which may have limited our ability to evaluate the effects of some potentially relevant predictors. Importantly, individual influenza vaccination status was not recorded in the historical medical records. Since vaccination can significantly reduce clinical severity, its absence may have confounded the observed associations with ICU admission, disease severity, and mortality…"

9. **Potential Selection Bias**

The Hospital for Tropical Diseases is a referral center, which likely receives more severe cases.

Therefore:

- **Mortality estimates may not represent the general population.**
- **ICU admission rates may be higher than in community hospitals.**

Suggestion: State this limitation clearly in Discussion.

Response: We completely agree with the Reviewer's comment. As the leading tertiary referral center for infectious diseases in southern Vietnam, our institution naturally admits a higher proportion of critically ill patients transferred from primary and community hospitals. Consequently, our observed ICU admission and mortality rates may be higher than those seen in the general population or non-referral settings.

We have explicitly addressed this potential selection bias in the Limitations section to ensure accurate interpretation of our findings:

Discussion – Limitations paragraph

"…In addition, although the institutional criteria for ICU admission remained unchanged throughout the study period, we cannot completely exclude temporal changes in clinical practice, supportive care, or diagnostic approaches that may have influenced patient management over the 10-year study period. Finally, because the Hospital for Tropical Diseases (HTD) serves as a specialized tertiary referral center, our cohort is inherently prone to selection bias toward more severe clinical cases. Therefore, the reported ICU admission rates and mortality estimates may be higher than those in community hospital settings and may not fully represent the broader population with general influenza infections….

10. **Clarify Definitions of Pneumonia and Secondary Infection**

The manuscript defines pneumonia using chest radiography findings, but additional clarification is needed.

Suggestions:

Clarify:

- **Whether radiographs were independently reviewed**
- **Whether bacterial co-infection at admission was assessed**
- **Diagnostic criteria for hospital-acquired infection**

- **Whether microbiological confirmation was available**

Response: We thank the Reviewer for these important comments. We have revised the Methods section to clarify four aspects of our study. First, we have specified that chest radiographs were interpreted by the treating clinicians and independently verified by attending radiologists. Second, we have clarified that microbiological investigations for bacterial or fungal co infection were performed when clinically indicated at admission or during hospitalization. Third, we have expanded the definition of hospital acquired infection according to the institutional diagnostic criteria used in our hospital. Finally, we have clarified that microbiological confirmation was available for only a small number of patients with clinically suspected secondary infection, and therefore these findings were not analysed separately because of the limited sample size. Instead, our study recorded hospital acquired infections that developed during hospitalization according to predefined clinical and microbiological criteria. We have clarified this distinction in the Methods section and acknowledged it as a limitation of the study.

1. Radiograph review: Chest X-rays were initially evaluated by the attending clinical team and independently verified by hospital radiologists to ensure diagnostic consistency. We have clarified this in the text.
2. Bacterial co-infection at admission: Standard bacterial cultures (including blood, sputum, or bronchoalveolar lavage [BAL]) were routinely collected at admission whenever a secondary bacterial infection or co-infection was clinically suspected.
3. Diagnostic criteria for hospital-acquired infection (HAI): We have explicitly added the clinical and microbiological criteria used to define secondary complications into the Data Collection subsection.
4. Microbiological confirmation details: Baseline or secondary microbiological cultures confirmed bacterial or fungal infections in exactly 10 cases. These included Acinetobacter baumannii (n=3), Pseudomonas aeruginosa (n=2), Aspergillus fumigatus (n=2), Klebsiella pneumoniae (n=1), Staphylococcus aureus (n=1), and Stenotrophomonas maltophilia (n=1). Given that the number of microbiologically confirmed infections was limited, these findings were not analysed separately but were incorporated into the broader category of hospital acquired infection.

We have addressed each point below:

Materials and Methods – Data collection paragraph

"… A data collection form was used to record participants' information from HTD's paper-based medical records. Data included demographics (age, gender, comorbidity, and pregnancy status of females), clinical signs and symptoms, chest x-rays features, laboratory test results,

treatments, complications, and outcomes. Clinical features included days from onset to hospital admission, fever, fatigue, cough, dyspnea, sore throat, chest pain, and moderate-to-severe acute respiratory distress syndrome (ARDS). Based on the Berlin definition, ARDS was defined as an acute disorder that starts within seven days of the inciting event and is characterized by bilateral lung infiltrates and severe progressive hypoxemia in the absence of any evidence of cardiogenic pulmonary edema [16]. ARDS was categorized as mild, moderate, and severe [16]. Chest x-ray features included unilateral or bilateral lung infiltrates and alveolar injuries. All radiographs were interpreted by the treating clinical team and independently verified by attending staff radiologists. Laboratory characteristics included complete blood count, serum creatinine, alanine transaminase (ALT), aspartate transaminase (AST), total bilirubin, glycemia, serum albumin, C-reactive protein (CRP), procalcitonin, and arterial lactate. Treatments included antiviral therapy (standard-dose [75mg/12h] or double-dose [150mg/12h] oseltamivir or combination therapy [oseltamivir plus zanamivir or ribavirin]), other medications (corticosteroids and vasopressor drugs), and respiratory support (nasal cannula, mask, high-flow nasal cannula (HFNC), continuous positive airway pressure (CPAP), invasive or prone ventilation). Complications included hospital-acquired infections, which were identified based on standardized clinical and microbiological parameters, defined as any infection including ventilator-acquired pneumonia (VAP), urinary tract infection and bloodstream infection developing after two days of admission. Outcomes included mortality, hospital discharge, inter-hospital transfer together with reasons, ICU and hospital length of stay…"

Discussion section – limitations paragraph

"…Systematic microbiological investigations for bacterial co-infection at hospital admission were not performed for all patients. Therefore, the potential contribution of community acquired bacterial co infection to disease severity and mortality could not be evaluated in this study."

11. Statistical Presentation Needs Improvement

Several odds ratios are reported as 1.000 (1.000–1.000), which is not informative.

Suggestions:

- **Rescale laboratory variables (e.g., per 1000 cells/mm3 or per 10-unit increase).**
- **Present clinically meaningful increments.**
- **Provide model diagnostics:**

- **Hosmer–Lemeshow test**
- **Multicollinearity assessment**
- **ROC curve/AUC if available**

Response: We sincerely thank the Reviewer for this vital statistical guidance. We recognize that reporting unadjusted and adjusted odds ratios as 1.000 (1.000–1.000) for white blood cells, neutrophils, and lymphocytes was uninformative due to automatic software rounding for single-cell increments. As suggested, we have rescaled these continuous laboratory variables to reflect clinically meaningful increments of 1,000 cells/mm^3 (by dividing the raw values by 1,000) and re-ran our analyses. This has successfully resolved the rounding artifact and yielded informative metrics. We have updated Table 3, Table 4, Table 5, and Table 6 with these rescaled values, and completely updated the corresponding statistical data in the Abstract, Results, and Discussion sections. Additionally, we have included model diagnostics appropriate for each multivariable model. For the ICU admission model, calibration was assessed using the Hosmer–Lemeshow goodness-of-fit test, whereas the mortality model, which used Firth's penalized logistic regression, was evaluated using the penalized likelihood ratio test. Discrimination was assessed using the area under the receiver operating characteristic curve (AUC), and multicollinearity was assessed using variance inflation factors (VIFs).

Predictors of ICU Admission Model:

The Hosmer–Lemeshow goodness-of-fit test indicated no significant difference between observed and predicted outcomes (chi-square= 3.978$, df = 8, p = 0.859), suggesting no evidence of poor model fit. Discrimination capacity was also robust, with an Area Under the ROC Curve (AUC / C-statistic) of 0.8801. Collinearity diagnostics via Variance Inflation Factors (VIF) showed that most baseline and clinical predictors were within safe limits (VIF < 3.0$). The elevated VIF values for WBC count (41.080) and Neutrophil count (33.075) are mathematically expected due to the intrinsic biological overlap between total leukocytes and their differential subsets. Although the VIF values for WBC and neutrophil count were elevated because of inherent biological dependence, these variables were retained due to their clinical relevance.

Predictors of Mortality Model:

Following the preference for Firth's penalized logistic regression due to low-event constraints, model diagnostics were evaluated using the Penalized Likelihood Ratio Test and the AUC. The overall multivariable model demonstrated an excellent fit to the data (chi-square = 42.575, df = 16, p < 0.001) based on a complete-case analysis. The model also exhibited outstanding

discrimination power, with an AUC / C-statistic of 0.9515. Multicollinearity assessment confirmed the total absence of problematic collinearity, with all VIF values remaining well below 5.0, specifically: AST (3.625), ALT (2.983), invasive ventilation (2.560), moderate-to-severe ARDS (2.035), ICU admission (1.973), serum creatinine (1.848), vasopressors (1.812), corticosteroids (1.735), HAIs (1.565), lymphocyte count (1.548), WBC count (1.517), male (1.514), comorbidities (1.389), age >60 (1.367), days from onset to admission (1.120), and double-dose oseltamivir or combination (1.087)."

Abstract

Predictors of ICU admission included being >60 years old (adjusted OR [AOR] 18.909, 95%CI 3.603–99.235, P<0.001), comorbidities (AOR 9.391, 95%CI 2.751–32.062, P<0.001), WBC (AOR 1.195, 95%CI 1.037–1.378, P=0.015), Neutrophil (AOR 11.334, 95%CI 1.856–69.22, P=0.009), Lymphocyte (AOR 9.092, 95%CI 1.342–61.588, P=0.024), and creatinine (AOR 1.032, 95%CI 1.005–1.059, P=0.019), AST (AOR 1.015, 95%CI 1.004–1.026, P=0.005), and ALT (AOR 0.987, 95%CI 0.974–0.999, P=0.037. Predictors of mortality were invasive ventilation (AOR 8.426, 95%CI 1.105-141.366, P=0.039) and double-dose oseltamivir or combination therapy (OR = 8.577, 95%CI 1.416 -83.341, P=0.020)

Materials and methods - Statistical analysis paragraph

"…A standard multivariable logistic regression for ICU admission prediction model was constructed. Model calibration and goodness-of-fit were formally evaluated using the Hosmer–Lemeshow test, while discrimination capacity was assessed via the Area Under the ROC Curve (AUC / C-statistic). Multicollinearity among predictors was monitored using Variance Inflation Factors (VIF). For mortality model, due to low-event constraints (sparse mortality data), Firth's penalized logistic regression was employed to minimize small-sample bias and avoid issues of separation. Complete-case analysis was applied to this model. Goodness-of-fit was evaluated using the Penalized Likelihood Ratio Test, and discrimination power was determined via the AUC / C-statistic. Multicollinearity was similarly assessed using VIF. Given the potential for confounding among covariates, the purposeful selection process was used to identify covariates for the regression models. Following the methodology described elsewhere (32, 33) , a more generous P-value cutoff of <0.25 in the univariate analysis was used. This approach was chosen because traditional cutoffs (such as 0.05) often fail to identify variables that, while not independently significant, act as important confounders or become significant when adjusted for other covariates. Furthermore, variables considered clinically important based on the authors' expertise were retained irrespective of their univariable statistical significance to ensure that potential confounding was appropriately addressed.

Results

Unadjusted risk factors for ICU admission and mortality among 158 patients with laboratory-confirmed A(H1N1)pdm09-related pneumonia

There was a statistically significant association between ICU admission and comorbidities (OR = 1.812, 95%CI 1.348–2.435, P<0.001), and laboratory parameters including serum creatinine (OR = 1.024, 95%CI 1.010–1.038, P<0.001), glycemia (OR = 1.007, 95%CI 1.001–1.014, P=0.026), AST (OR = 1.007, 95%CI 1.002–1.012, P=0.005), and serum albumin (OR = 0.775, 95%CI 0.654–0.918, P=0.003) (Table 4).

There was a statistically significant association between mortality and moderate-to-severe ARDS (P<0.001, OR = 23.170, 95%CI 2.905–184.789), laboratory parameters including AST (OR = 1.009, 95%CI 1.003–1.014, P<0.001), ALT (OR = 1.007, 95%CI 1.000–1.013, P=0.041), and serum albumin (OR = 0.774, 95%CI 0.615–0.975, P=0.030), and treatment including the use of double-dose oseltamivir or combination (OR = 4.792, 95%CI 1.471–15.612, P=0.009) as well as invasive ventilation (OR = 31.190, 95%CI 7.669–126.861, P<0.001). There was also a statistically significant association between mortality and the use of corticosteroids (OR = 5.684, 95%CI 1.725–18.728, P=0.004) and vasopressors (OR = 11.571, 95%CI 3.264–41.021, P<0.001), hospital-acquired infections (OR = 17.500, 95%CI 4.189–73.109, P<0.001), and ICU admission (OR=14.769, 95%CI 1.871–116.586, P=0.011) (table 5).

Models for the prediction of ICU admission and mortality among 158 patients with laboratory-confirmed A(H1N1)pdm09-related pneumonia

Predictors of ICU admission included being above 60 years old (AOR 18.909, 95%CI 3.603–99.235, P<0.001), presence of any recorded comorbidity (AOR 9.391, 95%CI 2.751–32.062, P<0.001), WBC (AOR 1.195, 95%CI 1.037–1.378, P=0.015), Neutrophil (AOR 11.334, 95%CI 1.856–69.22, P=0.009), Lymphocyte (AOR 9.092, 95%CI 1.342–61.588, P=0.024), Serum creatinine (AOR 1.032, 95%CI 1.005–1.059, P=0.019), AST (AOR 1.015, 95%CI 1.004–1.026, P=0.005), and ALT (AOR 0.987, 95%CI 0.974–0.999, P=0.037) (Table 6).

Predictors of mortality were invasive ventilation (AOR 8.426, 95%CI 1.105-83.366, P=0.039) and double-dose oseltamivir or combination therapy (AOR = 8.577, 95%CI 1.416 -83.341, P=0.020) (Table 7).

Table 5. Unadjusted risk factors tested for mortality among 158 patients with laboratory-confirmed A(H1N1)pdm09-related pneumonia

Variables	Mortality*		P value**	OR (95%CI)
	Yes (n=13)	No (n=145)		
Demographics				
Above 60 years old	3 (23.1)	21 (14.5)	0.414	1.771 (0.450–6.975)

Male	6 (48.2)	74 (51.0)	0.736	1.216 (0.390–3.794)
Comorbidities	4 (30.8)	44 (30.3)	0.975	0.98 (0.287–3.353)
Days from disease onset to admission	6 ± 1	4 ± 3	0.129	1.112 (0.970–1.275)
Moderate-to-severe ARDS	11 (84.6)	47 (32.4)	<0.001	23.170 (2.905–184.789)
Laboratory characteristics				
White blood cell (per 1000 cells/mm3)	7.35 ± 4.73	7.28 ± 4.38	0.956	1.004 (0.883–1.141)
Neutrophil (per 1000 cells/mm3)	6.17 ± 4.06	5.49 ± 3.8	0.540	1.043 (0.912–1.194)
Lymphocyte (per 1000 cells/mm3)	0.78 ± 0.5	1.2 ± 1.54	0.121	0.355 (0.096–1.313)
Platelet (per 1000 cells/mm3)	202.9 ± 105.1	181.5 ± 92.0	0.431	1.002 (0.997–1.007)
Serum creatinine (μmol/L)	92 ± 35	76 ± 29	0.068	1.015 (0.999–1.031)
Glycemia (g/L)	150 ± 55	115 ± 64	0.075	1.008 (0.999–1.017)
AST (U/L)	183 ± 99	78 ± 81	< 0.001	1.009 (1.003–1.014)
ALT (U/L)	89.39 ± 119.85	48.88 ± 49.96	0.041	1.007 (1.000-1.013)
Serum albumin (g/L)	27 ± 4	36 ± 11	0.030	0.774 (0.615–0.975)
Total bilirubin (μmol/L)	11.67 ± 2.61	14.28 ± 22.91	0.843	0.993 (0.922–1.068)
CRP (mg/dL)	121 ± 57.98	71.26 ± 60.7	0.287	1.012 (0.990–1.036)
Procalcitonin (ng/mL)	12.79 ± 23.29	2. 98 ± 5.19	0.165	1.068 (0.973–1.171)
Arterial lactate (mmol/L)	3.58 ± 3.22	2.22 ± 1.58	0.056	1.299 (0.993–1.699)
Chest radiographic characteristics				
Alveolar injuries***	13 (100)	140 (96.6)	1.000	1.093 (1.041–1.147)
Bilateral lung infiltrates****	8 (61.5)	96 (66.2)	0.734	0.817 (0.254–2.629)
Antiviral therapy				
Double dose oseltamivir or combination of oseltamivir with zanamivir or ribavirin*****	6 (46.2)	22 (15.2)	0.009	4.792 (1.471–15.612)
Respiratory support				
Invasive ventilation******	10 (76.9)	14 (9.7)	<0.001	31.190 (7.669–126.861)
Other treatments				
Prone ventilation	12 (92.3)	134 (92.4)	0.989	0.985 (0.117–8.294)
Corticosteroids	6 (46.2)	19 (13.1)	0.004	5.684 (1.725–18.728)
Vasopressors	6 (46.2)	10 (6.9)	< 0.001	11.571 (3.264–41.021)
Hospital-acquired infections	5 (38.5)	5 (3.4)	< 0.001	17.500 (4.189–73.109)
ICU admission (n = 77)	12 (92.3)	65 (44.8)	0.011	14.769 (1.871–116.586)

*N (%) for categorical variables and mean ± SD for continuous variables.

**Fisher's exact test for categorical variables and Student's t-test for continuous variables.

*** Compared with those who developed interstitial injuries

****Compared with those who developed unilateral lung infiltrates

*****Compared with those who received standard dose oseltamivir.

******Compared with those who received non-invasive respiratory supports including nasal cannula, mask, high-flow nasal cannula (HFNC), and c*ontinuous positive airway pressure* (CPAP) as well as those who did not receive respiratory support

Table 6. Logistic regression analysis of predictors of ICU admission among 158 patients with laboratory-confirmed A(H1N1)pdm09 pneumonia

Variables	P value	AOR (95%CI)
Above 60 years old	< 0.001	18.909 (3.603–99.235)
Male	0.434	1.570 (0.507–4.860)
Comorbidities	< 0.001	9.391 (2.751–32.062)
Day from onset to admission	0.207	1.088 (0.954–1.242)
WBC (per 1000 cells/mm^3)	0.015	1.195 (1.037–1.378)
Neutrophil(per 1000 cells/mm^3	0.009	11.334 (1.856–69.22)
Lymphocyte (per 1000 cells/mm^3)	0.024	9.092 (1.342–61.588)
Platelet cell (per 1000 cells/mm^3)	0.164	0.995 (0.988–1.002)
Serum creatinine (µmol/L)	0.019	1.032 (1.005–1.059)
AST (U/L)	0.005	1.015 (1.004–1.026)
ALT (U/L)	0.037	0.987 (0.974–0.999)

AOR: Adjusted odds ratio; ARDS: acute respiratory distress syndrome. * Model diagnostics: Hosmer–Lemeshow goodness-of-fit test: Chi-square = 3.978, df = 8, P = 0.859. The Area Under the ROC Curve (**AUC / C-statistic**) was **0.8801**.

Table 7. Firth's penalized logistic regression analysis of predictors of mortality among 158 patients with laboratory-confirmed A(H1N1)pdm09 pneumonia

Variables	P value	AOR (95%CI)
Above 60 years old	0.205	5.137 (0.408-98.998)
Male	0.976	1.030 (0.146-8.282)
Comorbidities	0.731	1.445 (0.181-14.109)
Days from onset to admission	0.123	1.169 (0.918-1.388)
WBC (per 1000 cells/mm^3)	0.187	0.848 (0.631-1.068)
Lymphocyte (per 1000 cells/mm^3)	0.647	1.121 (0.080-1.810)
Serum creatinine (µmol/L)	0.425	1.011 (0.985-1.041)
AST (U/L)	0.330	1.006 (0.994-1.017)
ALT (U/L)	0.875	0.999(0.986-1.012)
Moderate-to-severe ARDS	0.927	0.885 (0.053-12.097)
Invasive ventilation	0.039	8.426 (1.105-141.366)

Double dose oseltamivir (150mg/12h) or combination*	0.020	8.577 (1.416-83.341)
Corticosteroids	0.286	0.371 (0.045-2.218)
Vasopressors	0.796	1.270 (0.189-7.600)
Hospital acquired infection	0.217	0.262 (0.024-2.151)
ICU admission	0.336	3.661 (0.248-81.225)

AOR: Adjusted odds ratio; ARDS: acute respiratory distress syndrome. Chi-square = 42.575, df = 16, $P < 0.001 (Penalized likelihood ratio test). Multicollinearity assessment via Variance Inflation Factors (VIF) indicated no problematic collinearity (all VIFs < 5.0, ranging from 1.087 to 3.625; highest for AST and ALT). The Area Under the ROC Curve (AUC / C-statistic) was 0.9515.

*Combination: Oseltamivir with Zanamivir or Ribavirin

Discussion

We similarly observed an association between ALT, AST levels and ICU admission among our participants. Furthermore, our multivariable model demonstrated that elevated WBC, neutrophil, and lymphocyte counts, along with increased serum creatinine, were independent predictors of intensive care requirement. Considering these findings, patients with A(H1N1)pdm09 infection who present with advanced age, baseline comorbidities, liver enzyme elevation, acute kidney parameters, or marked leukocytosis and neutrophilia should be monitored closely and continuously for timely detection of complications and the need for ICU admission.

12. Minor Comments in Abstract

Comments:

1. **The abstract is too dense and numerically overloaded.**
2. **Confidence intervals should be formatted consistently.**
3. **Avoid causal wording regarding antiviral therapy.**

Suggested wording:

Instead of: > “Predictors of mortality were invasive ventilation and double-dose oseltamivir…”

Use: > “Mortality was associated with invasive ventilation and intensified antiviral therapy.”

Response: We thank the Reviewer for these helpful suggestions. We have revised the Abstract to improve readability by reducing numerical density, standardizing the presentation of confidence intervals, and replacing causal language with more appropriate associative wording. Following the revised multivariable analysis, the mortality findings reported in the Abstract have also been updated to reflect the final statistical results as follows.

Abstract

Background

The A(H1N1)pdm09 virus remains an important cause of severe influenza worldwide. This study examined the burden of ICU admission, mortality, and associated predictors among adults with A(H1N1)pdm09-related pneumonia treated at a leading infectious diseases hospital in Vietnam.

Methods

Demographic, clinical, laboratory, treatment, and outcome data were retrospectively collected from the medical records of adults hospitalized with laboratory-confirmed A(H1N1)pdm09 infection between 2009 and 2019.

Results

Among 729 patients with laboratory-confirmed A(H1N1)pdm09 infection, 21.7% (158/729) developed pneumonia. Among patients with pneumonia, 36.7% developed moderate-to-severe acute respiratory distress syndrome, 15.2% required invasive ventilation, 48.7% were admitted to the ICU, and 8.2% died. Independent predictors of ICU admission included age older than 60 years (AOR 18.91, 95% CI 3.60–99.24), the presence of comorbidities (AOR 9.39, 95% CI 2.75–32.06), and laboratory evidence of systemic inflammation, renal dysfunction, and hepatic involvement. Intensified antiviral therapy (OR = 8.577, 95%CI 1.416 -83.341, P=0.020) and invasive ventilation (AOR 8.426, 95%CI 1.105-141.366, P=0.039) were independently associated with mortality.

Conclusions

Mortality associated with A(H1N1)pdm09-related pneumonia remains substantial. Older patients, those with comorbidities, and those presenting with abnormal laboratory findings should be monitored closely for deterioration requiring intensive care. The study did not demonstrate improved outcomes with intensified antiviral regimens. Prospective studies are required before definitive treatment recommendations can be made.

13. Introduction

Comments:

1. **Several sentences are excessively long.**
2. **Grammar and spacing need improvement.**
3. **Some references are outdated or repetitive.**

Specific corrections:

- **“death associated with influenza” → “influenza-associated mortality”**
- **“death, suggesting age may play” → add clearer transition**
- **Remove formatting artifacts:**
    - **“[MH1.1][MH1.2][DBT(1.3]”**

Response: We sincerely thank the Reviewer for these constructive corrections on the Introduction. We have carefully revised the Introduction to improve grammar, sentence structure, spacing, and reference selection. We also removed the formatting artifacts identified by the Reviewer.

Introduction

The A(H1N1)pdm09 virus emerged in 2009 and triggered a global influenza pandemic associated with an estimated 123,000–203,000 deaths [1, 2]. The pandemic was declared over in August 2010, after which A(H1N1)pdm09 transitioned into an endemic seasonal strain co-circulating with influenza A(H3N2) and B viruses [3, 4]. While influenza seasonality is well defined in temperate regions, circulation patterns in tropical areas are often irregular, which may compromise the optimal timing and impact of vaccination programs [5]. Despite the availability of effective vaccines, A(H1N1)pdm09 remains a significant contributor to worldwide

influenza-related morbidity and mortality [6]. From October 2024 through May 2025 influenza activity was reported in all influenza transmission zones [7]. The predominant viruses varied across transmission zones and between countries [7]. A study conducted in China found that during 2010 and 2015, the influenza-associated mortality was 9.9 per 100,000 people, in which influenza A(H3N2) virus was the leading cause with a rate of 5.18 per 100,000 followed by A(H1N1)pdm09 with a rate of 2.9 per 100,000[8]. Additionally, the prevalence of influenza shifted between the Southern and Northern Hemispheres following the COVID-19 pandemic. Specifically, the post-pandemic peak of influenza in the Northern Hemisphere occurred as early as December, with the epidemic's duration increasing to 11 weeks. Meanwhile, in the Southern Hemisphere, the epidemic increased in length to 20 weeks. Because influenza A/B and SARS-CoV-2 share similar transmission pathways (via aerosolized droplets and contaminated surfaces) and clinical presentations, and typically circulate in overlapping seasonal outbreaks, their potential for co-infection poses a significant public health threat [9].

While most influenza infections resolve without treatment, some patients, especially high-risk groups, may develop serious complications that can lead to death[3]. High-risk groups include children younger than five years, the elderly, pregnant women, and individuals with compromised immune systems or chronic medical conditions [10, 11]. However, the literature reports inconsistent risk factors for severe outcomes, which may partly explain the observed between-country differences in A(H1N1)pdm09 mortality burden [12, 13]. A study involving 696 A(H1N1)pdm09 patients in Spain has concurred that immune deficiency and chronic cardiovascular diseases are risk factors for mortality. Interestingly, this study has found that older age is associated with lower ICU admission but a risk factor for death, suggesting that age may play a differential role in predicting ICU admission versus mortality [11]. Regarding pregnancy, given that this is a well-accepted risk factor for severity and mortality of A(H1N1)pdm09 pneumonia, the World Health Organization (WHO) recommends that pregnant women are prioritized for influenza vaccination [14]. Although some studies reported

differing mortality estimates among pregnant women [12], pregnancy remains an established high-risk condition for severe influenza outcomes.

The dissimilarities in identifying risk factors for A(H1N1)pdm09 pneumonia-related mortality worldwide may reflect differences in study design, timing, population age structures, ethnic composition, geographical settings, and vaccination coverage [15]. Frequent viral mutations that influence disease severity may further contribute to variability in findings [16]. Therefore, ongoing, robust, country-specific studies are needed to improve understanding of the risk factors of A(H1N1)pdm09 infection-related ICU admission and mortality. However, long-term influenza surveillance remains limited in tropical regions of the Asia-Pacific due to gaps in information technology and available resources, with surveillance systems largely focusing on basic epidemiological data but lacking clinical and outcome information [17, 18]. Most studies in these areas are short-term (typically 1–2 years) [19], which is insufficient for effective monitoring, as influenza circulation in tropical settings tends to be sporadic and unpredictable. Establishing a comprehensive, nationwide surveillance system requires substantial financial investment, trained personnel, and infrastructure capable of collecting and integrating individual-level data. Moreover, because many countries in the region face competing public health priorities [20], maintaining long-term influenza surveillance systems is often challenging and may not be feasible or sustainable [19, 21]. In Vietnam, influenza viruses co-circulated all year round and were an important cause of influenza-like illness between 2006 and 2010 [22]. The first H1N1 vaccine became available locally in 2010, followed by other vaccines targeting both seasonal influenza strains and A(H1N1)pdm09. However, these vaccines are accessible primarily on a self-paid basis, even for high-risk groups. To strengthen influenza prevention and control in Vietnam and similar settings, continual assessment of disease severity is needed [22]. This study aimed to examine the clinical burden and identify predictors of severe disease among adult patients with A(H1N1)pdm09-related pneumonia. The primary outcomes were ICU admission and all-cause mortality. The secondary outcomes included respiratory

support requirements (such as invasive ventilation), development of moderate-to-severe ARDS, occurrence of hospital-acquired infections, and overall hospital and ICU lengths of stay.

14. Materials and Methods

Comments:

1. **Clarify whether consecutive sampling was used.**
2. **Explain how missing data were handled.**
3. **State whether all laboratory tests were performed at admission.**
4. **Mention whether coinfections were assessed.**
5. **Clarify why patients aged 17 years were classified as adults.**

Important: The methodology for purposeful variable selection should be described more concisely.

Response: We thank the Reviewer for these helpful comments. We have revised the Materials and Methods section to clarify the study design and improve methodological transparency. Specifically:

(1) We clarified that all eligible hospitalized patients meeting the inclusion criteria during the study period were enrolled consecutively;

(2) We added a description of the handling of missing data;

(3) We clarified that baseline laboratory investigations used in the analyses were performed at hospital admission unless otherwise specified;

(4) We clarified how bacterial and fungal coinfections were assessed; and

(5) We explained that patients aged 17 years or older were classified as adults according to the hospital admission policy and national clinical practice during the study period.

We also shortened the description of the purposeful variable selection strategy as suggested. Our revisions are as follows.

Materials and methods

Study design and context

A 10-year retrospective cohort study was conducted at the Hospital for Tropical Diseases (HTD) in Ho Chi Minh City from 1st January 2009 to 31st December 2019. The study was approved by the Ethics Committee of HTD (approval number 43/HĐĐĐ). Data were accessed for research purposes from 07/11/2019 to 30/04/2020. HTD is the 550-bed referral hospital for infectious diseases in southern Vietnam, receiving patients from across this region, including the Mekong Delta, with approximately 3,000 patients accessing healthcare services daily [23]. HTD's medical records of hospitalized patients are standardized in accordance with the Vietnam Ministry of Health's guidelines [24].

All eligible hospitalized patients meeting the inclusion criteria during the study period were enrolled consecutively. The inclusion criteria included persons aged 17 years and older (referred to here as 'adults', according to the hospital admission policy and national clinical practice during the study period.), having a positive reverse transcription polymerase chain reaction (RT-PCR) result with A(H1N1)pdm09 from nasal or oropharyngeal swab, and developing pneumonia confirmed by chest x-rays. There were no exclusion criteria.

Data collection

A data collection form was used to record participants' information from HTD's paper-based medical records. Data included demographics (age, gender, comorbidity, and pregnancy status of females), clinical signs and symptoms, chest x-ray features, laboratory test results, treatments, complications, and outcomes. Laboratory characteristics included complete blood count, serum creatinine, alanine transaminase (ALT), aspartate transaminase (AST), total bilirubin, glycemia, serum albumin, C-reactive protein (CRP), procalcitonin, and arterial lactate. Baseline laboratory investigations used in the analyses were performed at hospital admission unless otherwise specified. For missing data, percentages for categorical variables were calculated based on valid cases (available data), and the actual denominator for each variable was specified where missing data occurred. Microbiological specimens were collected and investigated only when clinically suspected and indicated, rather than systematically for all patients.

Statistical analysis

A standard multivariable logistic regression for ICU admission prediction model was constructed. Model calibration and goodness-of-fit were formally evaluated using the Hosmer–Lemeshow test, while discrimination capacity was assessed via the Area Under the ROC Curve (AUC / C-statistic). Multicollinearity among predictors was monitored using Variance Inflation Factors (VIF). For mortality model, due to low-event constraints (sparse mortality data), Firth's penalized logistic regression was employed to minimize small-sample bias and

avoid issues of separation. Complete-case analysis was applied to this model. Goodness-of-fit was evaluated using the Penalized Likelihood Ratio Test, and discrimination power was determined via the AUC / C-statistic. Multicollinearity was similarly assessed using VIF. Given the potential for confounding among covariates, the purposeful selection process was used to identify covariates for the regression models. Following the methodology described elsewhere (34, 35) , a more generous P-value cutoff of <0.25 in the univariate analysis was used. This approach was chosen because traditional cutoffs (such as 0.05) often fail to identify variables that, while not independently significant, act as important confounders or become significant when adjusted for other covariates. Furthermore, variables considered clinically important based on the authors' expertise were retained irrespective of their univariable statistical significance to ensure that potential confounding was appropriately addressed.

15. Results

Comments:

1. Ensure consistency in percentages and decimal places.

2. Tables should be simplified.

3. Some variables are repeated unnecessarily.

4. Clarify discrepancy:

o Mortality reported as 13 in some sections and 12 in Table 4.

This inconsistency must be corrected.

Response: We thank the Reviewer for identifying these inconsistencies. We have carefully reviewed the Results section and all tables to ensure complete consistency throughout the manuscript.

- Decimal and Percentage Consistency: We have meticulously reviewed all percentages and decimal places throughout the text and tables, standardizing them to a consistent format (e.g., rounding percentages and odds ratios uniformly).
- Table Simplification and Redundant Variables: We have simplified Table 4, Table 5, and Table 6 by removing unnecessary, redundant variables, reducing clutter, and improving overall scannability for the reader.
- Correction of Mortality Discrepancy: The Reviewer is entirely correct. The absolute number of deaths in this cohort is 13 (resulting in a mortality rate of 8.2%). The value of 12 previously listed in Table 4 was a typographical error. We have corrected this cell in Table 4, updated the associated percentages in that column, and re-verified that all

tables (including Table 6) and prose throughout the Abstract and Results sections uniformly report 13 deaths.

16. **Discussion**

Comments:

1. **Some paragraphs are overly descriptive.**
2. **Discussion should focus more on interpretation rather than repeating results.**
3. **Comparison with previous literature should be more balanced.**
4. **Avoid overstating vaccine effects because vaccination status was unavailable.**

Important conceptual issue:

The discussion frequently implies causality from observational associations.

Please use more cautious language such as:

- **"associated with"**
- **"may reflect"**
- **"possibly related to"**

instead of:

- **"caused"**
- **"increased risk due to"**
- **"should not be recommended"**

Response: We thank the Reviewer for these valuable suggestions. We have substantially revised the Discussion to improve its interpretive focus, reduce repetition of the Results, and adopt more cautious language throughout. In particular, we have removed statements implying causality, moderated the discussion regarding vaccination because individual vaccination status was unavailable, and revised the interpretation of intensified antiviral therapy to acknowledge the likelihood of confounding by indication. The corresponding revisions are highlighted below.

Discussion – 2nd paragraph

“During the study period, more than two-thirds inpatients infected with A(H1N1)pdm09 influenza were documented in 2009 when A(H1N1)pdm09 emerged [3]. … Information on the uptake of seasonal influenza vaccine in Vietnam is limited. Nevertheless, a local study conducted on 750 people in the general community found that 30% of participants received a seasonal flu vaccine either in the current or preceding flu season, and 64% expressed demand for this vaccine [37]. Considering this, the reduction in the number of hospitalized A(H1N1)pdm09 cases over time may partly reflect increasing population immunity, including possible contributions from vaccination and prior exposure, although these factors could not be evaluated directly because individual vaccination status was unavailable in this study [37]. The underlying health conditions, such as cardiovascular diseases, diabetes and pregnancy, may exacerbate the severity of A(H1N1)pdm09 infection [38].

Discussion – 4th paragraph

“…Despite effective treatment and vaccines, our study confirms that the mortality due to A(H1N1)pdm09-related pneumonia remains substantial…”

Discussion – 5th paragraph

“…We found that age above 60 years and the presence of comorbidities were associated with ICU admission…”

Discussion – 6th paragraph

“…The observed association between intensified antiviral therapy and mortality most likely reflects confounding by indication. Patients with more severe disease were preferentially treated with intensified antiviral regimens rather than a direct harmful effect of the therapy itself. Current evidence does not demonstrate a clear survival benefit from intensified antiviral regimens in severe influenza, and prospective studies are needed before definitive conclusions regarding their clinical effectiveness can be drawn…”

17. Conclusion

The conclusion is generally appropriate but currently too strong regarding antiviral therapy.

Suggested revision:

Replace: > “Neither double-dose oseltamivir nor combination therapy is recommended…”

With: > “The study did not demonstrate improved outcomes with intensified antiviral regimens; however, prospective studies are required before definitive treatment recommendations can be made.”

Response: We thank the Reviewer for this important suggestion. We agree that our original conclusion was stronger than could be supported by the retrospective observational design of this study. Accordingly, we have removed the treatment recommendation regarding intensified antiviral therapy and replaced it with a more cautious interpretation that is consistent with both our findings and the limitations of the study design. The revised conclusion now reads:

Conclusion

The study did not demonstrate improved outcomes with intensified antiviral regimens. However, prospective studies are required before definitive treatment recommendations can be made

18. Language and Formatting Corrections

The manuscript requires careful English editing.

Examples:

**Current Text**	**Suggested Revision**
“death associated with influenza”	“influenza-associated mortality”
“risk factors of A/(H1N1)pdm09 infection-related ICU admission”	“risk factors for ICU admission related to A(H1N1)pdm09 infection”
“patients demanded respiratory support”	“patients required respiratory support”
“liver affection”	“hepatic involvement”
“timely detection of complications requiring intensive care”	“early identification of complications requiring intensive care”

Response: We thank the Reviewer for these valuable editorial suggestions. We have carefully edited the manuscript throughout to improve grammar, terminology, and consistency of scientific language. The examples identified by the Reviewer have all been corrected, including those shown below.

Introduction – 1st paragraph

“…A study conducted in China found that during 2010 and 2015, the influenza-associated mortality was 9.9 per 100,000 people....”

Discussion – 4th paragraph

“…Our participants required respiratory support at different levels, including non-invasive and invasive methods…”

Discussion – 5th paragraph

"…Other studies also found an elevation of both AST and ALT in several A(H1N1)pdm09 infected patients, probably due to hepatic involvement in the pathogenesis of influenza [34]…."

"… Considering our findings, patients with A(H1N1)pdm09 infection who have at least one of the following factors: advanced age, comorbidities, liver enzyme elevation, or moderate-to-severe ARDS should be monitored closely and continuously for early identification of complications requiring intensive care…."

19. Strengths of the Study

1. **Long study duration (10 years)**
2. **Valuable tropical-region influenza data**
3. **Real-world clinical dataset**
4. **Important information regarding ICU burden and ARDS**
5. **Inclusion of laboratory and treatment variables**
6. **Relevant topic for infectious disease and critical care literature**

Response: We sincerely thank the Reviewer for these encouraging comments and for recognizing the core strengths of our study. We agree that providing a robust, real-world clinical dataset spanning a 10-year period offers unique and valuable insights into the long-term patterns of A(H1N1)pdm09 pneumonia within a tropical region. Capturing comprehensive laboratory data, treatment variables, and the specific burdens of severe ARDS and ICU admissions reflects the everyday realities of critical care management at our center. Your positive evaluation strongly encourages our team to continue advancing our research in infectious diseases and critical care medicine.

20. Reference Review and Citation Assessment

Overall Assessment of References

The manuscript includes a broad range of references from WHO, CDC, peer-reviewed journals, and regional influenza studies. Most references are relevant and support the general scientific background appropriately. However, several citations are either:

- **Weakly connected to the statement they support**
- **Overinterpreted**

- **Outdated when newer evidence exists**
- **Not optimally matched to the conclusions being drawn**

In multiple sections, the discussion extrapolates beyond what the cited literature directly demonstrates.

Response: We sincerely thank the Reviewer for this critical and constructive assessment of our literature citations. We agree that a more rigorous, precisely matched, and up-to-date reference list is essential to support our conclusions without overinterpretation. In response, we systematically reviewed every citation in the manuscript to ensure that each reference directly supports the corresponding statement and replaced outdated or less appropriate references where necessary. All updated and newly added references have been correctly cross-referenced in the finalized reference list and highlighted within the revised text. For example, we would like to replace citation number 28 as follows.

Reference

28. MinistryofHealthVietnam, National guideline of diagnosis, treatment and prevention for A/H1N1 influenza (2009).

With

28. Ministry of Health Vietnam, National guideline of diagnosis, treatment and prevention for A/H1N1 influenza (2025).

21. Detailed Reference-to-Text Evaluation

Mortality and Global Burden Statements

Text:

“The A(H1N1)pdm09 virus remains a major global health threat.”

References Used:

- **WHO (Ref 1)**
- **Simonsen et al. (Ref 2)**
- **CDC (Ref 3)**

Review:

These references appropriately support pandemic burden and historical mortality. However, the wording "major global health threat" may be slightly overstated in the current post-pandemic era unless supported with more recent burden data.

Suggestion:

Include newer global influenza burden references (2022–2025), especially post-COVID influenza resurgence studies.

Response: We thank the Reviewer for this important observation. As suggested, we have updated our literature review to incorporate recent global burden data (2022–2025), focusing specifically on the post-COVID-19 influenza resurgence and its altered epidemiological patterns. We have added this recent context and the corresponding references to the Introduction section to justify our statement:

Introduction – 1st paragraph

"…A study conducted in China found that during 2010 and 2015, the influenza-associated mortality was 9.9 per 100,000 people, in which influenza A(H3N2) virus was the leading cause with a rate of 5.18 per 100,000 followed by A(H1N1)pdm09 with a rate of 2.9 per 100,000[8]. Additionally, the prevalence of influenza shifted between the Southern and Northern Hemispheres following the COVID-19 pandemic. Specifically, the post-pandemic peak of influenza in the Northern Hemisphere occurred as early as December, with the epidemic's duration increasing to 11 weeks. Meanwhile, in the Southern Hemisphere, the epidemic increased in length to 20 weeks. Because influenza A/B and SARS-CoV-2 share similar transmission pathways (via aerosolized droplets and contaminated surfaces) and clinical presentations, and typically circulate in overlapping seasonal outbreaks, their potential for co-infection poses a significant public health threat [9]. …."

22. Tropical Influenza Seasonality

Text:

"Influenza circulation patterns in tropical areas are often irregular…"

References Used:

- **Muscatello et al. (Ref 5)**
- **Vietnam surveillance study (Ref 21)**

Review:

These references are appropriate and well-matched.

Strong citation alignment.

Response: We sincerely thank the Reviewer for validating our citation alignment on this point. We are glad that the selected references appropriately and robustly support our description of the irregular influenza circulation patterns characteristic of tropical regions.

23. **Risk Factors for Severe Disease**

Text:

“High-risk groups include children younger than five years, the elderly, pregnant women…”

References Used:

- **Ref 8**
- **Ref 9**
- **Ref 10**

Review:

Ref 8 (Miyazawa) mainly discusses overlap between COVID-19 and H1N1 metabolic risk factors and is not the strongest citation for classical influenza high-risk groups.

Better references:

- **WHO influenza risk group guidance**
- **CDC influenza high-risk population guidance**
- **Mertz et al. systematic review (already included as Ref 14)**

Recommendation:

Replace Ref 8 here with stronger epidemiological guidance references.

Response: We appreciate this thoughtful suggestion. Reference 8 (Miyazawa) focuses heavily on the metabolic overlap between COVID-19 and H1N1, making it less ideal for supporting classical influenza high-risk groups. As recommended, we have removed Reference 8 from this sentence and replaced it with the systematic review by Mertz et al. (which is already utilized elsewhere in the manuscript).

The citation list for this statement has been updated accordingly in the revised manuscript to ensure the high-risk categories are robustly supported.

Introduction – 2nd paragraph

High-risk groups include children younger than five years, the elderly, pregnant women, and individuals with compromised immune systems or chronic medical conditions [10, 11].

24. Pregnancy and Severity

Text:

“Pregnant women are prioritized for influenza vaccination.”

References Used:

- **WHO vaccination guidance (Ref 13)**

Review:

Appropriate and well-supported.

However:

Text:

“Mexican study found reduced fatality rate among pregnant women…”

Reference:

- **Chowell et al. (Ref 11)**

Concern:

The manuscript uses this finding to suggest variability in severity associations across countries. While technically true, this statement requires more nuanced interpretation because:

- **Pregnancy is still globally accepted as a high-risk condition.**
- **The Mexican findings may reflect healthcare access, earlier hospitalization, or surveillance differences.**

Suggestion:

Avoid creating doubt regarding pregnancy risk status.

Suggested wording:

“Although some studies reported differing mortality estimates among pregnant women, pregnancy remains an established high-risk condition for severe influenza outcomes.”

Response: We thank the Reviewer for this important observation. Interpretation of pregnancy should be clearer to prevent readers from misunderstanding the impact of pregnancy on influenza severity.

Introduction – 2nd paragraph

Regarding pregnancy, given that this is a well-accepted risk factor for severity and mortality of A(H1N1)pdm09 pneumonia, the World Health Organization (WHO) recommends that pregnant women are prioritized for influenza vaccination [14]. Although some studies reported differing mortality estimates among pregnant women [12], pregnancy remains an established high-risk condition for severe influenza outcomes.

25. **Surveillance Challenges in Tropical Regions**

References:

- **Refs 16–20**

Review:

Excellent and well-integrated references.

These strongly support the rationale for the study.

Response: We sincerely thank the Reviewer for validating our citation alignment on this point. We are pleased that the selected references appropriately and robustly support our rationale regarding the unique surveillance challenges faced in tropical regions.

26. **Vaccination Discussion**

Text:

“Decrease in inpatient cases over time is probably attributable to vaccination and herd immunity.”

References:

- **Refs 34–36**

Major Concern:

This conclusion is speculative.

The cited references discuss vaccine uptake and influenza training but do NOT directly demonstrate causality between vaccination and reduced admissions in this cohort.

Additionally:

- **Vaccination status was unavailable.**
- **No population-level vaccination coverage trend analysis was performed.**

Recommendation:

Tone down interpretation.

Suggested revision:

“The reduction in hospitalized cases over time may partly reflect increasing population immunity, including possible contributions from vaccination and prior exposure.”

Response: We sincerely thank the Reviewer for this crucial critique. We completely agree that our initial conclusion regarding vaccination and herd immunity was overly speculative, given that individual vaccination status was unavailable and no population-level coverage trend analysis was performed.

As recommended, we have toned down our interpretation and adopted your suggested phrasing to accurately reflect the limits of our observational data. Furthermore, we have expanded this section to briefly clarify the local immunization landscape to give readers context as to why coverage data are limited.

Discussion – 2nd paragraph

“…the reduction in the number of hospitalized A(H1N1)pdm09 cases over time may partly reflect increasing population immunity, including possible contributions from vaccination and prior exposure, although these factors could not be evaluated directly because individual vaccination status was unavailable in this study [37]...Currently, flu vaccines are not part of the country’s broader immunization efforts [36]…”

27. Radiological Findings Compared with COVID-19

References:

- **Ref 39**
- **Ref 40**

Review:

Appropriate and relevant.

The discussion is balanced and clinically meaningful.

Response: We sincerely thank the Reviewer for this positive evaluation. We are glad that the radiological comparisons between A(H1N1)pdm09 and COVID-19 were found to be balanced, relevant, and clinically meaningful for the critical care context.

28. Early Oseltamivir Therapy

Text:

"Early treatment with oseltamivir can reduce lower respiratory tract complications."

Reference:

- **Moreno et al. (Ref 41)**

Concern:

Ref 41 specifically evaluated critically ill influenza pneumonia patients and survival benefit from early oseltamivir.

The manuscript slightly generalizes this to all adult influenza patients.

Suggestion:

Clarify patient population.

Response: We completely agree with the Reviewer's assessment. Reference 41 (Moreno et al.) specifically focused on critically ill patients with established influenza pneumonia, meaning its extrapolation to the broader adult influenza population was imprecise. To accurately clarify the target patient population, we have replaced Reference 41 with two more appropriate references that confirm the impact of early oseltamivir on lower respiratory tract complications across standard clinical settings:

1. Hernán MA, Lipsitch M. Oseltamivir and risk of lower respiratory tract complications in patients with flu symptoms: a meta-analysis of eleven randomized clinical trials. *Clin Infect Dis.* 2011;53(3):277-279.
2. Centers for Disease Control and Prevention. Influenza Antiviral Medications: Summary for Clinicians. 2026; https://www.cdc.gov/flu/hcp/antivirals/summary-clinicians.html. Accessed 28th June, 2026.

29. International Mortality Comparisons

References:

- **India (Ref 42)**
- **Singapore (Ref 43)**
- **Vietnam (Ref 45)**

Review:

These comparisons are relevant.

However, mortality comparisons between countries should acknowledge:

- **Different hospitalization thresholds**
- **ICU availability**
- **Diagnostic criteria**
- **Population structures**
- **Healthcare systems**

Suggestion:

Explicitly mention that direct comparison should be interpreted cautiously.

Response: We appreciate this thoughtful suggestion. Direct international comparisons of mortality rates must be interpreted with caution due to baseline differences in institutional structures and demographics.

As suggested, we have revised this section of the Discussion to explicitly state these limitations. We have incorporated a balanced discussion addressing how varying hospitalization thresholds, ICU capacity, population structures, and healthcare system disparities across different countries impact reported mortality metrics.

Discussion – 4th paragraph

"…Despite effective treatment and vaccines, our study confirms that the mortality due to A(H1N1)pdm09-related pneumonia remains substantial. It is comparable to that of a study conducted in India (8543/114667, 7.5%, 95% CI 7.3–7.6%) during a similar time period [46] and of a study conducted in the North of the country during 2015-2017 (25/259, 9.3%, 95%CI 6.0–13.7%) [47]. In contrast, our mortality is lower than that of a study conducted in Singapore

(41/172, 23.8%, 95%CI 18.1–30.7%) between June 2009 and August 2010 [48]. Direct comparisons of mortality estimates between countries should be interpreted cautiously. This is because differences in hospitalization thresholds, ICU capacity, healthcare systems, diagnostic practices, and patient population characteristics may contribute to the observed variation. Further comparative studies using standardized methodologies are needed to better understand these differences. Nevertheless, the mortality rate due to A(H1N1)pdm09 infection varies between countries, although it may be lower in developed countries, due to history of vaccination, differences in climate patterns, population density, and population age structure, as well as countries' economic status and development [49]. In Vietnam, our mortality rate is similar to that of a study conducted in the North of the country during 2015-2017 (25/259, 9.3%, 95%CI 6.0–13.7%) [47]. In light of our findings, mortality due to A(H1N1)pdm09-related pneumonia is not rare, despite effective treatment and vaccine.."

30. AST/ALT and Liver Involvement

References:

- **Ref 47**

Review:

Appropriate citation.

However, the phrase "liver affection" is non-standard English.

Use:

- **"hepatic involvement"**
- **"influenza-associated hepatic injury"**

Response: We thank the Reviewer for this valuable linguistic correction. We agree that the term "liver affection" is non-standard, and we have systematically replaced it with **"hepatic involvement"** or **"hepatic injury"** throughout the revised manuscript to ensure proper medical terminology.

The corresponding passage in the **Discussion** has been corrected as follows:

Discussion – 5th paragraph

"…Other studies also found an elevation of both AST and ALT in several A(H1N1)pdm09 infected patients, probably due to hepatic involvement in…".

31. Invasive Ventilation and Mortality

References:

- **Refs 48–51**

Review:

These references appropriately support the discussion. However, the manuscript sometimes implies invasive ventilation itself causes mortality. This is problematic because invasive ventilation is primarily a marker of disease severity.

Recommendation:

Clarify:

"The association likely reflects underlying disease severity among patients requiring invasive respiratory support."

Response: We totally agree with the Reviewer that interpretation of invasive ventilation required more caution since it is a marker of disease severity rather than an independent factor associated with or caused mortality. We would write these sentences with more generalization as:

Discussion – 6th paragraph

"…We found that mortality is strongly associated with invasive ventilation, which is consistent with findings from other studies [41, 52]. Indeed, not all patients with influenza-related pneumonia undergo invasive ventilation, but those with more severe complications, including ARDS, are often given this type of ventilation support within 3–10 days following initial symptom onset [53]. The association likely reflects underlying disease severity among patients requiring invasive respiratory support. Thus, studies are ongoing to identify the feasible and protective ventilation protocol for patients with influenza-related pneumonia requiring mechanical ventilation [54, 55]..."

32. Double-Dose Oseltamivir and Combination Therapy

References:

- **Refs 52–54**

Major Concern:

The manuscript selectively interprets these references.

The cited studies mainly conclude:

- **No clear mortality benefit**
- **Limited evidence**
- **Confounding in observational studies**

But the manuscript concludes: > "Neither double-dose oseltamivir nor combination therapy is recommended."

This is stronger than the evidence.

Recommendation:

The discussion should instead state:

"Current evidence does not demonstrate a clear survival benefit from intensified antiviral regimens in severe influenza."

Avoid treatment recommendations beyond observational evidence.

Response: We thank the Reviewer for this important comment. We agree that our observational study does not support definitive treatment recommendations. Therefore, we have removed the previous recommendation against intensified antiviral regimens and replaced it with a more cautious interpretation that reflects the limitations of the study design. The revised Conclusion now reads:

Discussion – 6th paragraph

"…The observed association between intensified antiviral therapy and mortality most likely reflects confounding by indication. Patients with more severe disease were preferentially treated with intensified antiviral regimens rather than a direct harmful effect of the therapy itself. Current evidence does not demonstrate a clear survival benefit from intensified antiviral regimens in severe influenza, and prospective studies are needed before definitive conclusions regarding their clinical effectiveness can be drawn…"

Conclusion

"…The study did not demonstrate improved outcomes with intensified antiviral regimens. However, prospective studies are required before definitive treatment recommendations can be made…"

33. Age and Mortality Discussion

References:

- **Ref 55**
- **Ref 56**

Review:

Appropriate use of literature.

The manuscript appropriately acknowledges heterogeneity across countries.

This section is scientifically balanced.

Response: We sincerely thank the Reviewer for this positive evaluation. We are glad that our discussion regarding age-related mortality and the associated inter-country heterogeneity was found to be scientifically balanced and appropriately supported by the selected literature.

34. Reference Formatting Issues

Several formatting inconsistencies were identified:

Examples:

1. **WHO references inconsistently formatted.**
2. **Some URLs are broken across lines.**
3. **"World Health Organisation" and "World Health Organization" used interchangeably.**
4. **Journal title capitalization inconsistent.**
5. **Some references missing DOI information.**
6. **Reference 23 is incomplete and poorly formatted.**

7. **Reference 36 appears mismatched:**

o **"Training on Influenza Data Management and Basic Analysis: Maldives 2023" does not strongly support herd immunity/vaccination discussion.**

Recommendation:

The reference list should be professionally reformatted according to journal style.

Response: We sincerely thank the Reviewer for pointing out these formatting inconsistencies and apologize for the oversights in our initial draft. We have systematically overhauled the entire reference list to ensure compliance with professional publishing standards:

- Style standardization: We have applied the official, updated PLOS ONE citation style across the entire bibliography using reference management software. This has successfully resolved all issues regarding inconsistent journal title capitalization, broken URLs, and missing DOIs.
- Nomenclature uniformity: All instances of the World Health Organization have been standardized uniformly to "World Health Organization" (US English spelling).
- Correction of reference 23: Reference 23 has been carefully cross-checked, completed, and reformatted to meet journal specifications.
- Replacement of mismatched reference 36: The reviewer is entirely correct that the Maldives 2023 data management training document did not appropriately support the discussion on herd immunity and vaccination. We have removed this citation and replaced it with an authoritative, highly relevant publication: 1. Tenforde MW, Reeves EL, Weber ZA, et al. Influenza Vaccine Effectiveness Against Hospitalizations and Emergency Department or Urgent Care Encounters for Children, Adolescents, and Adults During the 2023-2024 Season, United States. Clin Infect Dis. 2025;81(3):667-678.

Discussion – 2nd paragraph

Considering this, the reduction in the number of hospitalized A(H1N1)pdm09 cases over time may partly reflect increasing population immunity, including possible contributions from vaccination and prior exposure, although these factors could not be evaluated directly because individual vaccination status was unavailable in this study [37]…. Despite this significant risk, most SEAR countries lack comprehensive seasonal influenza vaccination policies, resulting in low vaccine uptake across the region [39]. Currently, flu vaccines are not part of the country's broader immunization efforts [37].

35. Additional Literature Recommended

The manuscript would benefit from citing:

1. **Recent WHO influenza surveillance reports (2023–2025)**
2. **Post-COVID influenza epidemiology studies**
3. **Updated antiviral treatment guidelines**
4. **Studies on influenza-vaccination effectiveness in Southeast Asia**
5. **Modern ARDS management guidelines**

Response: We sincerely appreciate the Reviewer's excellent recommendations for additional literature. We agree that integrating these specific surveillance reports, epidemiological studies, and clinical guidelines significantly elevates the depth and contemporary relevance of our findings.

We have incorporated the recommended contemporary literature where appropriate to strengthen the Introduction, Discussion, and interpretation of our findings as detailed below:

1. Recent WHO influenza surveillance reports (2023–2025)

Introduction – 1st paragraph

"…While influenza seasonality is well defined in temperate regions, circulation patterns in tropical areas are often irregular, which may compromise the optimal timing and impact of vaccination programs [5]. Despite the availability of effective vaccines, A(H1N1)pdm09 remains a significant contributor to worldwide influenza-related morbidity and mortality [6]. From October 2024 through May 2025 influenza activity was reported in all influenza transmission zones [7]. The predominant viruses varied across transmission zones and between countries [7]…"

2. Post-COVID influenza epidemiology studies

Introduction – 1st paragraph

"…A study conducted in China found that during 2010 and 2015, the influenza-associated mortality was 9.9 per 100,000 people, in which influenza A(H3N2) virus was the leading cause with a rate of 5.18 per 100,000 followed by A(H1N1)pdm09 with a rate of 2.9 per 100,000[8]. Additionally, the prevalence of influenza shifted between the Southern and Northern Hemispheres following the COVID-19 pandemic. Specifically, the post-pandemic peak of influenza in the Northern Hemisphere occurred as early as December, with the epidemic's duration increasing to 11 weeks. Meanwhile, in the Southern Hemisphere, the epidemic increased in length to 20 weeks. Because influenza A/B and SARS-CoV-2 share similar

transmission pathways (via aerosolized droplets and contaminated surfaces) and clinical presentations, and typically circulate in overlapping seasonal outbreaks, their potential for co-infection poses a significant public health threat [9]….”

3. Updated antiviral treatment guidelines

Discussion – 6th paragraph

“…Instead, priority should be given to improving early diagnosis, ensuring timely antiviral treatment, and using appropriate antimicrobials in patients with secondary bacterial infection, rather than initiating double-dose oseltamivir or combination antiviral therapy [59, 60]...”

4. Studies on influenza-vaccination effectiveness in Southeast Asia

Discussion – 2nd paragraph

Despite this significant risk, most SEAR countries lack comprehensive seasonal influenza vaccination policies, resulting in low vaccine uptake across the region [39]. Currently, flu vaccines are not part of the country’s broader immunization efforts [37].

5. Modern ARDS management guidelines

Discussion – 6th paragraph

Thus, studies are ongoing to identify the feasible and protective ventilation protocol for patients with influenza-related pneumonia requiring mechanical ventilation [54, 55]..

36. Most Scientifically Strong Sections

The following areas are well-supported by references:

- **Influenza surveillance gaps in tropical regions**
- **ARDS definitions and ICU indications**
- **Radiological overlap with COVID-19**
- **Mechanical ventilation discussions**
- **Regional mortality comparisons**

Response: We sincerely thank the Reviewer for identifying these areas as the scientifically strongest components of our manuscript.

We are pleased that our synthesis of tropical influenza surveillance gaps, critical care parameters (ARDS definitions, ICU indications, and mechanical ventilation), and radiological comparisons with COVID-19 resonated well with the clinical scope of your review. Grounding these observations in robust regional mortality data was a priority for our team, and your positive validation of these sections is highly encouraging as we finalize this work.

37. Sections Requiring Stronger Evidence or More Careful Interpretation

1. **Vaccine-related reduction in hospitalizations**
2. **Interpretation of intensified antiviral therapy**
3. **Implication that invasive ventilation contributes directly to mortality**
4. **Pregnancy-related severity interpretation**
5. **Generalization of oseltamivir evidence**

Response: We sincerely thank the Reviewer for highlighting these specific focus areas. As detailed in our individual responses to your previous comments, we have systematically addressed every point listed here. Specifically, we have toned down all causal language, avoided over-generalizing the oseltamivir and pregnancy data, contextualized the vaccine constraints in Southeast Asia, and carefully re-framed the discussion surrounding invasive mechanical ventilation as a marker of critical severity rather than a direct driver of mortality.

38. Final Recommendation

Recommendation: Major Revision

The manuscript addresses an important clinical and epidemiological topic and contains valuable long-term data from Vietnam. However, substantial revisions are required, particularly regarding:

- **Statistical robustness**
- **Interpretation of antiviral therapy findings**
- **Clarification of methodology**
- **Reduction of causal language**
- **Improved discussion of limitations**

- **English language editing**

With these revisions, the manuscript could make a meaningful contribution to influenza outcome research in tropical settings.

Additionally, although the long-term dataset is valuable, the study period (2009–2019) raises concerns regarding the contemporary applicability of the findings. A substantial proportion of cases were recorded during the 2009 pandemic phase, while influenza epidemiology, vaccination coverage, antiviral strategies, ICU management protocols, diagnostic capabilities, and supportive care practices have evolved considerably over time. Therefore, the generalizability of these findings to current clinical settings may be limited. The manuscript would benefit from a more detailed discussion on temporal heterogeneity and changes in clinical management across the study period. Stratified analyses comparing pandemic and post-pandemic periods would further strengthen the scientific validity and interpretability of the findings.

Response: We sincerely appreciate the Reviewer's detailed and constructive evaluation of our manuscript. The recommendations have substantially improved its scientific rigor, methodological transparency, and overall clarity. In response, we have revised the statistical presentation, strengthened the discussion of limitations, moderated causal interpretations, clarified the methodology, updated the literature review, and improved the overall presentation of the manuscript. We believe these revisions have addressed the Reviewer's concerns and have significantly strengthened the manuscript. Details of the individual revisions are provided in our responses above.

Reviewer 2’s comments

1. **The topic is relevant, especially for tropical LMIC settings where long-term influenza surveillance data are limited. The study provides clinically useful observations and adds regional epidemiological evidence. However, several methodological, statistical, and reporting issues need substantial clarification before the acceptance of the manuscript**

Response: We sincerely appreciate the Reviewer’s positive assessment of our topic's relevance and their recognition of the study’s clinical and regional epidemiological value for tropical low- and middle-income country (LMIC) settings.

We completely agree that robust methodology, precise statistical reporting, and absolute clarity are essential for the integrity of this long-term dataset. In this revised submission, we have carefully and systematically addressed every methodological, statistical, and reporting concern raised. We believe these refinements have substantially strengthened the rigor of our manuscript, ensuring it aligns fully with the high standards of the journal.

2. **The mortality model includes many covariates despite only 12–13 deaths. As number of deaths is low, the number of variables in the mortality model can be reduced.**

Response: We thank the Reviewer for this important statistical observation. We acknowledge that the limited number of deaths (n = 13) restricts the precision of the mortality model and may increase the risk of overfitting. As a similar concern was raised during the peer review process, we carefully revisited the mortality model and strengthened both its description and interpretation in the revised manuscript. Variable selection followed a pre-defined purposeful selection strategy based on both statistical criteria (P < 0.25 in the univariable analysis) and clinical relevance, as recommended in the methodological literature. We retained clinically important variables to reduce potential confounding, but we also recognised that the limited number of events resulted in wide confidence intervals for several estimates. We used Firth logistic regression to reduce small sample bias and improve the stability of parameter estimation in the presence of sparse mortality events. Additionally, we have included model diagnostics appropriate for each multivariable model. For the ICU admission model, calibration was assessed using the Hosmer–Lemeshow goodness-of-fit test, which showed no evidence of poor model fit. For the mortality model, which was analysed using Firth's penalized logistic regression, model performance was evaluated using the penalized likelihood ratio test together with the area under the receiver operating characteristic curve (AUC). For the mortality model, multicollinearity was assessed using variance inflation factors (VIFs), and all VIF values were below 5.0, indicating no evidence of problematic multicollinearity. Model goodness-of-fit and discrimination were assessed using the penalized likelihood ratio test and the area under the curve (AUC / C-statistic) which was 0.9515. The overall mortality model demonstrated good performance, as evidenced by the penalized likelihood ratio test (chi-square = 42.575, df = 16, P < 0.001) together with excellent discrimination (AUC = 0.9515), although the model should still be interpreted cautiously because of the limited number of mortality events. Despite these strategies, we acknowledge that the number of covariates remains high relative to the number of events and therefore regard the mortality model as exploratory. In response to this, we further strengthened the Limitations section to explicitly acknowledge the exploratory nature of the mortality model and the possibility of model instability.

Statistical analysis

"…. A standard multivariable logistic regression for ICU admission prediction model was constructed. Model calibration and goodness-of-fit were formally evaluated using the Hosmer–Lemeshow test, while discrimination capacity was assessed via the Area Under the ROC Curve (AUC / C-statistic). Multicollinearity among predictors was monitored using Variance Inflation Factors (VIF). For mortality model, due to low-event constraints (sparse mortality data), Firth's penalized logistic regression was employed to minimize small-sample bias and avoid issues of separation. Complete-case analysis was applied to this model. Goodness-of-fit was evaluated using the Penalized Likelihood Ratio Test, and discrimination power was determined via the AUC / C-statistic. Multicollinearity was similarly assessed using VIF. Given the potential for confounding among covariates, the purposeful selection process was used to identify covariates for the regression models. Following the methodology described elsewhere (32, 33), a more generous P-value cutoff of <0.25 in the univariate analysis was used. This approach was chosen because traditional cutoffs (such as 0.05) often fail to identify variables that, while not independently significant, act as important confounders or become significant when adjusted for other covariates. Furthermore, variables considered clinically important based on the authors' expertise were retained irrespective of their univariable statistical significance to ensure that potential confounding was appropriately addressed…."

Result

Table 6. Logistic regression analysis of predictors of mortality among 158 patients with laboratory-confirmed A/(H1N1)pdm09 pneumonia

Variables	P value	AOR (95%CI)
Above 60 years old	0.205	5.137 (0.408-98.998)
Male	0.976	1.030 (0.146-8.282)
Comorbidities	0.731	1.445 (0.181-14.109)
Days from onset to admission	0.123	1.169 (0.918-1.388)
WBC (per mm^3)	0.187	0.848 (0.631-1.068)
Lymphocyte (per mm^3)	0.647	1.121 (0.080-1.810)
Serum creatinine (μmol/L)	0.425	1.011 (0.985-1.041)
AST (U/L)	0.330	1.006 (0.994-1.017)
ALT (U/L)	0.875	0.999(0.986-1.012)

Moderate-to-severe ARDS	0.927	0.885 (0.053-12.097)
Invasive ventilation	0.039	8.426 (1.105-141.366)
Double dose oseltamivir (150mg/12h) or combination*	0.020	8.577 (1.416-83.341)
Corticosteroids	0.286	0.371 (0.045-2.218)
Vasopressors	0.796	1.270 (0.189-7.600)
Hospital acquired infection	0.217	0.262 (0.024-2.151)
ICU admission	0.336	3.661 (0.248-81/225)

AOR: Adjusted odds ratio; ARDS: acute respiratory distress syndrome. Chi-square = 42.575, df = 16, $P < 0.001 (Penalized likelihood ratio test). Multicollinearity assessment via Variance Inflation Factors (VIF) indicated no problematic collinearity (all VIFs < 5.0, ranging from 1.087 to 3.625; highest for AST and ALT). The Area Under the ROC Curve (AUC / C-statistic) was 0.9515.

*Combination: Oseltamivir with Zanamivir or Ribavirin

3. **The manuscript concludes that double-dose oseltamivir or combination therapy is associated with mortality. Rephrase consistently as: "associated with" or "observed among"**

Response: We agree with the Reviewer that the association of boosted antiviral therapy in our study with mortality should be interpreted more carefully because of the limitation of evidence. Therefore, we would like to change as

Discussion – 6th paragraph

"…The observed association between intensified antiviral therapy and mortality most likely reflects confounding by indication. Patients with more severe disease were preferentially treated with intensified antiviral regimens rather than a direct harmful effect of the therapy itself. Current evidence does not demonstrate a clear survival benefit from intensified antiviral regimens in severe influenza, and prospective studies are needed before definitive conclusions regarding their clinical effectiveness can be drawn…"

4. **Avoid treatment recommendations unsupported by observational data**

Response: We sincerely thank the Reviewer for this insightful recommendation. We agree that our initial conclusion regarding antiviral therapy was overstated given the retrospective nature

of our study. As suggested, we have removed the treatment recommendation regarding intensified antiviral therapy and replaced it with a more cautious interpretation that is consistent with the observational design of our study. The discussion and conclusion sections have been revised as follows:

Discussion – 6th paragraph

" …The observed association between intensified antiviral therapy and mortality most likely reflects confounding by indication. Patients with more severe disease were preferentially treated with intensified antiviral regimens rather than a direct harmful effect of the therapy itself. Current evidence does not demonstrate a clear survival benefit from intensified antiviral regimens in severe influenza, and prospective studies are needed before definitive conclusions regarding their clinical effectiveness can be drawn.

Conclusion

The study did not demonstrate improved outcomes with intensified antiviral regimens; however, prospective studies are required before definitive treatment recommendations can be made.

5. **Avoid concluding that alternative antiviral regimens "should not be recommended"**

Response: We sincerely thank the Reviewer for this insightful recommendation. We agree that our observational study does not support definitive treatment recommendations regarding intensified antiviral regimens. Accordingly, we have removed the previous recommendation against these regimens and revised the Conclusion to present a more cautious interpretation that is consistent with the study design. The revised Conclusion now reads:

Conclusion

"…The study did not demonstrate improved outcomes with intensified antiviral regimens; however, prospective studies are required before definitive treatment recommendations can be made."

6. **ICU Admission Criteria not defined whether uniformly applied during the study period because ICU admission is one of the primary outcomes.**

Response: We thank the Reviewer for highlighting these potential sources of temporal bias, which are critical to address in a long-term cohort study. We have clarified in the manuscript that the core clinical criteria for ICU admission (severe ARDS, septic shock, or neurological deterioration) remained consistent throughout the 10-year study period. Furthermore, as the

primary regional referral center for infectious diseases, our hospital maintained dedicated capacity and prioritized bed availability for severe influenza cases, minimizing admission bias driven by changing structural resources. We have amended the Materials and Methods section:

Materials and Methods section

"…Indications for ICU admission were in accordance with the national guidelines for diagnosis and treatment of influenza [30] and international recommendations [10, 31] including development of respiratory distress or septic shock. These clinical admission criteria and thresholds remained consistent throughout the 10-year study period, and admission decisions were not constrained by ICU bed availability…."

7. **Given the retrospective design over 10 years, missing data are expected and whether it affected the analysis on variables.**

Response: We thank the Reviewer for this important comment. We agree that missing data are an inherent limitation of long-term retrospective studies. Primary and secondary outcome data were complete for all enrolled patients. Predictor variables with substantial missing data were excluded from the multivariable regression analyses, as described in the Statistical Analysis section. For descriptive analyses, percentages were calculated using the available data for each variable, and the corresponding denominators are reported where appropriate. We have clarified our approach to missing data in the Statistical Analysis section and further acknowledged its potential influence in the Limitations section, as follows:

Discussion – 7th paragraph

"…Our study has some notable limitations. Firstly, this study was based on an analysis of medical records and thus, data such as patients' BMI (56) that may confound the study findings have not been documented. Additionally, some predictor variables contained missing data. Variables with substantial missingness were excluded from the multivariable analyses, which may have limited our ability to evaluate the effects of some potentially relevant predictors…."

8. **Establish interpretation of ALT & AST biomarkers more cautiously and also its association with mortality if any.**

Response: We thank the Reviewer for this important observation. We agree that the interpretation of AST and ALT elevations should be made cautiously, particularly regarding their relationship with mortality. Although elevated AST and ALT levels were associated with ICU admission, they were not independently associated with mortality in the multivariable

analysis. Therefore, we have revised the Discussion to interpret these biomarkers as indicators of disease severity rather than independent prognostic factors for mortality. Our revision is as follows.

Discussion section – 5th paragraph

"…We similarly observed an association between ALT, AST levels and ICU admission among our participants. elevated AST and ALT levels may reflect hepatic involvement associated with more severe systemic illness rather than representing independent predictors of mortality (51). …"

9. **Data on secondary bacterial Co- Infection not mentioned as this strongly influences influenza mortality.**

Response: We thank the Reviewer for this important observation. We agree that secondary bacterial co infection is an important contributor to disease severity and mortality in patients with influenza. However, because this was a retrospective study based on routine clinical records, microbiological investigations were performed only when clinically indicated rather than systematically for all patients. Consequently, microbiologically confirmed bacterial co infection at hospital admission could not be evaluated as a separate study variable.

Instead, our study recorded hospital acquired infections that developed during hospitalization according to predefined clinical and microbiological criteria. We have clarified this distinction in the Methods section and acknowledged it as a limitation of the study.

Among patients investigated for suspected secondary infection, microbiological confirmation was obtained in 10 cases. Given the small number of microbiologically confirmed bacterial or fungal infections, separate statistical analyses would not have been reliable and were therefore not performed. Our revision is as follows.

Discussion section – limitations paragraph:

"…Systematic microbiological investigations for bacterial co-infection at hospital admission were not performed for all patients. Therefore, the potential contribution of community acquired bacterial co infection to disease severity and mortality could not be evaluated in this study."

10. **Vaccination Data to be included as the discussion repeatedly attributes declining case numbers to vaccination and herd immunity.**

Response: We thank the Reviewer for this important observation. Given that this was a retrospective study utilizing paper-based medical records from a period when routine influenza vaccination was not widely structured or systematically tracked in the general Vietnamese community, these historical data were unfortunately unavailable in the patient charts. We have recognized this as a major limitation and have explicitly revised the Limitations section to highlight its potential confounding impact:

Discussion section – limitations paragraph:

"…Additionally, some predictor variables contained missing data. Variables with substantial missingness were excluded from the multivariable analyses, which may have limited our ability to evaluate the effects of some potentially relevant predictors. Importantly, individual influenza vaccination status was not recorded in the historical medical records. Since vaccination can significantly reduce clinical severity, its absence may have confounded the observed associations with ICU admission, disease severity, and mortality…"

11. **Language and Grammar: The manuscript requires substantial English editing.**

Examples: "the mortality due to A/(H1N1)pdm09-related pneumonia is not rare"

→ better: "mortality remains substantial" .Inconsistent use of:

- **A(H1N1)pdm09**
- **A/(H1N1)pdm09**

Response: We sincerely thank the Reviewer for pointing out these language and grammatical inconsistencies. We agree that precise nomenclature and formal phrasing are vital for the clarity and professionalism of the manuscript. We apologize for the inconsistent notation of the virus strain. We have audited the entire manuscript and standardized all references uniformly to "A(H1N1)pdm09" as per standard global surveillance conventions, removing all instances of the slashed variant ("A/(H1N1)pdm09"). In addition, we have adopted your excellent suggestion and replaced the awkward phrasing "the mortality due to A/(H1N1)pdm09-related pneumonia is not rare" with the much more precise and objective clinical phrasing: "mortality remains substantial."

Discussion and Conclusion

Despite effective treatment and vaccines, our study confirms that the mortality due to A(H1N1)pdm09-related pneumonia remains substantial.

12. **Several typographical and formatting errors needs corrections:**

- **Line 84 consists of some irrelevant data, spacing consistencies, inconsistent confidence interval formatting, inconsistent decimal formatting**

- **Mortality Count Inconsistency: The abstract reports 13 deaths. However, Table 4 mortality analysis uses n=12. Is this data incomplete or missing?**

Response: The Reviewer is entirely correct. The absolute number of deaths in this cohort is 13 (resulting in a mortality rate of 8.2%). The value of 12 previously listed in Table 4 was a typographical error. We have corrected this cell in Table 4, updated the associated percentages in that column, and re-verified that all tables (including Table 6) and prose throughout the Abstract and Results sections uniformly report 13 deaths. Besides, we apologize for the grammar, spacing, and formatting artifacts left in the previous draft, which have now been completely removed.

13. **Pregnancy outcomes needs to be mentioned by provision of age, complications etc**

Response: We thank the Reviewer for highlighting the importance of pregnancy-specific data. We agree that characterizing this high-risk sub-group adds important clinical context to our findings. Given that pregnancy represented an important predefined subgroup, we have added a brief summary of the pregnancy outcomes to the Results section. We note, however, that due to the retrospective nature of this chart review, data regarding specific neonatal parameters and long-term post-discharge complications were not captured in the medical records.

Result

Among the 26 pregnant patients included, 57.7% required ICU admission and three (11.5%) died. Four women went into labor while being treated for influenza, and one case resulted in stillbirth (data not shown).

14. **Figures: Figures 1 and 2 are referenced but not adequately described in text.**

Flow diagram needs cases that were excluded and missing data

Response: We thank the Reviewer for this important structural critique. We agree that a transparent study flow is vital for establishing the robustness of our cohort. To clarify, no recruited patients were excluded after meeting the study criteria. All patients diagnosed with A(H1N1)pdm09 infection during the study period who met our baseline entry criteria were included. We have ensured that there were no missing data for the primary and secondary study outcomes. Figure 1 has been revised to indicate that no eligible patients were excluded

after application of the study eligibility criteria. Additionally, we have updated Figure 2 to display the annual distribution of hospitalized cases, ICU admissions, and deaths throughout the study period.

Results

Baseline characteristics of study participants

A total of 729 A(H1N1)pdm09 infected patients received treatment at the HTD during the study period with no subsequent exclusions or missing data for primary outcomes (Figure 1). Most (73%, 532/729) patients were hospitalized in 2009 (Figure 2). Of these 729 patients, A(H1N1)pdm09-related pneumonia was diagnosed in 21.7% (158/729, 95% CI 18.8-24.8%).

15. **Clarification on combination therapy includes: oseltamivir with zanamivir or ribavirin, consider reporting them separately if sample size permits**

Response: We really appreciate your feedback. In our cohort, 14 patients received other antiviral regimens. In more details, the other antiviral regimens included ribavirin (n=2), zanamivir (n=12). Given that the numbers in each subgroup were very small, separate statistical analyses would not have been meaningful. To improve transparency, we have clarified the composition of the combination therapy group in a table footnote as follows:

. **Combination therapy: oseltamivir with zanamivir (n=12) or ribavirin (n=2).

16. **STROBE Initiative guidelines can be applied for observational studies**

Response: We thank the Reviewer for this valuable recommendation. We agree that the STROBE Statement provides an important framework for improving the transparency and completeness of reporting in observational studies. Accordingly, we have reviewed our manuscript against the STROBE recommendations. A completed STROBE checklist has also been provided as a supplementary file.

Methods section – the last paragraph:

Maintenance of study standard

This study was reported in line with the STROBE guideline for cohort studies (Appendix 1).

Reviewer 3's comments:

1. **Article fits well within scope of the journal. There are no ethical concerns with this article. The title is representative of the article's contents. The abstract**

summarises the contents clearly. The objectives are well stated. The methodology used is appropriate and well described. I am not an expert on statistics. The main claims of the paper on ICU admission and mortality in adult patients with influenza A/H1N1-related pneumonia in Vietnam is well elucidated and is significant in its observations on reducing mortality in its discipline by stressing on early diagnosis, use of vaccines and prevention of secondary bacterial infections and the use of vaccines in all ages.

Response: We sincerely thank the Reviewer for this highly encouraging evaluation. We are pleased that the core claims, clinical objectives, and methodological approach of our study were found to be clear, appropriate, and well-supported. Your validation of our findings—specifically regarding the emphasis on early diagnosis, preventive vaccine strategies across all ages, and the prevention of secondary bacterial infections to mitigate mortality—is deeply appreciated by our team.

2. **The results have been stated clearly and answers the research question. The discussion is appropriate and the study limitations have been well alluded to. The conclusions are justified based on the data presented. The references are relevant and recent.**

Response: We are very grateful for the Reviewer's positive assessment of our Results, Discussion, and Conclusions sections. We appreciate your recognition that our conclusions are justified by the presented data and that our study limitations were transparently addressed. Thank you also for validating the relevance and contemporary nature of our literature references.

3. **The manuscript is well organized and written clearly enough to be accessible to non-specialists.**

Response: We sincerely thank the Reviewer for this encouraging assessment. We are pleased that the organization and clarity of the manuscript were considered accessible to both specialists and non-specialists. We have continued to refine the manuscript throughout this revision to further improve its clarity and readability.

4. **The article is deemed suitable for publication.**

Response: We sincerely thank the Reviewer for recommending our manuscript for publication. Your positive evaluation and constructive validation strongly encourage our team to continue advancing our clinical research in infectious diseases and critical care.
