# Supplementary material for: ICU admission and mortality in adult patients with influenza A(H1N1)pdm09-related pneumonia in Vietnam since the 2009 H1N1 pandemic: A 10-year cohort study
Source: PLoS One. 2026 Aug 24;21(8):e0348450. doi: 10.1371/journal.pone.0348450 (PMC13502601; doi:10.1371/journal.pone.0348450)
Supplement: Supplementary file 2 [file pone.0348450.s001.docx]

# **Peer Review Report**

## **Manuscript Title**

Burden of ICU Admission, Mortality, and Predictors among Patients with A(H1N1)pdm09 Pneumonia in Vietnam (2009–2019)

# **Overall Evaluation**

This manuscript presents a 10-year retrospective cohort study evaluating ICU admission, mortality, and predictors of severe outcomes among adults with A(H1N1)pdm09 pneumonia in Vietnam. The study addresses an important public health issue, particularly in tropical and resource-limited settings where long-term influenza surveillance data are limited. The long study duration and inclusion of real-world clinical data are notable strengths.

The manuscript is clinically relevant and provides useful epidemiological and outcome data from a tropical Asian setting. However, several methodological, statistical, and presentation-related issues should be addressed before publication. Some conclusions are currently overstated relative to the observational nature of the study and the limited sample size, especially regarding antiviral therapy and mortality.

Overall recommendation: **Major Revision**

# **Major Comments**

## 1. Clarify Study Objective and Primary Outcomes

The objectives should be more explicitly stated in the Introduction. Currently, the manuscript discusses ICU admission, mortality, and treatment outcomes, but the primary and secondary outcomes are not clearly defined.

### Suggested improvement:

At the end of the Introduction, clearly state:

- Primary outcomes: ICU admission and mortality
- Secondary outcomes: ventilation requirement, ARDS, hospital-acquired infection, and length of stay

## 2. Concern Regarding Small Number of Mortality Events

The mortality analysis appears statistically underpowered.

Only 13 deaths occurred, yet many variables were entered into the multivariable logistic regression model. This raises concern for:

- Model overfitting
- Unstable estimates
- Extremely wide confidence intervals
- Reduced reliability of adjusted odds ratios

Examples:

- Invasive ventilation AOR 55.355 (95% CI 1.486–2062.375)
- Double-dose oseltamivir/combination therapy AOR 32.625 (95% CI 1.594–667.661)

These wide confidence intervals indicate substantial statistical instability.

### Suggestions:

- Reduce the number of variables included in the mortality regression model.
- Consider using penalized logistic regression or Firth logistic regression.
- Include an events-per-variable justification.
- Acknowledge possible model instability more explicitly in limitations.

## 3. Interpretation of Antiviral Therapy Findings is Overstated

The manuscript repeatedly implies that double-dose oseltamivir or combination therapy may increase mortality. However, this conclusion is not sufficiently supported because:

- This is an observational retrospective study.
- Sicker patients are more likely to receive aggressive therapy (confounding by indication).
- Disease severity itself likely influenced treatment decisions.

Therefore, the association should not be interpreted causally.

### Current problematic statements:

- “Neither double-dose oseltamivir nor combination therapy is recommended…”
- “Alternative antiviral regimens should not be recommended.”

These conclusions exceed the evidence generated by this study.

### Suggested revision:

Replace with more cautious wording such as:

“The observed association between intensified antiviral therapy and mortality likely reflects treatment allocation to more severely ill patients rather than a direct harmful effect of therapy.”

And:

“Further prospective studies are required to evaluate the efficacy of intensified antiviral regimens in severe influenza.”

## 4. ICU Admission Criteria Need Better Standardization

The manuscript states that ICU admission followed national and international guidelines. However, ICU admission practices can vary over time, physicians, and hospital capacity.

Given the 10-year study duration, ICU admission thresholds may have changed.

### Suggestions:

- Clarify whether ICU criteria remained consistent throughout the study period.
- Mention whether ICU bed availability influenced admission decisions.
- Discuss possible temporal bias.

## 5. Temporal Changes Across 2009–2019 Were Not Analyzed

More than 70% of cases occurred in 2009, yet the study combines all years together.

Clinical management, vaccination uptake, antiviral use, and ICU care likely evolved over time.

### Suggestions:

- Include subgroup analysis comparing:
  - Pandemic period (2009–2010)
  - Post-pandemic years
- Assess whether mortality or ICU admission differed by time period.
- Alternatively, discuss this limitation more thoroughly.

## 6. ARDS and ICU Admission Relationship May Introduce Circular Reasoning

Moderate-to-severe ARDS is identified as a predictor of ICU admission, but ARDS itself is often one of the indications for ICU transfer.

Thus, the relationship may partly reflect clinical decision-making rather than independent prediction.

### Suggestions:

- Clarify timing of ARDS diagnosis relative to ICU admission.
- Discuss possible incorporation bias.

## 7. Lack of Vaccination Status is a Significant Limitation

Influenza vaccination status is highly relevant when studying severity and mortality.

The absence of vaccination data should be highlighted more strongly because it may substantially confound:

- ICU admission
- Disease severity
- Mortality

### Suggestion:

Explicitly discuss this as a major limitation.

## 8. Potential Selection Bias

The Hospital for Tropical Diseases is a referral center, which likely receives more severe cases.

Therefore:

- Mortality estimates may not represent the general population.
- ICU admission rates may be higher than in community hospitals.

### Suggestion:

State this limitation clearly in Discussion.

## 9. Clarify Definitions of Pneumonia and Secondary Infection

The manuscript defines pneumonia using chest radiography findings, but additional clarification is needed.

### Suggestions:

Clarify:

- Whether radiographs were independently reviewed
- Whether bacterial co-infection at admission was assessed
- Diagnostic criteria for hospital-acquired infection
- Whether microbiological confirmation was available

## 10. Statistical Presentation Needs Improvement

Several odds ratios are reported as 1.000 (1.000–1.000), which is not informative.

### Suggestions:

- Rescale laboratory variables (e.g., per 1000 cells/mm3 or per 10-unit increase).
- Present clinically meaningful increments.
- Provide model diagnostics:
  - Hosmer–Lemeshow test
  - Multicollinearity assessment
  - ROC curve/AUC if available

# **Minor Comments**

## Abstract

### Comments:

1. The abstract is too dense and numerically overloaded.
2. Confidence intervals should be formatted consistently.
3. Avoid causal wording regarding antiviral therapy.

### Suggested wording:

Instead of: > “Predictors of mortality were invasive ventilation and double-dose oseltamivir…”

Use: > “Mortality was associated with invasive ventilation and intensified antiviral therapy.”

## Introduction

### Comments:

1. Several sentences are excessively long.
2. Grammar and spacing need improvement.
3. Some references are outdated or repetitive.

### Specific corrections:

- “death associated with influenza” → “influenza-associated mortality”
- “death, suggesting age may play” → add clearer transition
- Remove formatting artifacts:
  - “[MH1.1][MH1.2][DBT(1.3]”

## Materials and Methods

### Comments:

1. Clarify whether consecutive sampling was used.
2. Explain how missing data were handled.
3. State whether all laboratory tests were performed at admission.
4. Mention whether coinfections were assessed.
5. Clarify why patients aged 17 years were classified as adults.

### Important:

The methodology for purposeful variable selection should be described more concisely.

## Results

### Comments:

1. Ensure consistency in percentages and decimal places.
2. Tables should be simplified.
3. Some variables are repeated unnecessarily.
4. Clarify discrepancy:
   - Mortality reported as 13 in some sections and 12 in Table 4.

This inconsistency must be corrected.

## Discussion

### Comments:

1. Some paragraphs are overly descriptive.
2. Discussion should focus more on interpretation rather than repeating results.
3. Comparison with previous literature should be more balanced.
4. Avoid overstating vaccine effects because vaccination status was unavailable.

### Important conceptual issue:

The discussion frequently implies causality from observational associations.

Please use more cautious language such as:

- “associated with”
- “may reflect”
- “possibly related to”

instead of:

- “caused”
- “increased risk due to”
- “should not be recommended”

## Conclusion

The conclusion is generally appropriate but currently too strong regarding antiviral therapy.

### Suggested revision:

Replace: > “Neither double-dose oseltamivir nor combination therapy is recommended…”

With: > “The study did not demonstrate improved outcomes with intensified antiviral regimens; however, prospective studies are required before definitive treatment recommendations can be made.”

# Language and Formatting Corrections

The manuscript requires careful English editing.

Examples:

| Current Text | Suggested Revision |
| --- | --- |
| “death associated with influenza” | “influenza-associated mortality” |
| “risk factors of A/(H1N1)pdm09 infection-related ICU admission” | “risk factors for ICU admission related to A(H1N1)pdm09 infection” |
| “patients demanded respiratory support” | “patients required respiratory support” |
| “liver affection” | “hepatic involvement” |
| “timely detection of complications requiring intensive care” | “early identification of complications requiring intensive care” |

# Strengths of the Study

1. Long study duration (10 years)
2. Valuable tropical-region influenza data
3. Real-world clinical dataset
4. Important information regarding ICU burden and ARDS
5. Inclusion of laboratory and treatment variables
6. Relevant topic for infectious disease and critical care literature

# Reference Review and Citation Assessment

## Overall Assessment of References

The manuscript includes a broad range of references from WHO, CDC, peer-reviewed journals, and regional influenza studies. Most references are relevant and support the general scientific background appropriately. However, several citations are either:

- Weakly connected to the statement they support
- Overinterpreted
- Outdated when newer evidence exists
- Not optimally matched to the conclusions being drawn

In multiple sections, the discussion extrapolates beyond what the cited literature directly demonstrates.

## Detailed Reference-to-Text Evaluation

### 1. Mortality and Global Burden Statements

#### Text:

“The A(H1N1)pdm09 virus remains a major global health threat.”

### References Used:

- WHO (Ref 1)
- Simonsen et al. (Ref 2)
- CDC (Ref 3)

### Review:

These references appropriately support pandemic burden and historical mortality. However, the wording “major global health threat” may be slightly overstated in the current post-pandemic era unless supported with more recent burden data.

### Suggestion:

Include newer global influenza burden references (2022–2025), especially post-COVID influenza resurgence studies.

### 2. Tropical Influenza Seasonality

#### Text:

“Influenza circulation patterns in tropical areas are often irregular…”

### References Used:

- Muscatello et al. (Ref 5)
- Vietnam surveillance study (Ref 21)

### Review:

These references are appropriate and well-matched.

Strong citation alignment.

### 3. Risk Factors for Severe Disease

#### Text:

“High-risk groups include children younger than five years, the elderly, pregnant women…”

### References Used:

- Ref 8
- Ref 9
- Ref 10

### Review:

Ref 8 (Miyazawa) mainly discusses overlap between COVID-19 and H1N1 metabolic risk factors and is not the strongest citation for classical influenza high-risk groups.

### Better references:

- WHO influenza risk group guidance
- CDC influenza high-risk population guidance
- Mertz et al. systematic review (already included as Ref 14)

### Recommendation:

Replace Ref 8 here with stronger epidemiological guidance references.

### 4. Pregnancy and Severity

#### Text:

“Pregnant women are prioritized for influenza vaccination.”

### References Used:

- WHO vaccination guidance (Ref 13)

### Review:

Appropriate and well-supported.

However:

#### Text:

“Mexican study found reduced fatality rate among pregnant women…”

### Reference:

- Chowell et al. (Ref 11)

### Concern:

The manuscript uses this finding to suggest variability in severity associations across countries. While technically true, this statement requires more nuanced interpretation because:

- Pregnancy is still globally accepted as a high-risk condition.
- The Mexican findings may reflect healthcare access, earlier hospitalization, or surveillance differences.

### Suggestion:

Avoid creating doubt regarding pregnancy risk status.

Suggested wording:

“Although some studies reported differing mortality estimates among pregnant women, pregnancy remains an established high-risk condition for severe influenza outcomes.”

### 5. Surveillance Challenges in Tropical Regions

### References:

- Refs 16–20

### Review:

Excellent and well-integrated references.

These strongly support the rationale for the study.

### 6. Vaccination Discussion

#### Text:

“Decrease in inpatient cases over time is probably attributable to vaccination and herd immunity.”

### References:

- Refs 34–36

### Major Concern:

This conclusion is speculative.

The cited references discuss vaccine uptake and influenza training but do NOT directly demonstrate causality between vaccination and reduced admissions in this cohort.

Additionally:

- Vaccination status was unavailable.
- No population-level vaccination coverage trend analysis was performed.

### Recommendation:

Tone down interpretation.

Suggested revision:

“The reduction in hospitalized cases over time may partly reflect increasing population immunity, including possible contributions from vaccination and prior exposure.”

### 7. Radiological Findings Compared with COVID-19

### References:

- Ref 39
- Ref 40

### Review:

Appropriate and relevant.

The discussion is balanced and clinically meaningful.

### 8. Early Oseltamivir Therapy

#### Text:

“Early treatment with oseltamivir can reduce lower respiratory tract complications.”

### Reference:

- Moreno et al. (Ref 41)

### Concern:

Ref 41 specifically evaluated critically ill influenza pneumonia patients and survival benefit from early oseltamivir.

The manuscript slightly generalizes this to all adult influenza patients.

### Suggestion:

Clarify patient population.

### 9. International Mortality Comparisons

### References:

- India (Ref 42)
- Singapore (Ref 43)
- Vietnam (Ref 45)

### Review:

These comparisons are relevant.

However, mortality comparisons between countries should acknowledge:

- Different hospitalization thresholds
- ICU availability
- Diagnostic criteria
- Population structures
- Healthcare systems

### Suggestion:

Explicitly mention that direct comparison should be interpreted cautiously.

### 10. AST/ALT and Liver Involvement

### References:

- Ref 47

### Review:

Appropriate citation.

However, the phrase “liver affection” is non-standard English.

Use:

- “hepatic involvement”
- “influenza-associated hepatic injury”

### 11. Invasive Ventilation and Mortality

### References:

- Refs 48–51

### Review:

These references appropriately support the discussion.

However, the manuscript sometimes implies invasive ventilation itself causes mortality.

This is problematic because invasive ventilation is primarily a marker of disease severity.

### Recommendation:

Clarify:

“The association likely reflects underlying disease severity among patients requiring invasive respiratory support.”

### 12. Double-Dose Oseltamivir and Combination Therapy

### References:

- Refs 52–54

### Major Concern:

The manuscript selectively interprets these references.

The cited studies mainly conclude:

- No clear mortality benefit
- Limited evidence
- Confounding in observational studies

But the manuscript concludes: > “Neither double-dose oseltamivir nor combination therapy is recommended.”

This is stronger than the evidence.

### Recommendation:

The discussion should instead state:

“Current evidence does not demonstrate a clear survival benefit from intensified antiviral regimens in severe influenza.”

Avoid treatment recommendations beyond observational evidence.

### 13. Age and Mortality Discussion

### References:

- Ref 55
- Ref 56

### Review:

Appropriate use of literature.

The manuscript appropriately acknowledges heterogeneity across countries.

This section is scientifically balanced.

## Reference Formatting Issues

Several formatting inconsistencies were identified:

### Examples:

1. WHO references inconsistently formatted.
2. Some URLs are broken across lines.
3. “World Health Organisation” and “World Health Organization” used interchangeably.
4. Journal title capitalization inconsistent.
5. Some references missing DOI information.
6. Reference 23 is incomplete and poorly formatted.
7. Reference 36 appears mismatched:
   - “Training on Influenza Data Management and Basic Analysis: Maldives 2023” does not strongly support herd immunity/vaccination discussion.

### Recommendation:

The reference list should be professionally reformatted according to journal style.

## Additional Literature Recommended

The manuscript would benefit from citing:

1. Recent WHO influenza surveillance reports (2023–2025)
2. Post-COVID influenza epidemiology studies
3. Updated antiviral treatment guidelines
4. Studies on influenza-vaccination effectiveness in Southeast Asia
5. Modern ARDS management guidelines

## Most Scientifically Strong Sections

The following areas are well-supported by references:

- Influenza surveillance gaps in tropical regions
- ARDS definitions and ICU indications
- Radiological overlap with COVID-19
- Mechanical ventilation discussions
- Regional mortality comparisons

## Sections Requiring Stronger Evidence or More Careful Interpretation

1. Vaccine-related reduction in hospitalizations
2. Interpretation of intensified antiviral therapy
3. Implication that invasive ventilation contributes directly to mortality
4. Pregnancy-related severity interpretation
5. Generalization of oseltamivir evidence

# **Final Recommendation**

## Recommendation: Major Revision

The manuscript addresses an important clinical and epidemiological topic and contains valuable long-term data from Vietnam. However, substantial revisions are required, particularly regarding:

- Statistical robustness
- Interpretation of antiviral therapy findings
- Clarification of methodology
- Reduction of causal language
- Improved discussion of limitations
- English language editing

With these revisions, the manuscript could make a meaningful contribution to influenza outcome research in tropical settings.

Additionally, although the long-term dataset is valuable, the study period (2009–2019) raises concerns regarding the contemporary applicability of the findings. A substantial proportion of cases were recorded during the 2009 pandemic phase, while influenza epidemiology, vaccination coverage, antiviral strategies, ICU management protocols, diagnostic capabilities, and supportive care practices have evolved considerably over time. Therefore, the generalizability of these findings to current clinical settings may be limited. The manuscript would benefit from a more detailed discussion on temporal heterogeneity and changes in clinical management across the study period. Stratified analyses comparing pandemic and post-pandemic periods would further strengthen the scientific validity and interpretability of the findings.
