# Supplementary material for: ICU admission and mortality in adult patients with influenza A(H1N1)pdm09-related pneumonia in Vietnam since the 2009 H1N1 pandemic: A 10-year cohort study
Source: PLoS One. 2026 Aug 24;21(8):e0348450. doi: 10.1371/journal.pone.0348450 (PMC13502601; doi:10.1371/journal.pone.0348450)
Supplement: Supplementary file 3 [file pone.0348450.s003.docx]

PONE-D-26-17842_

- Article fits well within scope of the journal. There are no ethical concerns with this article. The title is representative of the article’s contents. The abstract summarises the contents clearly. The objectives are well stated. The methodology used is appropriate and well described. I am not an expert on statistics. The main claims of the paper on ICU admission and mortality in adult patients with influenza A/H1N1-related pneumonia in Vietnam is well elucidated and is significant in its observations on reducing mortality in its discipline by stressing on early diagnosis, use of vaccines and prevention of secondary bacterial infections and the use of vaccines in all ages.
- The results have been stated clearly and answers the research question. The discussion is appropriate and the study limitations have been well alluded to. The conclusions are justified based on the data presented. The references are relevant and recent.
- T he manuscript is well organized and written clearly enough to be accessible to non-specialists.
- The article is deemed suitable for publication.
