# Supplementary material for: ICU admission and mortality in adult patients with influenza A(H1N1)pdm09-related pneumonia in Vietnam since the 2009 H1N1 pandemic: A 10-year cohort study
Source: PLoS One. 2026 Aug 24;21(8):e0348450. doi: 10.1371/journal.pone.0348450 (PMC13502601; doi:10.1371/journal.pone.0348450)
Supplement: Supplementary file 4 [file pone.0348450.s002.odt]

Reviewer comments
The topic is relevant, especially for tropical LMIC settings where long-term influenza surveillance data are limited. The study provides clinically useful observations and adds regional epidemiological evidence. However, several methodological, statistical, and reporting issues need substantial clarification before the acceptance of the manuscript 
Major Concerns
The mortality model includes many covariates despite only 12–13 deaths.  As number of deaths is low, the number of variables in the mortality model can be reduced. 
The manuscript concludes that double-dose oseltamivir or combination therapy is associated with mortality. Rephrase consistently as: “associated with” or “observed among” 
Avoid treatment recommendations unsupported by observational data 
Avoid concluding that alternative antiviral regimens “should not be recommended” 
ICU Admission Criteria not defined whether uniformly applied during the study period because ICU admission is one of the primary outcomes.
Given the retrospective design over 10 years, missing data are expected and whether it affected the  analysis on variables .
Establish interpretation of ALT & AST biomarkers more cautiously and also its association with mortality if any.
Data on secondary bacterial Co- Infection not mentioned as this  strongly influences influenza mortality.
Vaccination Data to be included  as  the discussion repeatedly attributes declining case numbers to vaccination and herd immunity. 
Language and Grammar: The manuscript requires substantial English editing.
Examples: “the mortality due to A/(H1N1)pdm09-related pneumonia is not rare”
→ better: “mortality remains substantial” .Inconsistent use of: 
A(H1N1)pdm09 
A/(H1N1)pdm09 
Several typographical and formatting errors needs corrections:
Line 84 consists of some irrelevant data,  spacing consistencies, inconsistent confidence interval formatting , inconsistent decimal formatting 
Mortality Count Inconsistency: The abstract reports 13 deaths. However, Table 4 mortality analysis uses n=12. Is this data incomplete or missing?

Pregnancy outcomes needs to be mentioned by provision of age, complications etc
Figures: Figures 1 and 2 are referenced but not adequately described in text.
Flow diagram needs cases that were excluded and missing data 
Clarification on combination therapy includes: oseltamivir with  zanamivir or ribavirin, consider reporting them separately if sample size permits.
STROBE Initiative guidelines can be applied for observational studies
