# Supplementary material for: ICU admission and mortality in adult patients with influenza A(H1N1)pdm09-related pneumonia in Vietnam since the 2009 H1N1 pandemic: A 10-year cohort study
Source: PLoS One. 2026 Aug 24;21(8):e0348450. doi: 10.1371/journal.pone.0348450 (PMC13502601; doi:10.1371/journal.pone.0348450)
Supplement: Peer review history

# Peer review: Decision Letter 0

**DOI:** 10.1371/journal.pone.0348450.r001

**Authors:** Benjamin M. Liu

1 Jun 2026

Dear Dr. Bui,

**Academic Editor's Comments:**

Lines 129-137 "Nasal and oropharyngeal swabs were collected ... All laboratory tests were performed at the standardized Laboratory Department (ISO 15189) of the HTD." The methods section describes influenza A and influenza B detection using PCR, but it does not provide influenza A subtype (typing) information. In addition, the authors should introduce that LAMP has been successfully used for the detection of influenza A virus. More references should be cited, with this one (Liu, B.M. Isothermal nucleic acid amplification technologies and CRISPR-Cas based nucleic acid detection strategies for infectious disease diagnostics. p 30-47. In Manual of Molecular Microbiology: Fundamentals and Applications; ASM Press: Washington, DC, USA, 2025.doi:10.1128/9781683674597.ch03.) as an example (citing is optional).

==============================

Please submit your revised manuscript by Jul 16 2026 11:59PM. If you will need more time than this to complete your revisions, please reply to this message or contact the journal office at plosone@plos.org. When you're ready to submit your revision, log on to https://www.editorialmanager.com/pone/ and select the 'Submissions Needing Revision' folder to locate your manuscript file.

- A letter that responds to each point raised by the academic editor and reviewer(s). You should upload this letter as a separate file labeled 'Response to Reviewers'.
- A marked-up copy of your manuscript that highlights changes made to the original version. You should upload this as a separate file labeled 'Revised Manuscript with Track Changes'.
- An unmarked version of your revised paper without tracked changes. You should upload this as a separate file labeled 'Manuscript'.

If you would like to make changes to your financial disclosure, please include your updated statement in your cover letter. Guidelines for resubmitting your figure files are available below the reviewer comments at the end of this letter.

If applicable, we recommend that you deposit your laboratory protocols in protocols.io to enhance the reproducibility of your results. Protocols.io assigns your protocol its own identifier (DOI) so that it can be cited independently in the future. For instructions see: https://journals.plos.org/plosone/s/submission-guidelines#loc-laboratory-protocols. Additionally, PLOS ONE offers an option for publishing peer-reviewed Lab Protocol articles, which describe protocols hosted on protocols.io. Read more information on sharing protocols at https://plos.org/protocols?utm_medium=editorial-email&utm_source=authorletters&utm_campaign=protocols.

As the corresponding author, your ORCID iD is verified in the submission system and will appear in the published article. PLOS supports the use of ORCID, and we encourage all coauthors to register for an ORCID iD and use it as well. Please encourage your coauthors to verify their ORCID iD within the submission system before final acceptance, as unverified ORCID iDs will not appear in the published article. *Only*  the individual author can complete the verification step; PLOS staff *cannot*  verify ORCID iDs on behalf of authors.

We look forward to receiving your revised manuscript.

Kind regards,

Benjamin M. Liu, PhD, D(ABMM), MB(ASCP)

Academic Editor

PLOS One

**Journal Requirements:**

1. When submitting your revision, we need you to address these additional requirements.

Please ensure that your manuscript meets PLOS ONE's style requirements, including those for file naming. The PLOS ONE style templates can be found at

https://journals.plos.org/plosone/s/file?id=wjVg/PLOSOne_formatting_sample_main_body.pdf and

https://journals.plos.org/plosone/s/file?id=ba62/PLOSOne_formatting_sample_title_authors_affiliations.pdf

2. We note that you have included the phrase “data not shown” in your manuscript. Unfortunately, this does not meet our data sharing requirements. PLOS does not permit references to inaccessible data. We require that authors provide all relevant data within the paper, Supporting Information files, or in an acceptable, public repository. Please add a citation to support this phrase or upload the data that corresponds with these findings to a stable repository (such as Figshare or Dryad) and provide and URLs, DOIs, or accession numbers that may be used to access these data. Or, if the data are not a core part of the research being presented in your study, we ask that you remove the phrase that refers to these data.

3. Your ethics statement should only appear in the Methods section of your manuscript. If your ethics statement is written in any section besides the Methods, please move it to the Methods section and delete it from any other section. Please ensure that your ethics statement is included in your manuscript, as the ethics statement entered into the online submission form will not be published alongside your manuscript.

4. If the reviewer comments include a recommendation to cite specific previously published works, please review and evaluate these publications to determine whether they are relevant and should be cited. There is no requirement to cite these works unless the editor has indicated otherwise. 

[Note: HTML markup is below. Please do not edit.]

Reviewers' comments:

**Comments to the Author**

1. Is the manuscript technically sound, and do the data support the conclusions?

Reviewer #1: Yes

Reviewer #2: Yes

Reviewer #3: Yes

**********

2. Has the statistical analysis been performed appropriately and rigorously?

Reviewer #1: Yes

Reviewer #2: Yes

Reviewer #3: I Don't Know

**********

3. Have the authors made all data underlying the findings in their manuscript fully available?

The PLOS Data policy

Reviewer #1: No

Reviewer #2: Yes

Reviewer #3: Yes

**********

4. Is the manuscript presented in an intelligible fashion and written in standard English?

Reviewer #1: Yes

Reviewer #2: No

Reviewer #3: Yes

**********

**Reviewer #1:** This manuscript presents a 10-year retrospective cohort study evaluating ICU admission, mortality, and predictors among patients with A(H1N1)pdm09 pneumonia in Vietnam. The topic is clinically relevant and provides valuable long-term data from a tropical setting where such evidence is limited. The inclusion of real-world clinical data and focus on severe outcomes are notable strengths. However, several important issues need to be addressed before the manuscript can be considered for publication.

Major Comments

Clarification of Study Objectives

The study objectives should be more clearly defined in the Introduction. The distinction between primary outcomes (ICU admission, mortality) and secondary outcomes (e.g., ventilation requirement, ARDS, length of stay) should be explicitly stated.

Limited Mortality Events and Statistical Concerns

The mortality analysis appears underpowered, with only a small number of death events included in multivariable regression. This raises concerns about model overfitting and unstable estimates. The authors are encouraged to reduce the number of variables, justify events-per-variable, and consider alternative methods such as penalized regression. This limitation should also be clearly acknowledged.

Overinterpretation of Antiviral Therapy Findings

The manuscript suggests that intensified antiviral therapy (double-dose oseltamivir or combination therapy) may increase mortality. However, given the observational design, this likely reflects confounding by indication, where more severe patients received aggressive treatment. The conclusions should be revised to avoid causal interpretation and use cautious language such as “associated with” rather than “caused by.”

ICU Admission Criteria and Variability

The criteria for ICU admission need further clarification. Given the long study period, clinical practices and ICU availability may have varied. The authors should address whether criteria remained consistent and discuss potential variability.

Lack of Temporal Analysis

Most cases occurred during the 2009 pandemic period, yet data from all years are combined. Changes in clinical management over time may influence outcomes. A subgroup analysis comparing pandemic and post-pandemic periods, or at least a detailed discussion of this limitation, is recommended.

Potential Bias in ARDS and ICU Association

ARDS is identified as a predictor of ICU admission, but it is also a common indication for ICU transfer. This may introduce circular reasoning. The timing of ARDS diagnosis relative to ICU admission should be clarified.

Absence of Vaccination Data

Influenza vaccination status is not included, which is an important limitation. This should be emphasized more strongly, as it may significantly influence disease severity and outcomes.

Selection Bias

As the study was conducted in a referral hospital, more severe cases are likely overrepresented. This may limit generalizability and should be clearly discussed.

Definitions and Diagnostic Criteria

The manuscript would benefit from clearer definitions of pneumonia, ARDS, and hospital-acquired infections. Details regarding radiological assessment and microbiological confirmation should be added.

Statistical Presentation

Some results are difficult to interpret due to scaling issues (e.g., odds ratios of 1.000). Variables should be rescaled to clinically meaningful units, and model diagnostics should be included where possible.

Minor Comments

The abstract should be simplified and avoid overly dense numerical reporting.

Language and grammar require improvement throughout the manuscript.

Avoid causal wording; use terms such as “associated with” instead.

Ensure consistency in reported data (e.g., mortality counts across text and tables).

Tables can be simplified to improve readability.

Several references require updating, better alignment with statements, and formatting corrections.

Conclusion

The manuscript addresses an important topic and provides valuable long-term data. However, substantial revisions are required, particularly in methodology, statistical analysis, interpretation of findings, and language clarity. With these improvements, the study could make a meaningful contribution to the literature.

Recommendation: Major Revision

**Reviewer #2:**  The ethics approval for conduct of this retrospective study was received but doesn`t specify if the informed consent was waived off and anonymized data used. Overall, the study addresses an important topic and contributes valuable regional data. However, substantial revision is necessary to improve the methodological rigor, transparency of reporting, and interpretation of findings. I would recommend major revision before publication

**Reviewer #3:**  ICU admission and mortality in adult patients with influenza A/H1N1-related pneumonia in Vietnam is a well researched and an original paper presenting the ICU admissions of H1N1 pneumonia an important factor causing death in young adults as well as elderly. Epidemiological data has been well researched retrospectively inspite of low sample siz.

**********

what does this mean?). If published, this will include your full peer review and any attached files.

If you choose “no”, your identity will remain anonymous but your review may still be made public.

**Do you want your identity to be public for this peer review?**  For information about this choice, including consent withdrawal, please see our Privacy Policy

Reviewer #1: No

Reviewer #2: No

Reviewer #3: No

**********

[NOTE: If reviewer comments were submitted as an attachment file, they will be attached to this email and accessible via the submission site. Please log into your account, locate the manuscript record, and check for the action link "View Attachments". If this link does not appear, there are no attachment files.]

To ensure your figures meet our technical requirements, please review our figure guidelines: https://journals.plos.org/plosone/s/figures

You may also use PLOS’s free figure tool, NAAS, to help you prepare publication quality figures: https://journals.plos.org/plosone/s/figures#loc-tools-for-figure-preparation.

NAAS will assess whether your figures meet our technical requirements by comparing each figure against our figure specifications.

Attachment Submitted filename: Review H1N1 Pneumonia Article .docx

Attachment Submitted filename: Reviewer comments H1N1 Plosone.odt

Attachment Submitted filename: PONE-D-26-17842_reviewer comments.docx

# Author comment: Author response to Decision Letter 1

**DOI:** 10.1371/journal.pone.0348450.r002

16 Jul 2026

Handling Editor’s comments:

1. Given that influenza circulates year-round in Vietnam, both Southern and Northern hemisphere influenza vaccines together with a locally manufactured seasonal influenza vaccine are licensed. The authors should introduce the seasonality of influenza A in Southern and Northern hemisphere, as well as epidemiology of influenza and co-infection with other respiratory viruses in the post-COVID era. More references should be cited, with this one (PMID: 40137747) as an example (citing is optional).

Response: We thank the Editor for this valuable suggestion. We agree that additional background on influenza seasonality, post-COVID epidemiology, and viral co-infection strengthens the rationale for our study. Accordingly, we have revised the Introduction by incorporating recent evidence on global influenza circulation, changes in influenza epidemiology following the COVID 19 pandemic, and the clinical relevance of influenza and SARS-CoV-2 co-infection. The revised text is shown below.

Introduction – 1st paragraph

While influenza seasonality is well defined in temperate regions, circulation patterns in tropical areas are often irregular, which may compromise the optimal timing and impact of vaccination programs [5]. Despite the availability of effective vaccines, A(H1N1)pdm09 remains a significant contributor to worldwide influenza-related morbidity and mortality [6]. From October 2024 through May 2025 influenza activity was reported in all influenza transmission zones [7]. The predominant viruses varied across transmission zones and between countries [7]. A study conducted in China found that between 2010 and 2015, the influenza-associated mortality was 9.9 per 100,000 people, in which influenza A(H3N2) virus was the leading cause with a rate of 5.18 per 100,000, followed by A(H1N1)pdm09 with a rate of 2.9 per 100,000[8]. Additionally, the prevalence of influenza shifted between the Southern and Northern Hemispheres following the COVID-19 pandemic. Specifically, the post-pandemic peak of influenza in the Northern Hemisphere occurred as early as December, with the epidemic's duration increasing to 11 weeks. Meanwhile, in the Southern Hemisphere, the epidemic increased in length to 20 weeks [9]. Given that influenza A/B and SARS-CoV-2 share similar transmission pathways (via aerosolized droplets and contaminated surfaces) and clinical presentations, and typically circulate in overlapping seasonal outbreaks, their potential for co-infection poses a significant public health threat [9].

2. Lines 129-137 "Nasal and oropharyngeal swabs were collected ... All laboratory tests were performed at the standardized Laboratory Department (ISO 15189) of the HTD." The methods section describes influenza A and influenza B detection using PCR, but it does not provide influenza A subtype (typing) information. In addition, the authors should introduce that LAMP has been successfully used for the detection of influenza A virus. More references should be cited, with this one (Liu, B.M. Isothermal nucleic acid amplification technologies and CRISPR-Cas based nucleic acid detection strategies for infectious disease diagnostics. p 30-47. In Manual of Molecular Microbiology: Fundamentals and Applications; ASM Press: Washington, DC, USA, 2025.doi:10.1128/9781683674597.ch03.) as an example (citing is optional).

Response: We thank the Editor for this helpful suggestion. We have revised the Materials and Methods section to clarify the influenza A subtyping procedure used in this study. We have also briefly acknowledged Loop Mediated Isothermal Amplification (LAMP) as an emerging molecular diagnostic method for influenza A, while noting that real-time RT PCR remained the routine diagnostic platform throughout the study period. The revised text is shown below.

Materials and methods - A(H1N1)pdm09 laboratory confirmation, definitions of A(H1N1)pdm09 pneumonia, and indications for ICU admission paragraph

Nasal and oropharyngeal swabs were collected by medical staff as per the HTD’s guidelines. Loop Mediated Isothermal Amplification (LAMP) has also been reported as a rapid molecular method for detecting influenza A virus [25]. However, throughout the study period, real-time RT PCR remained the routine diagnostic method at our institution and was therefore used for patient enrolment. RT-PCR was performed on the LightCycler 480 II System (Roche Molecular Diagnostics, Pleasanton, CA) [26, 27] using the QIAGEN OneStep RT‐PCR kit [27, 28].

Reviewer 1’s comments:

1. Overall Evaluation

This manuscript presents a 10-year retrospective cohort study evaluating ICU admission, mortality, and predictors of severe outcomes among adults with A(H1N1)pdm09 pneumonia in Vietnam. The study addresses an important public health issue, particularly in tropical and resource-limited settings where long-term influenza surveillance data are limited. The long study duration and inclusion of real-world clinical data are notable strengths.

The manuscript is clinically relevant and provides useful epidemiological and outcome data from a tropical Asian setting. However, several methodological, statistical, and presentation-related issues should be addressed before publication. Some conclusions are currently overstated relative to the observational nature of the study and the limited sample size, especially regarding antiviral therapy and mortality.

Overall recommendation: Major Revision

Response: We sincerely appreciate your evaluation and constructive feedback. We are grateful for the recognition of our study's clinical relevance and its contribution to filling surveillance gaps in tropical settings.

We have taken your concerns very seriously and have comprehensively addressed the methodological, statistical, and presentation-related issues in this revised manuscript. All individual queries have been answered in detail below.

2. Clarify Study Objective and Primary Outcomes

The objectives should be more explicitly stated in the Introduction. Currently, the manuscript discusses ICU admission, mortality, and treatment outcomes, but the primary and secondary outcomes are not clearly defined.

Suggested improvement:

At the end of the Introduction, clearly state:

• Primary outcomes: ICU admission and mortality

• Secondary outcomes: ventilation requirement, ARDS, hospital-acquired infection, and length of stay

Response: We thank the Reviewer for this excellent suggestion. While these endpoints were initially detailed in the Materials and Methods section, we agree that explicitly defining them at the end of the Introduction enhances the clarity of our study objectives. We have revised the final paragraph of the Introduction as follows:

Introduction – 3rd paragraph

To strengthen influenza prevention and control in Vietnam and similar settings, continual assessment of disease severity is needed [22]. This study aimed to examine the clinical burden and identify predictors of severe disease among adult patients with A(H1N1)pdm09-related pneumonia. The primary outcomes were ICU admission and all-cause mortality. The secondary outcomes included respiratory support requirements (such as invasive ventilation), development of moderate-to-severe ARDS, occurrence of hospital-acquired infections, and overall hospital and ICU lengths of stay.

3. Concern Regarding Small Number of Mortality Events

The mortality analysis appears statistically underpowered. Only 13 deaths occurred, yet many variables were entered into the multivariable logistic regression model. This raises concern for:

• Model overfitting

• Unstable estimates

• Extremely wide confidence intervals

• Reduced reliability of adjusted odds ratios

Examples:

• Invasive ventilation AOR 55.355 (95% CI 1.486–2062.375)

• Double-dose oseltamivir/combination therapy AOR 32.625 (95% CI 1.594–667.661)

These wide confidence intervals indicate substantial statistical instability.

Suggestions:

• Reduce the number of variables included in the mortality regression model.

• Consider using penalized logistic regression or Firth logistic regression.

• Include an events-per-variable justification.

• Acknowledge possible model instability more explicitly in limitations.

Response: We thank the Reviewer for raising this important statistical concern. We acknowledge that the small number of deaths (n = 13) limits the precision of the mortality model and increases the risk of model overfitting. Variable selection followed a pre-defined purposeful selection strategy based on both statistical criteria (P < 0.25 in the univariable analysis) and clinical relevance, as recommended in the methodological literature. We retained clinically important variables to reduce potential confounding, but we also recognised that the limited number of events resulted in wide confidence intervals for several estimates. Based on your suggestion, we used Firth logistic regression to strengthen the power of mortality. Additionally, we have included model diagnostics appropriate for each multivariable model. For the ICU admission model, calibration was assessed using the Hosmer–Lemeshow goodness-of-fit test, and discrimination was evaluated using the area under the receiver operating characteristic curve (AUC). For the mortality model, which was analysed using Firth's penalized logistic regression, model performance was evaluated using the penalized likelihood ratio test together with the AUC. Multicollinearity was assessed using variance inflation factors (VIFs), and no evidence of problematic multicollinearity was identified among the variables included in the mortality model. Despite these strategies, we acknowledge that the number of covariates remains high relative to the number of events and therefore regard the mortality model as exploratory. In response to this, we further strengthened the Limitations section to explicitly acknowledge the exploratory nature of the mortality model and the possibility of model instability. The revised text is shown below:

Materials and methods – statistical analysis paragraph

“…A standard multivariable logistic regression for ICU admission prediction model was constructed. Model calibration and goodness-of-fit were formally evaluated using the Hosmer–Lemeshow test, while discrimination capacity was assessed via the Area Under the ROC Curve (AUC / C-statistic). Multicollinearity among predictors was monitored using Variance Inflation Factors (VIF). For mortality model, due to low-event constraints (sparse mortality data), Firth’s penalized logistic regression was employed to minimize small-sample bias and avoid issues of separation. Complete-case analysis was applied to this model. Goodness-of-fit was evaluated using the Penalized Likelihood Ratio Test, and discrimination power was determined via the AUC / C-statistic. Multicollinearity was similarly assessed using VIF. Given the potential for confounding among covariates, the purposeful selection process was used to identify covariates for the regression models. Following the methodology described elsewhere [34, 35], a more generous P-value cutoff of <0.25 in the univariate analysis was used. This approach was chosen because traditional cutoffs (such as 0.05) often fail to identify variables that, while not independently significant, act as important confounders or become significant when adjusted for other covariates. Furthermore, variables considered clinically important based on the authors' expertise were retained irrespective of their univariable statistical significance to ensure that potential confounding was appropriately addressed.”

Results

Table 7. Firth's penalized logistic regression analysis of predictors of mortality among 158 patients with laboratory-confirmed A(H1N1)pdm09 pneumonia

To ensure complete transparency and help readers better interpret our findings, we have expanded the Limitations section to explicitly address how the small number of events affects model precision and stability:

Discussion – Limitations paragraph

“Our study has some notable limitations. Firstly, this study was based on an analysis of medical records and thus, data such as patients’ BMI [63] that may confound the study findings have not been documented. Additionally, some predictor variables contained missing data. Variables with substantial missingness were excluded from the multivariable analyses, which may have limited our ability to evaluate the effects of some potentially relevant predictors. Importantly, individual influenza vaccination status was not recorded in the historical medical records. Since vaccination can significantly reduce clinical severity, its absence may have confounded the observed associations with ICU admission, disease severity, and mortality. Secondly, although our study included all eligible patients treated during the study period, only 13 deaths occurred. Consequently, the multivariable mortality model should be interpreted cautiously, as the limited number of outcome events may have resulted in model overfitting and imprecise effect estimates, reflected by the wide confidence intervals for several adjusted odds ratios. Systematic microbiological investigations for bacterial co-infection at hospital admission were not performed for all patients. Therefore, the potential contribution of community acquired bacterial co-infection to disease severity and mortality could not be evaluated in this study. In addition, although the institutional criteria for ICU admission remained unchanged throughout the study period, we cannot completely exclude temporal changes in clinical practice, supportive care, or diagnostic approaches that may have influenced patient management over the 10-year study period. Finally, because the Hospital for Tropical Diseases (HTD) serves as a specialized tertiary referral center, our cohort is inherently prone to selection bias toward more severe clinical cases. Therefore, the reported ICU admission rates and mortality estimates may be higher than those in community hospital settings and may not fully represent the broader population with general influenza infections…”

4. Interpretation of Antiviral Therapy Findings is Overstated

The manuscript repeatedly implies that double-dose oseltamivir or combination therapy may increase mortality. However, this conclusion is not sufficiently supported because:

• This is an observational retrospective study.

• Sicker patients are more likely to receive aggressive therapy (confounding by indication).

• Disease severity itself likely influenced treatment decisions.

Therefore, the association should not be interpreted causally.

Current problematic statements:

• “Neither double-dose oseltamivir nor combination therapy is recommended…”

• “Alternative antiviral regimens should not be recommended.”

These conclusions exceed the evidence generated by this study.

Suggested revision:

Replace with more cautious wording such as:

“The observed association between intensified antiviral therapy and mortality likely reflects treatment allocation to more severely ill patients rather than a direct harmful effect of therapy.”

And:

“Further prospective studies are required to evaluate the efficacy of intensified antiviral regimens in severe influenza.”

Response:

We thank the Reviewer for this important comment. We agree that the observed association should not be interpreted as causal because this retrospective observational study is susceptible to confounding by indication. Therefore, we have revised the Discussion and Conclusion to avoid causal language and to emphasise that patients receiving intensified antiviral therapy were likely those with more severe disease. The revised text is shown below.

Discussion – 6th paragraph

“…The observed association between intensified antiviral therapy and mortality most likely reflects confounding by indication. Patients with more severe disease were preferentially treated with intensified antiviral regimens rather than a direct harmful effect of the therapy itself. Current evidence does not demonstrate a clear survival benefit from intensified antiviral regimens in severe influenza, and prospective studies are needed before definitive conclusions regarding their clinical effectiveness can be drawn…”

5. ICU Admission Criteria Need Be

Attachment Submitted filename: Response to reviewers and editor 14072026 .pdf

# Editor report: Decision Letter 1

**DOI:** 10.1371/journal.pone.0348450.r003

**Authors:** Benjamin M. Liu

20 Jul 2026

<p>ICU admission and mortality in adult patients with influenza A(H1N1)pdm09-related pneumonia in Vietnam since the 2009 H1N1 pandemic: a 10-year cohort study

PONE-D-26-17842R1

Dear Dr. Bui,

We’re pleased to inform you that your manuscript has been judged scientifically suitable for publication and will be formally accepted for publication once it meets all outstanding technical requirements.

Within one week, you’ll receive an e-mail detailing the required amendments. When these have been addressed, you’ll receive a formal acceptance letter and your manuscript will be scheduled for publication.

An invoice will be generated when your article is formally accepted. Please note, if your institution has a publishing partnership with PLOS and your article meets the relevant criteria, all or part of your publication costs will be covered. Please make sure your user information is up-to-date by logging into Editorial Manager at Editorial Manager® and clicking the ‘Update My Information' link at the top of the page. For questions related to billing, please contact billing support.

If your institution or institutions have a press office, please notify them about your upcoming paper to help maximize its impact. If they’ll be preparing press materials, please inform our press team as soon as possible -- no later than 48 hours after receiving the formal acceptance. Your manuscript will remain under strict press embargo until 2 pm Eastern Time on the date of publication. For more information, please contact onepress@plos.org.

Kind regards,

Benjamin M. Liu, PhD, D(ABMM), MB(ASCP)

Academic Editor

PLOS One

Additional Editor Comments (optional):

Reviewers' comments:

# Editor report: Acceptance letter

**DOI:** 10.1371/journal.pone.0348450.r004

**Authors:** Benjamin M. Liu

PONE-D-26-17842R1

PLOS One

Dear Dr. Bui,

I'm pleased to inform you that your manuscript has been deemed suitable for publication in PLOS One. Congratulations! Your manuscript is now being handed over to our production team.

At this stage, our production department will prepare your paper for publication. This includes ensuring the following:

* All references, tables, and figures are properly cited

* All relevant supporting information is included in the manuscript submission,

* There are no issues that prevent the paper from being properly typeset

You will receive further instructions from the production team, including instructions on how to review your proof when it is ready. Please keep in mind that we are working through a large volume of accepted articles, so please give us a few days to review your paper and let you know the next and final steps.

Lastly, if your institution or institutions have a press office, please let them know about your upcoming paper now to help maximize its impact. If they'll be preparing press materials, please inform our press team within the next 48 hours. Your manuscript will remain under strict press embargo until 2 pm Eastern Time on the date of publication. For more information, please contact onepress@plos.org.

You will receive an invoice from PLOS for your publication fee after your manuscript has reached the completed accept phase. If you receive an email requesting payment before acceptance or for any other service, this may be a phishing scheme. Learn how to identify phishing emails and protect your accounts at https://explore.plos.org/phishing.

If we can help with anything else, please email us at customercare@plos.org.

Thank you for submitting your work to PLOS ONE and supporting open access.

Kind regards,

PLOS ONE Editorial Office Staff

on behalf of

Dr. Benjamin M. Liu

Academic Editor

PLOS One
